# Liberia health system's journey to long-term recovery and resilience post-Ebola: a case study of an exemplary multi-year collaboration

**DOI:** 10.3389/fpubh.2023.1137865

**Published:** 2023-06-19

**Authors:** Louis Ako-Egbe, Redda Seifeldin, Sohel Saikat, Sanford C. Wesseh, Moses Brown Bolongei, Ballah Jusu Ngormbu, Roseline George, Charles Ocan, Clement Lugala Peter Lasuba

**Affiliations:** ^1^Health Systems Strengthening, World Health Organization - Country Office for Liberia, Monrovia, Montserrado, Liberia; ^2^World Health Organization, Geneva, Switzerland; ^3^Ministry of Health Liberia, Monrovia, Liberia; ^4^World Health Organization - Country Office for Liberia, Monrovia, Montserrado, Liberia; ^5^National Public Health Institute of Liberia, Monrovia, Montserrado, Liberia

**Keywords:** health systems, resilience, integration, catchment approach, emergency preparedness and response, healthcare quality, recovery, COVID-19

## Abstract

This article is part of the Research Topic ‘Health Systems Recovery in the Context of COVID-19 and Protracted Conflict'

Liberia is one of the three countries worst hit by the 2014–2016 West Africa Ebola Virus disease (EVD) outbreak, during which it recorded over 10,000 cases, including health workers. Estimates suggest that the non-EVD morbidity and mortality resulting from the collapse of the health system exceeded the direct impact of EVD. Lessons from the outbreak were clear, not only for Liberia but also for the regional and global communities: that building health system resilience through an integrated approach is an investment in population health and wellbeing, as well as economic security and national development. It is therefore no surprise that Liberia made recovery and resilience a national priority from the time the outbreak waned in 2015. The recovery agenda provided the platform for stakeholders to work toward the restoration of the pre-outbreak baseline of health system functions while aiming to build a higher level of resilience, informed by lessons from the Ebola crises. Based on the co-authors' experiences of on-the-ground country-support work, this study sought to provide an overview of the Liberia Health Service Resilience project (2018–2023) funded by KOICA, and propose a set of recommendations for national authorities and donors, derived from the authors' perceptions of best practices and key challenges associated with the project. We used both quantitative and qualitative approaches to generate the data represented in this study by reviewing published and unpublished technical and operational documents, and datasets derived through situational and needs assessments and routine monitoring and evaluation activities. This project has contributed to the implementation of the Liberia Investment Plan for Building a Resilient Health System and the successful response to the COVID-19 outbreak in Liberia. Although limited in scope, the Health Service Resilience project has demonstrated that health system resilience could be operationalized by applying a catchment and integrated approach and encouraging multi-sectoral collaboration, partnership, local ownership, and promoting the Primary Health Care approach. Principles applied in this pilot could guide the operationalization of health system resilience efforts in other resource-limited settings similar to Liberia and beyond.

## Introduction

Liberia is one of the three countries worst hit by the 2014–2016 West Africa Ebola Virus disease (EVD) outbreak, during which it recorded over 10,000 cases, including health workers. Estimates suggest that the non-EVD morbidity and mortality resulting from the collapse of the health system exceeded the direct impact of EVD (1). The health and socioeconomic impacts of the outbreak were felt by individuals, communities, governments, and organizations long after the outbreak. Lessons from the outbreak were clear, not only for Liberia but also for the regional and global communities: that building health system resilience through an integrated approach (2) is an investment in population health and wellbeing, as well as in economic security and national development. It is, therefore, no surprise that Liberia made recovery and resilience a national priority in 2015 when the outbreak began to wane (3). The recovery agenda provided the platform for stakeholders to work toward the restoration of the pre-outbreak baseline of health system functions while aiming to build a higher level of resilience, informed by lessons from the Ebola crises.

The health system was ill-equipped to effectively respond to EVD with the necessary occupational health, safety, infection prevention, and control (IPC) measures for safe and effective health services. As a result, health workers suffered a 30 times higher risk of infection compared to the general population. As of April 8, 2015, 372 health workers had been infected, of whom 184 died (3). Pre-existing (pre-EVD) system vulnerabilities, partly due to the impact of a 14-year civil war in Liberia, contributed to the aftermath of EVD. Such vulnerabilities included inadequate and poorly motivated health workers, insufficient and unsuitable infrastructure and equipment, a weak supply chain system, and poor overall quality of care. These contributed to around 50% disruption in the delivery of routine health services during EVD due to health facility closures, fears and refusal of health workers to provide routine health services, and community distrust and fears (3). Coverage of life-saving maternal and child health interventions, in particular, declined dramatically, from already low levels (3).

Table 1 presents key health system indicators in the pre- and post-EVD era (4, 5).

**Table 1:** Health system indicators pre- and post-EVD.

**Health indicators**	LDHS^*^, 2007	**LDHS, 2019–20**
Total Fertility Rate (TFR), children per woman	5.2	4.2
Use of modern family planning method by married women (15–49 years)	10%	24%
Antenatal care provided by skilled health workers	79%	98%
Skilled assistance during delivery	46%	84%
Institutional deliveries	37%	80%
Children 12–23 months with full immunization coverage	39%	51%
Stunting in under-five children	39%	30%
Wasting in under-five children	8%	3%%
Insecticide-treated nets ownership (household)	47%	55%
Insecticide-treated Net use (pregnant women 15–49 years)	47%	78%
Insecticide-treated Net use (children under five)	26%	44%
Children under-5 with fever receiving malaria treatment	45%	85%
Pregnant women receiving intermittent preventive therapy (IPT)	58%	90%
Comprehensive knowledge of HIV/AIDS (female)	19%	33%
Comprehensive knowledge of HIV/AIDS (male)	32%	35%
Pregnancy-related mortality ratio (100,000 live births)	994	913
Maternal mortality ratio (maternal deaths per 100,000 live births)	1,072	742
Under-five mortality rate (deaths per 1000 live births)	110	93
Infant mortality (per 1000 births)	71	63
Child mortality (per 1000 births)	41	33

^*^Source: Liberia Demographic Health Survey (LDHS) 2019–2020.

Liberia's 2015–2021 Investment Plan for Building a Resilient Health System (3) was developed to support the transition of the health system from EVD response to recovery and the “building back better” of a health system that provided quality routine services while also remaining resilient to future outbreaks. More specifically, the plan aimed to improve the health of Liberians and achieve equitable health outcomes by improving access to safe and quality services, establishing a robust Health Emergency Risk Management System, and building an enabling environment that restored trust in the health system's ability to provide services (3). The plan identified nine key investment areas, namely, building a fit-for-purpose health workforce, re-engineering the health infrastructure, strengthening Emergency Preparedness and Response (EPR) and surveillance, restoring and enhancing service delivery systems, enhancing capacity for essential medicines and supplies, strengthening information and research systems, expanding capacity for leadership and governance, establishing sustainable financing systems, and establishing a professional community workforce (3).

The ultimate goal of the Investment Plan was to enhance health system resilience. The resilience of a health system is the capacity of health actors, institutions, and populations to prepare for and effectively respond to crises; maintain core functions when a crisis hits; and informed by lessons learned during the crisis, reorganize if conditions require (2). An integrated approach (6) to develop and sustain the quality of routine services and emergency preparedness in tandem was emphasized in the Investment Plan. However, operationalizing this integrated approach in the Liberia health system remained a gap (7).

A 5-year (2018–2023) Health Services Resilience (HSR) project, funded by the Korea International Corporation Agency (KOICA) through the Global Disease Eradication Fund (GDEF), was approved for implementation in Liberia in October 2018 with the aim of “Making Health Services Resilient with Quality and Preparedness for Emergency Response in Liberia”. A parallel project, also funded by KOICA, is implemented in Ethiopia. Liberia's project sought to contribute to the implementation of priority activities of the Investment Plan, especially building a fit-for-purpose health workforce that equitably and optimally delivers quality services (Investment Plan Area A and Project Outcome 3). The project leveraged the momentum for focusing on resilience; structures established post-Ebola through the Investment Plan, such as the establishment of the Quality Management Unit within the Ministry of Health (MoH); and aspirations to finalize and roll out a National Quality Strategy.

An in-depth situational assessment (SA) of the state of quality and resilience in Liberia post-EVD (7) foregrounded work on the HSR project. The SA revealed a general weakness in human resource capacity in healthcare quality and resilience and a paucity of associated technical tools (guidelines, plans, strategies, and sets of indicators). It also found a lack of integration of the metrics used for healthcare quality and emergency preparedness in health services. The SA equally highlighted the need for a systematic and integrated approach to improving the capacity of health workers and health system leaders in quality and resilience by ensuring their active participation in activities related to health service quality and preparedness for public health emergencies.

The Health Services Resilience project set out to provide catalytic support (financial and technical) to the Ministry of Health (MoH), the National Public Health Institute of Liberia (NPHIL), and other relevant institutions, coordinated by the World Health Organization (WHO), to address the gaps identified from the SA and enhance the implementation of the Investment Plan. The project also aimed to operationalize the concept of resilience at the policy and operational levels of the health system by applying the key lessons learned from shock experiences, with such lessons essential for the recovery and transformation of a health system post-crisis (6).

## Aims and objectives

This study aims to:

- Provide an overview of the Liberia HSR project (2018–2023) funded by KOICA, with a focus on its guiding principles, key features of its set-up, main activities, achievements, best practices, and challenges.
- Explore the contribution of the HSR project to the implementation of the Investment Plan for Building a Resilient Health System in Liberia and broader health system and population health outcomes in Liberia.
- Propose a set of recommendations for national authorities and donors, derived from the authors' perceptions of best practices and key challenges associated with the project, with a focus on “building back better” health systems post-COVID-19 and on strengthening preparedness for future public health events.

Insights from this case study would be useful for learning from Liberia's experience in long-term recovery efforts and the benefits of investing in more integrated, system-strengthening, resilience-focused initiatives. This is particularly timely as many countries are embarking on recovery from the ongoing COVID-19 pandemic and other health system shocks.

## Methods

The sources of data represented in this study comprise official reports from the HSR project, published and unpublished technical and operational documents from it, and datasets generated through situational and needs assessments and routine monitoring and evaluation activities. In this section, we describe the methodological steps specific to the current study, namely how we identified documents for review and how findings from them were extracted and synthesized. Sections “Approach to developing and refining the project goals, tools and activities” and “Monitoring and Evaluation” under “Overview of the HSR project” provide detail on data collection methods used to serve the needs of the HSR project development, implementation, and monitoring and evaluation. Relative to the current study, these processes fed in ready data which we only synthesized. As the original goals served by such data were strongly operational, and also somewhat different from the goals of the current study, the pre-existing data we use have significant limitations. These are discussed under the Section *Study strengths and limitations* at the end of the article.

### Identification and prioritization of documents and data sources

To identify the richest and most reliable documents that can be used to construct an overview of Liberia's HSR project (study goal 1), LA and RS first reviewed all major project folders and developed a draft list of key project-generated documents (Table 2). The list was supplemented by titles of documents concerning the Investment Plan for Building a Resilient Health System and broader health system and population health outcomes (3, 4, 8–13) in Liberia (study goal 2). These were identified through online searches in PubMed, Google Scholar and Google by using search terms such as “public health emergency,” “health care quality,” “service resilience,” “health system resilience,” and “resilience capacities,” and through soliciting advice from MoH colleagues. The combined draft list was then enriched with recommendations made by stakeholders at MoH, NPHIL, WHO, and further relevant institutions and partners [United Nations International Children's Emergency Funds-UNICEF, USAID-supported John Hopkins Program for International Education in Gynecology and Obstetrics-, Jhpiego and Last Mile Health (LMH)]. The process was iterative and resulted in the final list presented in Table 2. All documents from the list were then retrieved, including the most recent drafts of un-finalized policies and plans.

**Table 2:** List of documents consulted for the desk review.

**Documents retrieved**	**Year of publication**	**Data extracted**
**National policies, plans, strategies, and guidelines**		
•1. Liberia National Health Policy, 2022–2031• 2. National Health Sector Strategic Plan: A Roadmap to Universal Health Coverage, 2022–2026• 3. Essential Package of Health Services for Universal Health Coverage, Liberia 2022• 4. Liberia Essential Package of Health Services (EPHS II)• 5. Investment Plan for Building a Resilient Health System in Liberia, 2015–2021 • 6. Liberia Demographic and Health Survey, 2019–2020• 7. Liberia Harmonized Health Facility Assessment Report, October 2022• 8. Liberia National Health Quality Strategy (draft) 2023–2027	2022 2022 2022 2022 2015 2021 2023	- Health system profile of Liberia - Health system indicators and demographic characteristics - Health system priorities, including investment areas for building a resilient health system - Objectives of the Investment Plan for Building a Resilient Health System in Liberia
**Project technical documents**		
1. Operational Framework and Technical Guide. Korean International Cooperation Agency (KOICA)-Funded Project: Making Health Services Resilient with Quality and Preparedness for Emergency Response in Ethiopia and Liberia 2. Liberia Situational Assessment Report. KOICA-Funded Project: Making Health Services Resilient with Quality and Preparedness for Emergency Response 3. KOICA-Funded Project work plan and Monitoring and Evaluation Framework 4. Off-The-Shelf Exercise Handbook. Health Systems Resilience Exercises 5. Training Package on Integrated Approach to Health System Resilience Focusing on Services 6. Health Service Resilience Indicators (Including Adaptation for Primary Health Care Monitoring and Evaluation) 7. Stakeholders' Consultative Meeting Report Korea International Cooperation Agency (KOICA)-Funded Health Service Resilient Project, 8–10 October 2029	2019 2019 2019 2019 2020 2020 2019	- Project guiding principles - Project catchment approach - Project pilot sites (counties, districts, and facilities) - Project outcomes, outputs, activities, and indicators - Health Service resilience indicators
**Project activity reports**		
1. Health Service Resilience (KOICA-Funded) Project: Making Health Services Resilient with Quality for Emergency Preparedness and Response in Liberia. Mid-term Review Workshop Report (2–3 September 2021) 2. Integrated training in IPC, WASH, and COVID-19 Case Management for Frontline Healthcare Workers in Lofa, Bong, and Grand Cape Mount Counties (28–30 April 2020) 3. Training of border parties to strengthen disease surveillance and referral pathways at Ground Crossing Points of Entry in Lofa and Grand Cape Mount Counties (21–23 April 2020) 4. Progress Report. Application of the HS SimEx Package to review the functionality of sub-national Health System to respond to COVID-19 and continue Essential Health Services 5. Strengthening maternal newborn and child health QoC in health facilities through training and supportive supervision in 3 project counties (8–14 November 2020) 6. Strengthening health facility Quality Management Teams to enhance routine health services and during outbreaks (COVID-19): Montserrado, Bomi, Gbarpolu, Grand Cape Mount, Margibi, and Nimba Counties (14–20 February 2021) 7. Strengthening Infection Prevention and Control Standards and Practices in Health Facilities through Supportive Supervision/on-site Mentorship to promote Quality Healthcare Delivery, September 2020 8. Health Workforce Competency Assessment on Quality and Resilience, August 2020 9. MoH Annual Operational Plan for Fiscal Year 2020/2021 10. Progress Report. Strengthening the Quality of Care in routine service delivery for public health emergency and response through teleconference with National and County Quality Management Teams, November 2020 11. Baseline assessment of Antibiotic Consumption and Resistance using the Point Prevalence Survey. Assessment in seven hospitals from four counties in Liberia 12. Mentorship on safe healthcare waste management and restoration of water supply for quality and health service delivery at Tellewoyan hospital, September 2020 13. Training of Frontline Healthcare Workers and Health Systems Managers in Healthcare Quality and Resilience for Emergency Preparedness and Response in Lofa and Bong Counties, 12–16 December 2022 and 19–23 January 2023 14. Pre-validation Workshop for WISN Results for Clinics and Health Centers, 12–13 December 2022 15. Pre-validation Workshop for the National Health Quality Strategy (2022–2026) for Liberia, 24–25 November 2023	2021 2020 2020 2021 2020 2020 2020 2020 2020 2020 2020 2020 2022 2022	- Project achievements and results - Project best practices, strengths, challenges, and limitations

### Approach to data extraction and synthesis

LA and RS independently reviewed the selected documents for data that can serve to address the study objectives. Data were extracted in Microsoft Word. Table 1 (third column) shows what types of data were extracted from the different categories of documents. The process of considering the relevance of data and representing and prioritizing it was iterative, with key stages involving all co-authors. Data were synthesized following recommendations made by Lin et al. (14) on using health system data in combination with systematic reviews to support decision-making.

## Findings

### Overview of the HSR project

Liberia's Health Services Resilience project was designed for implementation over 5 years (October 2018–October 2023) and had a clear work plan comprising five outcomes and 15 outputs as well as a monitoring and evaluation framework (Box 1) (15). These were all agreed upon and jointly implemented by key stakeholders from global, national, sub-national, and service delivery levels. This section offers an overview of the project in terms of its guiding principles; key stakeholders; settings; approaches to the development and implementation of goals, tools, and activities; and monitoring and evaluation framework.

Box 1 **Overall goal and summary of expected outcomes of the HSR project.**

To build resilience in health systems to enable quality health services in all contexts, along with preparedness.

✓ Build health service resilience with systematic consideration of quality and emergency preparedness for response including health services continuity
✓ Develop bridges between work on health systems (primarily MoH responsibility) and health security (primarily NPHIL responsibility) as well as between the human health sector and allied sectors (such as animal health).
✓ Promote effective health system participation in emergency preparedness and response

The Korean International Cooperation Agency (KOICA) funded the project, through the Global Disease Eradication Fund (GDEF).

#### Guiding principles

The following key principles underpinned all project activities and tools:

- **Government ownership and leadership**. All project activities are implemented with the leadership of responsible local and national health authorities. This includes the participation of the Minister of Health and Director General (DG) of NPHIL in project workshops and their guidance on the implementation of project activities.
- **Local, national, and international stakeholders' participation and collaboration**. The project team applies a collaborative and coordinated approach with national and international stakeholders at all administrative levels to avoid duplication and fragmentation of efforts. A “One work plan, One budget” approach is used to enhance collaboration, transparency, and efficiency in project implementation.
- **“One WHO” support**. The skills and experience at all three levels of WHO (Country Office, Regional Office for Africa, and Headquarters) have been harnessed in the project development and implementation.
- **Catchment approach**. Project implementation involves actors responsible for strengthening service delivery and emergency preparedness and response at the four levels of the health system (community/health facility, health district, county, and national levels) and from the animal and environmental health sectors, in addition to the human health one (One-Health umbrella) (16).
- **Primary healthcare focus**. The project prioritizes high-impact activities at the community and primary health facility level in line with the Essential Package of Health Services (EPHS) (8).
- **Integration/system approach**. Synergies between health system strengthening and public health emergency efforts are sought, including enabling health services and multi-sectoral participation across all levels (national to sub-national) toward Universal Health Coverage (UHC) and health security as interdependent goals. The project bridged health systems and health security efforts during health sector planning, COVID-19 preparedness and response, After Action Reviews (AAR), and Simulation Exercise (SimEx) by promoting the participation of stakeholders from health services, animal health, and environmental health.
- **Multi-sectorial engagement**. The project fosters joint working among MoH, NPHIL, and other relevant sectors, such as the Ministry of Agriculture (MoA), Environmental Protection Agency (EPA), Ministry of Internal Affairs (MIA), Port Health Authorities, and academia, especially at the sub-national level.
- **Partnership and sustainability**. Collaboration with health sector partners is paramount. For example, collaboration with the USAID-funded STAIP (Strategic Technical Assistance for Improved Health System Performance and Health Outcomes) and BRIDGE-U (Bringing Research to Impact for Development, Global Engagement and Utilization) projects resulted in the implementation of county operational planning and completion of the national Guideline for Continuing Professional Development (CPD). In addition, partnership with Jhpiego (the John Hopkins Program for International Education in Gynecology and Obstetrics) and Last Mile Health (LMH) led to the development of the new National Health Quality Strategy.

#### Key stakeholders

The country-based project team comprises two dedicated project staff from the WHO country office and two project focal persons from MoH (health systems) and NPHIL (health security), jointly implementing the project with support from the WHO Headquarters and Regional Office team. The two dedicated project officers from WHO Liberia Country Office are qualified and experienced public health practitioners and quality improvement specialists. The national focal person from the MoH is an experienced physician and director of the Health Quality Management Unit, while that from NPHIL is the director of training for the institute. Table 3 lists key stakeholders working closely with the WHO project team and national focal points.

**Table 3:** Examples of key stakeholders working closely with the WHO project team to implement the project.

**Level**	**Stakeholders/organization**	**Position and role**
National	- MoH: Health Quality Management Unit (HQMU) and related departments such as Health Monitoring, Evaluation and Research (HMER), Policy and Planning Unit, Family Health Division, Information Communication and Technology (ICT), and Human Resource for Health (HRH) units. - Ministry of Agriculture (MoA) - Environmental Protection Agency (EPA)	Leadership (Assistant Ministers and Directors) and technical focal points
	National Public Health Institute of Liberia (NPHIL)	Leadership (DGs) and technical focal points
	WHO country office (Health Systems and Health emergency clusters)	Leadership (WHO Country Representative (WR), Health Systems Strengthening (HSS), and WHO Health Emergency (WHE) team leads and technical focal points
	Health professional councils *(in charge of continuous education, licensing, accreditation, and registration)*	Nursing and midwifery board officers
	NGOs/Partners implementing quality and emergency initiatives such as Last Mile Health (LMH), Johns Hopkins Program for International Education in Gynecology and Obstetrics (Jhpiego), GIZ, USAID, UNICEF, and UNFPA	Leadership and technical focal points
Sub-national	County health teams	County Health Officer, Community Health Department Director, County Surveillance Officer, other technical officers (Infection Prevention and Control (IPC), Child Health Survivor, Maternal health), County authorities, and County Veterinary Officers
	District health teams	District Health Officer, District Surveillance Officer, and Leads and technical officers (IPC, Child Health Survivor Environmental Health Technician)
Health facility and community	County/district hospitals Health centers Primary health clinics *(public, private, and faith-based)*	Heads or focal persons responsible for the quality and public health emergency activities and collaboration, community leaders, and community health workers

#### Sites and their selection

**Figure 1:**
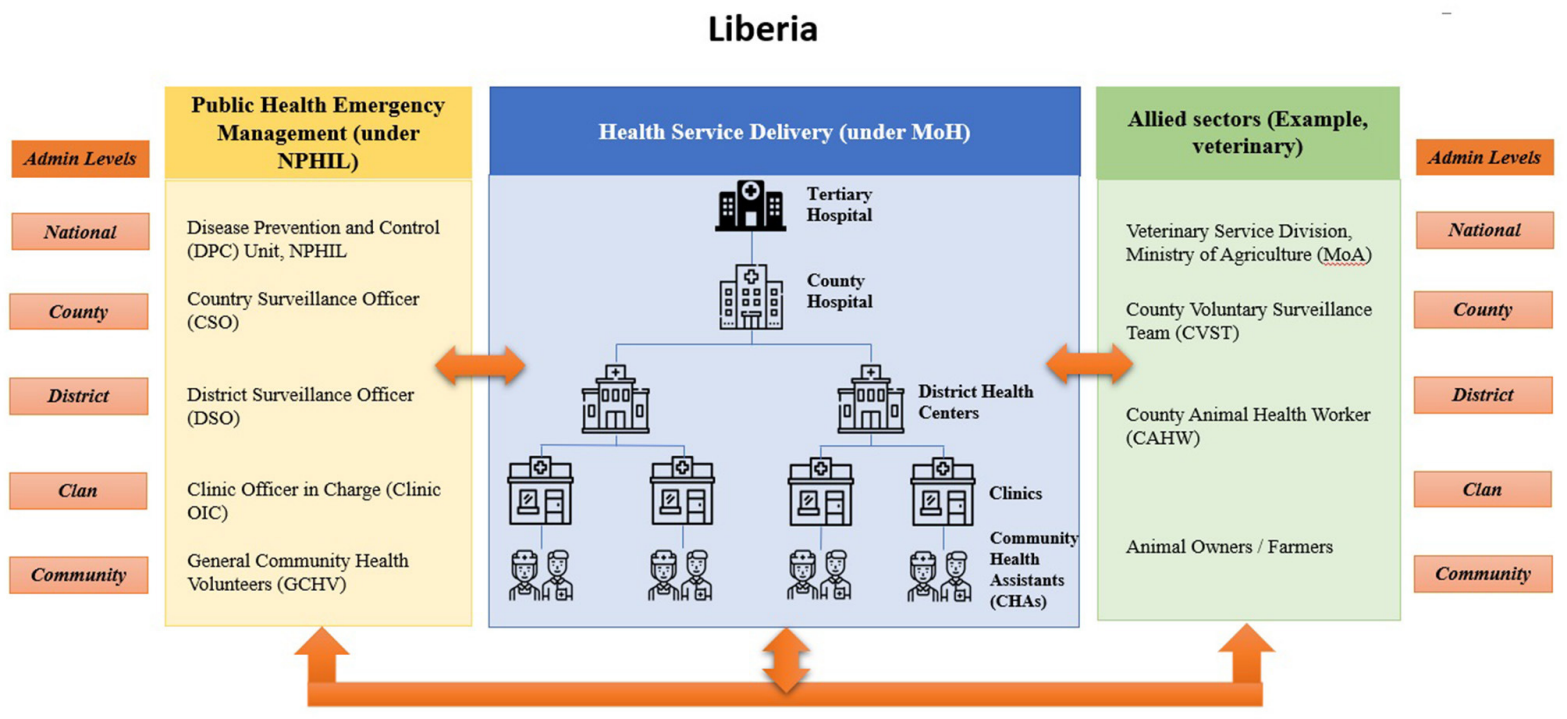
Health Service Resilience project catchment approach in Liberia.

**Figure 2:**
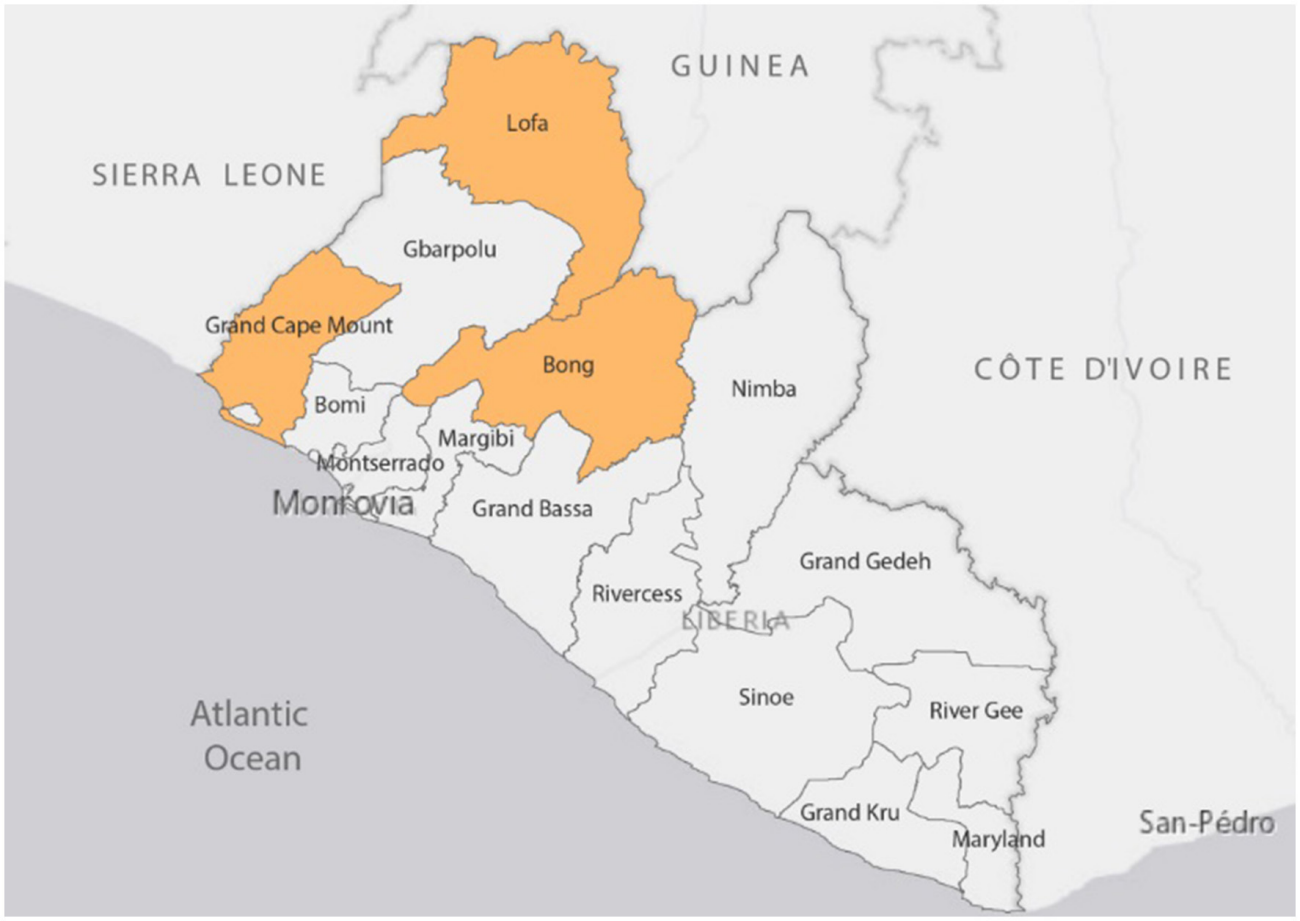
Map of Liberia showing project sites.

The selection of project sites was done through the catchment approach, which resulted in the selection of 19 health facilities (10 clinics, four health centers, and five hospitals) in three counties. Figure 1 below illustrates how this approach linked responsible entities in public health emergency management, health service delivery, and animal-environmental health across the 4 levels of the health system [national, county, district, and health facility—public and private—and community) within specified catchment areas. Implementation of the project was carried out in three counties—Bong, Lofa, and Grand Cape Mount, strategically selected in view of their geopolitical location, with significant internal and external borders (see map in Figure 2). Beyond the three pilot counties, the project activities have benefitted other counties prioritized by national authorities, for example, Montserrado, Nimba, and Gbarpolu.

#### Approach to developing and refining the project goals, tools, and activities

The project applied a mixed methods approach to assess the health system, develop project tools, implement project activities, and evaluate the level of progress. The methods included: (i) Literature review, (ii) Key Informant Interviews, and (iii) Project site assessment (county and facility visits) during the country situational assessment and project mid-term review.

- The literature review was conducted to identify existing tools to support health system resilience, for example, dedicated health system SimEx tools. Medical and health research databases (PubMed and Embase) as well as Google Scholar were searched. Search terms (e.g., health emergency, quality of care, health system resilience, etc.) were combined using Boolean Operators (AND and OR), quotation marks, and spelling variants, as applicable (17). Gray literature was identified mainly through key stakeholders' recommendations. In total, 79 documents (from both the literature search and stakeholders' recommendations) met the inclusion criteria at initial screening; 60 of them were retained for review. From the latter, 11 were peer-reviewed articles (Google Scholar-5, PubMed-4; Embase-2) and the remaining 49 documents were gray literature. Table 4 summarizes the types of documents which were reviewed.
- Key informant interviews were conducted with national (10) and sub-national (5) stakeholders (Table 3), including leadership and technical officers from relevant units within MoH and NPHIL; Environmental Protection Agency (EPA); Ministry of Agriculture (Veterinary division); WHO Country Office; Health professional training institutions; Health professional boards; Health regulatory bodies; Partner organizations; county health teams; district health teams; health facility management teams; community leaders, and healthcare workers (clinicians and community health workers). In addition, a similar group of stakeholders was consulted during an inter-country engagement meeting held in Bishoftu, Ethiopia (April 2019), and an in-country consultative workshop in Gbarnga, Bong County Liberia, in September 2019.

**Table 4:** Summary of reviewed documents for Liberia SA.

**Category**	**Reviewed**
Policies and legislations	21
Plans and strategies	16
Technical reports	24
Other technical documents (manuals, SOPs and guidelines)	18

The consultations resulted in the elaboration of the project work plan, selection of project pilot counties and facilities, validation of the SA findings, and approval of the project support package.

- Health facility and county assessments were carried out in 19 health facilities (10 clinics, five hospitals, and four health centers) in 10 health districts and 3 counties (Bong, Lofa, and Grand Cape Mount), as represented in Figure 2. Fifteen (79%) of the health facilities were public. The assessment was done using a structured questionnaire developed by the project team. The respondents included County and District Health Officers, medical directors and officers in charge, heads of units/programs, and community leaders.

#### Project support package and implementation

**Figure 3:**
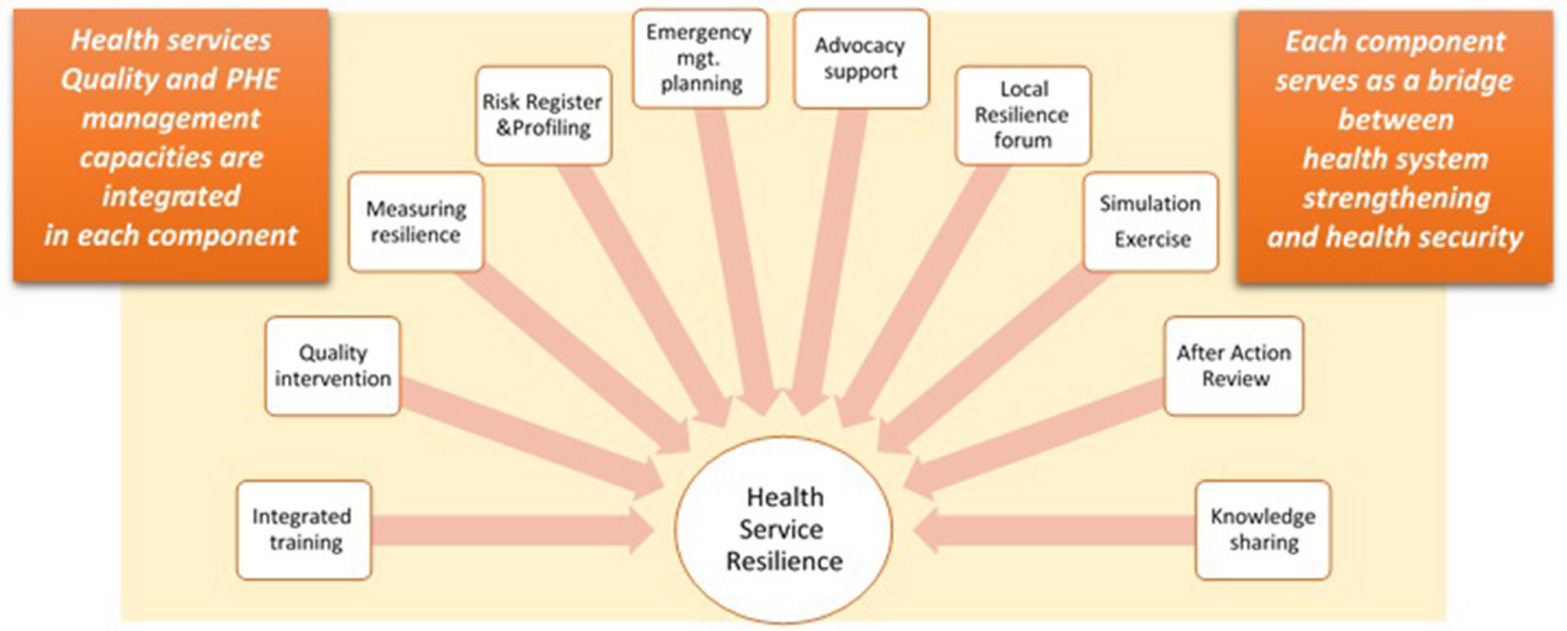
Health Service Resilience project support package.

Based on the project plan and country SA, the project team developed an operational guide for project implementation (18). The operational guide offered practical guidance on the implementation of 10 interconnected components: training and mentoring of the health workforce; health services quality interventions; measuring health system resilience; risk registering and profiling; emergency management with service continuity planning and policies; advocacy; local resilience fora; simulation exercises (SimEx); after-action reviews (AAR); and knowledge sharing (Figure 3). Each of these areas served as an opportunity to integrate emergency management capacities in routine service delivery (at primary, secondary, and tertiary levels of care) and overall strengthening of all health system building blocks (18, 19).

#### Monitoring and evaluation

Routine monitoring and evaluation (M&E) of the project uses a framework of five expected outcomes, 15 outputs with specified activities, and the status of implementation.

The M&E mechanism for the project involves all stakeholders at both national and global levels. It includes monthly reports to the national and WHO country office leadership and technical teams; quarterly meetings to provide updates to the KOICA regional team and review progress and challenges encountered; and bi-annual teleconferences with the KOICA-GDEF team, during which WHO provides an update on activities implemented and next steps.

In addition, a project mid-term review (MTR) was conducted in Monrovia in September 2021 involving national and sub-national institutions and stakeholders (see Table 3), with support from the WHO team and participation of KOICA Nigeria country office and other partners (20). The MTR assessed the status of project implementation and identified challenges and opportunities for continuity and scale-up. Data were collected through (1) a field assessment tool exploring the implementation of activities in the 10 technical support areas of the project, including versions for national, county, district, and health facility levels, (2) an online survey administered before the MTR meeting to all participants invited to it (26 out of 36 invited participants completed it), and (3) three focus group discussions (FDGs) (three groups of 12 participants each). The survey and FDGs aimed at gathering a more nuanced and in-depth understanding of the contributions of the HSR project to enhancing collaboration, integration, and health service resilience. Data from the field assessment, online survey, and focus group discussion are reported in the Section Overall evaluation by stakeholders.

### Project achievements and contributions to long-term recovery and building back better agenda in Liberia

The HSR project supported the MoH and NPHIL to strengthen Liberia's health system to deliver quality routine health services before, during, and after the COVID-19 pandemic. During the COVID-19 outbreak, project activities were re-aligned and adapted to meet the pandemic-driven priorities of the health system. The HSR project contributed to policymaking, strategic planning, surveillance, measurement of quality and resilience, human resource planning, and capacity building (in-service and pre-service) according to the 5 project outcomes.

#### Overall evaluation by stakeholders

**Figure 4:**
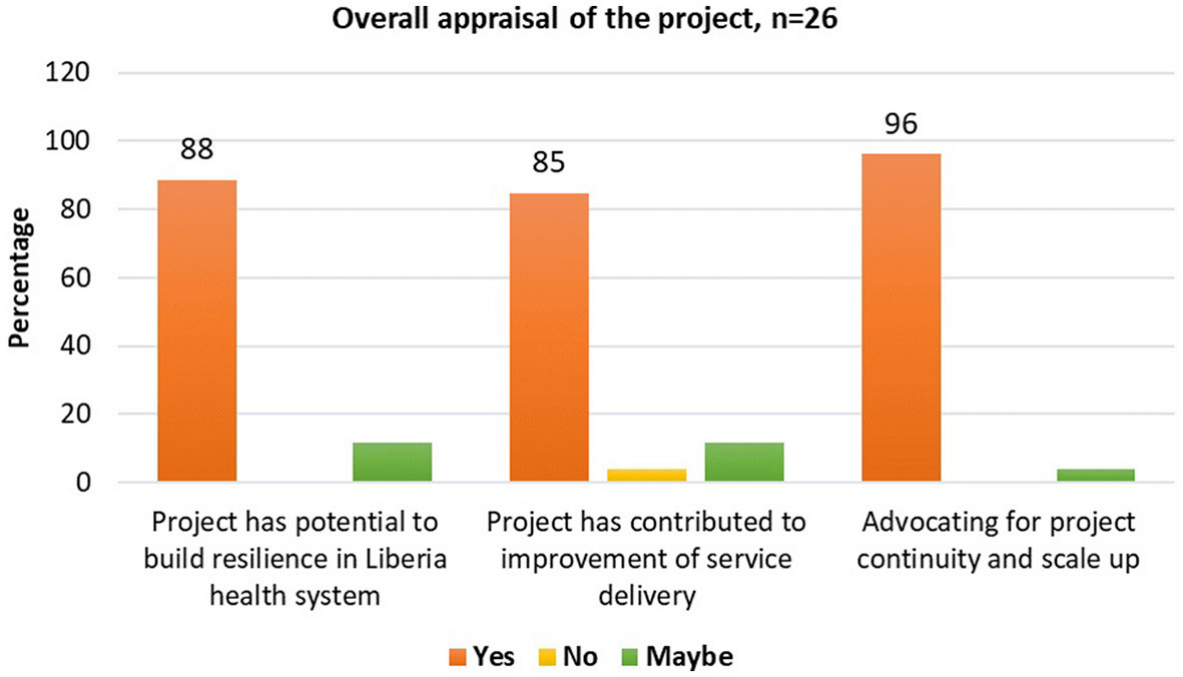
Stakeholders' appraisal of the Health Service Resilience project in Liberia.

From stakeholders' consultations conducted as part of the project's mid-term review, most key stakeholders (*n* = 26 respondents) at national and sub-national levels agreed that the project has: (1) contributed to health service improvement (85%), (2) has the potential to strengthen resilience in the health system of Liberia (88%), and therefore (3) advocated for project continuity and scale-up (96%) (see Figure 4). The project approach was appreciated particularly for its uniqueness in promoting the integration of quality health services and emergency preparedness and response (85%), promoting inter-sectoral and multi-disciplinary collaboration (81%), and encouraging ownership and sustainability of Quality improvement interventions (QI) carried out (85%).

The quotes below are the feedback received from the administrator of Kolahun Hospital, Lofa County, and the IPC focal person for Grand Cape Mount County concerning their impression of the Integrated approach used by the KOICA-funded project during health sector operational planning in Lofa and Grand Cape Mount counties.

“*This integrated approach of supervising clinicians with administrators, cleaners and security officers in hospitals is very unique and need to be encouraged. It gives us the opportunity to understand challenges in service delivery and how we can jointly find feasible solutions”*.

***Mr John Kawala*** *, Administrator Kolahun District hospital, Lofa county*

“*This approach of jointly reviewing the health system and planning together with colleagues of the animal, agriculture and environmental sectors is the first of its kind. The discussions were very rich & comprehensive due to the diversity of the group and the application of the novel Health System SimEx tool developed by the KOICA project”*.

***Victoria Railey*** *, IPC Focal Point, Grand Cape Mount county*

“*The project has ensured participation of various stakeholders. Activities are conducted through the MoH and NPHIL making the entities to assume ownership and for long-term sustainability”*

***Mr Garrison Kerwillain*** *, IPC coordinator, MoH*

#### Improvements in key resilience indicators

Table 5 below summarizes progress made in key Health System Resilience indicators (developed by the HSR project) between 2019 (project start year) and December 2022, across the building blocks and levels of the health system. This progress also includes some of the weak areas identified during the Liberia SA on the state of quality and resilience. Overall, the project has contributed to the advancement of key resilience indicators in Liberia.

**Table 5:** Progress in the attainment of key HSR indicators in Liberia (especially project counties).

**Level of health system**	**HSR indicator**	**HS building block**	**Baseline (2019)**	**Current (2022)**
All	Availability of a protocol for prioritization of services to be maintained during emergencies	Leadership	No	Yes
County	Proportion of counties that have conducted/updated vulnerability and risk analysis and mapping	Leadership	0%	100%
National	Availability of a designated authority for health service/system resilience functions	Leadership	No	Yes
National	Availability of a designated health system focal person or team responsible for providing input in the SPAR C9 assessment process	Leadership	No	Yes
National	SPAR health service provision capacity (C9) score	Leadership	33%	Level 3^*^ (50–75%)
All	Availability of a platform to share good practices and lessons learned from healthcare facility perspectives in the context of emergencies	Leadership	No	Yes (Monthly QoC TC)
Facility	Availability of clinical protocols for priority public health emergency case management	Service delivery	Yes	Yes (COVID-19)
County	Simulation exercise conducted in the last 12 months that includes testing operational capacity at the county level for EPR	Service delivery	No	Yes
Facility	Number of health facilities in Liberia reporting adverse events in care delivery	Service delivery	0	2
All	Availability of an all-hazard EPR plan (or equivalent) which includes planning for the continuity of routine health services in the event of PHE	Leadership	No	Yes
Facility	Percentage of facilities in project sites with a dedicated IPC focal person	Service delivery	53%	100%
Facility	System in place to assess community trust (patient-reported experiences/outcomes)	Community engagement	74%	100%
Facility	Percentage of facilities in project sites with a designated team or focal persons for emergency management and service continuity	Workforce	37%	100%
Facility	Percentage of facilities in project counties with personnel that have received training with a focus on Health Service Resilience	Workforce	63%	100%
All	Monitoring and evaluation mechanism established for measuring the resilience of health services to PHE as part of routine health information system	HIS	95%	100%
National	Availability of HS SimEx package to test HS capacity for EPR and MEHS	Leadership	No	Yes
All	Availability of functional Quality Management Teams with ToR	Leadership	No	Yes

^*^ Liberia SPAR 2022.

#### Progress in project implementation

**Figure 5:**
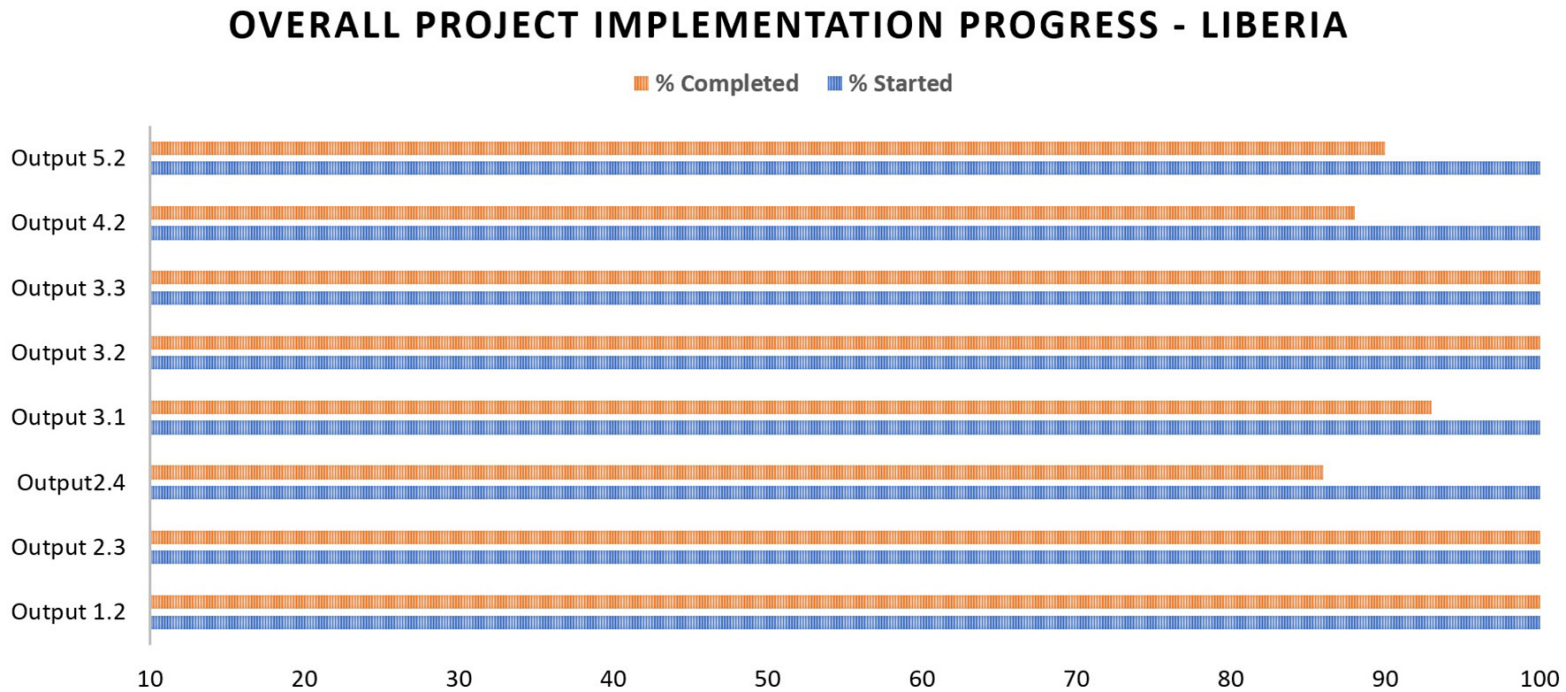
Implementation status of the Health Service Resilience project in Liberia.

As of December 2022, 95% of the project activities were estimated as completed (see Figure 5). The delay in achieving 100% completion has resulted from: delays in commencing implementation due to administrative and operational bottlenecks; disruptions and re-prioritization of project interventions due to the COVID-19 outbreak; and the longer than predicted time required to develop, adapt, and roll out the project support package at sub-national level. Despite the above delays, the project implementation would be completed within the set timeline of October 2023. Pending interventions are related to the roll out of key project tools such as the HSR training package and the Health System SimEx package.

#### Achievements relative to Liberia Investment Plan for Resilience

The project has also contributed to the implementation of the Liberia Investment Plan for Resilience, especially in building resilient capacities that enhanced preparedness, response, and recovery from the COVID-19 outbreak. For example, the project supported NPHIL and MoH to conduct a Vulnerability Risk Assessment and Mapping (VRAM) in 2021 (in line with the “awareness” feature of a resilient health system); implement a package of activities with a focus on primary healthcare (“diversity” feature of a resilient health system); enhance surge capacity by supporting integrated training for health workers to ensure rapid detection of outbreaks and minimize disruption of essential services (“self-regulation”); strengthen joint working between health systems and health security (“integration”) and develop tools for testing health system functionality (e.g., Health System SimEx); and planning for health service continuity during public health events (“transformation”).

**Figure 6:**
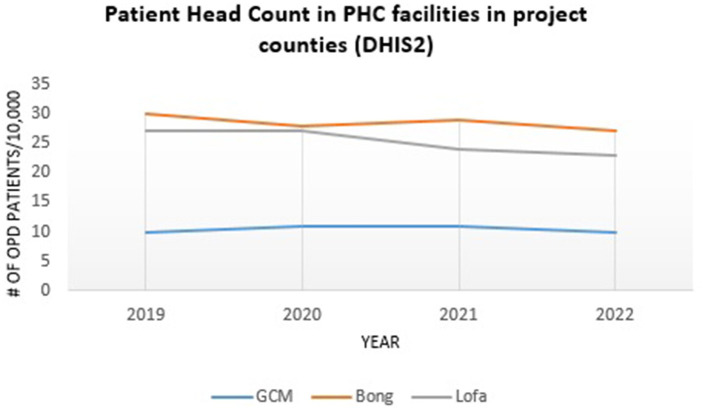
Primary healthcare head counts in project counties during COVID-19 pandemic.

Further research is needed to explore the relative contribution of this project to resilience outcomes as opposed to processes and structures, but there are some indicative data in this respect. For example, outpatient consultations in public and private primary healthcare facilities increased from 3.4 million (2020) to 3.6 million (2021) across the country (16), with a similar trend observed in two of the project counties (Bong and Grand Cape Mount) as depicted in Figure 6.

Table 6 summarizes the achievements of the project across the package of support components, the Liberia Investment Plan for Building a Resilient Health System, and the strategic areas for improving health system resilience during COVID-19 (22).

**Table 6:** Summary of HSR project achievements aligned with the health system building blocks.

**Examples of achievements by components of the project's package of support—cutting across all health system building blocks**	**Liberia investment plan priority mainly contributed to/HS resilience assessment framework (strategic areas)**
1. Advocacy—Governance • Continuous advocacy at all levels national and sub-national has led to ° High-level political buy-in and commitment to project implementation and institutionalization of the integrated health system strengthening approach promoted by the project as key for making health systems resilient, especially during the COVID-19 outbreak, e.g., participation of the Minister of Health and DG for NPHIL during project consultative meeting and national workshops to validate the Health System SimEx package and set of resilience indicators. ° strengthened joint working between stakeholders focusing on health security and those focusing on health system strengthening as well as those from service delivery levels (public and private) and allied sectors, e.g., animal, environmental health, and education. Example: joint planning for emergency preparedness and response and joint simulation exercises to test the resilience of the system.	•Expand capacity for leadership and governance to ensure effective guidance of health actions (Strategy 1–9)
2. Integrated training—Human Resource for Health Increased health workforce competency in building health system resilience through an integrated approach with awareness of their roles at leadership and service delivery levels. For example, through • Developing and making available (for in-person and online training) a training course on an integrated approach to health system resilience with a focus on services (21). • Developing and launching the Continuous Professional Development (CPD) guideline and program (including integration of the HSR training course) for in-service training of health workers in the country • Integrated training for over 1,000 health workers, including policymakers, managers, and health service providers (including those at primary care levels and port health officers, at national and sub-national levels) to build capacities in various technical areas required for health system strengthening and resilience, e.g., application of health system resilience concepts, health services quality including safety requirements for building resilience [e.g., infection Prevention and Control (IPC) and Water Sanitation and Hygiene (WASH), Antimicrobial Resistance, and healthcare-associated infection (HAI) surveillance], detection and management of priority public health threats like COVID-19 and ensuring continuity of quality routine health services in routine and emergency contexts. • Incorporation of the HSR concept in pre-service education curriculum for long-term capacity building among physicians, nurses, and midwives. For example, contributed to the reforming of the A.M Dogliotti Medical training curriculum of the University of Liberia, in collaboration with Yale University.	•Build a fit-for-purpose productive and motivated health workforce that equitably and optimally delivers quality services • Strengthen epidemic preparedness, and surveillance and response (Strategy 13, 14, 15)
3. Risk profiling • Contribution to and facilitating capacity building for vulnerability and risk profiling, e.g., supporting the development of the VRAM data collection tool • Updated risk profiled with awareness of key stakeholders—not only emergency teams	•Strengthen epidemic preparedness, and surveillance and response (Strategy 3)
4. Policies, strategies, and plans for emergency management with routing health services continuity—Governance • Informing and supporting national policies, planning, regulations, and other strategic actions to prioritize, enable, and mainstream resilience-focused activities adapted from the project's package of support and technical tools. Examples include, ° Adaptation of the projects' handbook to develop a National Guideline for Health Services Continuity Planning against the disruptive impact of public health emergencies in Liberia. ° Incorporation of integrated HSR considerations in the development of key documents such as the emergency preparedness and response plans, new Essential Package of Health Services (EPHS II), National Health Policy and Plan (NHPP) 2022-31, Health Sector Strategy and National Health Quality Strategy (NHQS) 2022-26, and facilitation of the development of the national health financing strategy and the consolidation of the National Health Accounts for FY2019 • Development of health facility accreditation standards in collaboration with Liberia Medical and Dental Council (LMDC) with an increased focus on the quality of health services and emergency preparedness as requirements for resilience-building	•Expand capacity for leadership and governance to ensure effective guidance of health actions • Establish sustainable health financing systems that will ensure efficiency and equity in the use of health resources (Strategy 16, 17, 18, 19, 20)
5. Quality (including safety) health services—Service Delivery • Strengthening health services delivery with quality improvement measures in routine and emergency contexts example through the ° Reactivation and strengthening of quality management teams (QMTs) to oversee quality improvement interventions in 11 county public hospitals, with a focus on building resilience ° Prioritization of routine health services continuity as an incident management system (IMS) pillar in coordination with teams responsible for service delivery, e.g., in the COVID-19 response. ° Incorporating considerations for routine health services continuity in the development of national and county-level outbreak preparedness and response plans ° Facilitating the participation of service providers at various service delivery levels, including those at primary care levels to actively participate in emergency preparedness and response including planning, simulation exercises, trainings, etc. ° Provision of ICT equipment and laboratory reagents to seven referral hospitals to enhance AMR surveillance and Antimicrobial Stewardship (AMS) activities in collaboration with WHO-AFRO, including the development of the AMS guideline.	•Restore and enhance service delivery systems to ensure the quality of care for patients and safe working environment for health staff • Strengthen epidemic preparedness, surveillance, and response • Build adequate capacity for the management of essential medicines and supplies at all levels (Strategy 19, 20)
6. Measuring health services and system resilience—Health Information System • Development of a set of indicators for measuring health services quality and resilience for adaptation and application at country levels in alignment with existing measurement tools, including SPAR C9 • Application of selected indicators in need assessments to understand the level of competency among healthcare workers in health services quality and system resilience • Assessment of health facilities' performance in quality of health services to identify and address gaps and build capacity for improvement ° Example: assessment of 40 healthcare facilities assessed for IPC and WASH practices using the WHO IPC Score Card and WASH-FIT tool, assessment of the status of implementation of maternal and new-born quality of care standards in 12 healthcare facilities, and utilization of the Harmonized Health Facility Assessment (HHFA) tool for health facility surveys on areas of quality and resilience • Establishment of the baseline data on antimicrobial consumption and use in seven referral hospitals through adaptation and application of the WHO Point Prevalence Survey (PPS) • Facilitated the national case management pillar to conduct an assessment of county capacity for EVD case isolation and treatment in five counties	•Strengthen the health information, research, and communication systems • Restore and enhance service delivery systems to ensure quality of care for patients and safe working environment for health staff (Strategy 20)
7. Testing the resilience of services and the system through simulation exercises—Health Information System • Adaptation and application of the HSR SimEx package (using a multi-sectoral approach—Human and public health, Agriculture, and Environmental sectors) to test and review health system functionality and resilience to proactively identify and address gaps; for example, in reviewing the functional capacity in six counties for emergency preparedness and response and routine service provision, and in reviewing of the functionality of Rapid Response Teams for COVID-19 in 15 counties • 150 multidisciplinary stakeholders in six counties trained in the application of the health system resilience SimEx package • Utilization of HSR simulation exercises results to guide six County-level health sector operational planning (Bong, Lofa, Grand Cape Mount, Nimba, Bomi, and Gbarpolu)	•Strengthen the health information, research, and communication systems (Strategy 3)
8. After and Intra Action Reviews from a health system perspective—Health Information System • Review of traditional approaches after action reviews which identified gaps and provided recommendations for the application of more integrated, system-focused approaches in conducting intra and after-action reviews • Application of health system perspectives in reviewing health system performance in the context of real public health events to enable learning and improvements as key aspects of resilience example intra-action reviews of COVID-19 response in three Counties, which informed the national Transition Plan	•Strengthen the health information, research, and communication systems (Strategy 4)
9. Local resilience forum—Governance • Establishment of multi-sectoral and multi-disciplinary working groups on resilience with the integration of health services quality and health security aspects. For example, the project has promoted the One Health Platform • ***Establishment of working groups** on resilience with the integration of quality and health security aspects, e.g., Quality Management Teams, Teleconference on quality*, and instituted a monthly teleconference that brings stakeholders from all levels of the health system to discuss issues around quality of care and resilience	•Restore and enhance service delivery systems to ensure quality of care for patients and safe working environment for health staff • Strengthen epidemic preparedness, and surveillance and response (Strategy 1, 8)
10. Knowledge sharing—Health Information System • Enabling institutionalized knowledge and experience sharing as a means of building health system resilience capacities, for example, establishing and sustaining a quality-of-care virtual platform (QoC Teleconference) for knowledge sharing and learning within and between cadres and administrative levels from 15 counties to promote quality improvement activities as a requirement for resilience • Publishing project case example to contribute to evidence and global learning on operationalizing health system resilience at the country level, e.g., as part of WHO Health System Resilience Toolkit, and Health Services Learning Hub and Action Brief to share best practices in quality improvement during the COVID-19 pandemic response	•Strengthen the health information, research, and communication systems (Strategy 4, 7)

#### Achievements in mobilizing and supporting the health workforce

The project contributed to the mapping of technical competencies among healthcare workers. It also supported integrated training in COVID-19 prevention and control for over 1,000 health workers (clinicians, health system managers, surveillance officers, administrators, environmental and veterinary officers, and community health workers) in Bong, Lofa, and Grand Cape Mount counties. Key elements of this training addressed Water, Sanitation, and Hygiene (WASH), case management, and the application of maternal and newborn quality of care standards in the context of an infectious disease pandemic. These efforts contributed to zero COVID-19-related death and zero maternal mortality in Grand Cape Mount County between March 2020 and September 2021 (20).

The project has supported long-term improvement in workforce capacity by contributing to the establishment of an integrated learning platform on healthcare quality and a program for Continuing Professional Development (CPD).

#### Achievements in strengthening public health capacities

Having learned from the EVD outbreak and other emergencies, the project tailored emergency preparedness and response activities to also support and allow the continuity of essential health services during emergencies. For example, following advice from the project team and in coordination with responsible stakeholders, the monitoring and maintenance of essential routine health services were incorporated as a response priority of the COVID-19 Incident Management System. Adopting a catchment approach (such as during stakeholders' workshops) facilitated the cross-pollination of knowledge and skills across the human, animal, and environmental sectors. Involving health facilities at the various levels across the referral system (clinics, health centers, and hospitals), the strengthening of the roles of actors at each level (2), and the creation of interlinkages between and within the different levels and sectors strengthened resilient capacities at service delivery levels to create sustainable impact and foster efficiency.

The HSR project catalyzed Liberia's efforts of building back better from EVD, COVID-19, and beyond, by embedding public health capacities in health system functions based on lessons from past and current public health events. Examples include the prioritization of routine health services continuity and the inclusion of a new response pillar called Maintenance of Essential Health Services (MEHS) during the COVID-19 pandemic (23). This has supported the strengthening of routine health services during COVID-19 as well as the roll out of COVID-19 vaccination, integration of clinical care for COVID-19 patients into routine healthcare, and revision of the essential package of health services (EPHS II, 2022–2026) to include concepts of emergency response and healthcare quality improvement.

In addition, the concept of sustainability has been embodied in the implementation of the project by leveraging and supporting existing partners' efforts to strengthen the health system in Liberia. The project team also facilitated the development of the new National Health Quality Strategy (NHQS 2023–2027) and is supporting the Liberian Medical and Dental Council (LMDC) to draft quality standards for the assessment and accreditation of health facilities in Liberia. These documents will guide the provision of quality healthcare services in the post-COVID-19 era.

#### Project contributions to the COVID-19 response

The ability to quickly reallocate available funding to support the COVID-19 response was necessary to provide a swift response (24). At the peak of the pandemic, some project activities were revised to re-align with COVID-19 response activities, and project funding was rapidly repurposed to address these needs. Such activities included integrated training in COVID-19 prevention/IPC, detection, isolation, and case management (including home-based care and specific interventions for maternal, newborn, and child health services) in the three project counties. Funding from the project was also used to support surge capacity through the repurposing of project staff to support COVID-19 response in non-project counties.

Technical officers for the project were repurposed to support the COVID-19 response in Montserrado and Grand Kru counties. This support resulted in the development of specific county response plans for COVID and the successful containment of the outbreak. The HSR project personnel contributed to the adaptation, validation, and roll out of the interim guidelines for COVID-19 clinical care and the Handbook for COVID-19 Treatment, which facilitated the management of COVID-19 patients in Treatment Units and county referral hospitals. The project focal point also facilitated the development of the national guideline for home-based isolation and care for COVID-19 patients, which facilitated the treatment and recovery of over 2,000 patients during the response (25).

In addition, through the HSR project, WHO procured and donated laptop computers and printers to eight referral hospitals to improve data collection and reporting on AMR during the COVID-19 outbreak and beyond. The water supply at Tellewoyan Memorial Hospital (Lofa County) was rehabilitated through the procurement and installation of a submersible water pump under the HSR project. This enhanced the quality of care for COVID-19 case management and other patients in routine care for the population of Lofa and the neighboring communities in Guinea and Sierra Leone.

### Project challenges and limitations

Early on, there was a 6-month delay in the onset of project implementation due to administrative and operational challenges such as delay in the recruitment of project staff and transfer of project funds to the country office. In addition, project implementation was disrupted by the COVID-19 pandemic response and restrictions, and competing priorities related to ongoing and potential outbreaks, including threats of EVD. However, the lost time was recovered by careful work planning, re-sequencing, and streamlining of activities in line with national and sub-national priorities while maintaining the project's ethos of fostering an integrated approach to health system strengthening for resilience even while adapting activities to support response to various emergencies.

A key limitation of the project was its relatively limited scope. The project was implemented in 19 public and private health facilities in three out of 15 counties in Liberia. However, there were spillovers of project activities in non-pilot counties, such as Montserrado, Nimba, Bomi, and Gbarpolu. Some activities were also implemented at a national level, benefitting stakeholders from MoH, NPHIL, MoA, EPA, National Disaster Management Agency (NDMA), and academia, and are likely to have had an impact on other activities involving the same stakeholders. Importantly, the health system challenges are similar in all 15 counties of Liberia, which will facilitate the transfer of lessons learned from the 3^+^ pilot counties across the health system.

## Discussion and recommendations

To support Liberia in building resilience in its health system, WHO partnered with KOICA to work with the MoH, NPHIL, and other relevant institutions to implement a health system resilience initiative. The project, though catalytic in nature and limited in scope, made a significant contribution toward the realization of the national ambition of building a resilient health system, especially in the three project counties.

Below we highlight what we perceive as the greatest strengths and persistent challenges for the Health Service Resilience project as implemented in Liberia and, based on these, recommend priorities in working toward health systems resilience. Highlights of the achievements of the parallel project in Ethiopia are included in Box 2.

Box 2 **Highlights of project implementation in Ethiopia.**

This resilience building project model is also being successfully implemented in Ethiopia with positive feedback from stakeholders acknowledging its role in promoting and operationalizing the concept of resilience in the health system as important in the context of recovery from various public health events. This demonstrates the relevance, adaptability, and applicability of the project to different contexts.

Examples of achievements from project support in Ethiopia include establishment of an institutional focus on health system resilience in the Ethiopian Public Health Institute (EPHI) and institutionalizing resilience-focused activities from the project in national public health activities as planning, Public Health Emergency Management (PHEM) guidelines. Emergency response efforts such as the COVID-19 incident management structure incorporated essential health service continuity as a priority, with active participation of health system and services-focused teams for a comprehensive response and timely prevention and mitigation of routine health services disruptions. National universities in Ethiopia have also embedded the concept and application of health systems resilience in their pre-service and advanced degree programs for health professionals, based on the training package from this initiative for long-term impact in health workforce competencies. These are especially timely to further position and expand considerations for integrated health system strengthening and resilience in recovery efforts from COVID-19, conflict-related humanitarian response and other system strengthening initiatives building on the projects best practices and achievements.

### Health systems strengthening for resilience needs an integrated approach and multi-partner, multi-sectoral collaboration

Fragmented and siloed approaches have often hampered efforts to make health systems more resilient (26), by prioritizing disease-specific objectives over health system strengthening and clinical care over public health and by not making the most of limited resources. The HSR project, in contrast, mainstreamed the joint working of key stakeholders across various disciplines and sectors at policy, planning, and operational, including service delivery, levels.

The project strengthened the integration of health systems and health security work. It ensured that each project-supported activity contributed to strengthening the capacity of the health system to equally prioritize and serve routine and emergency-related population health needs during and between small- and large-scale emergencies. High-level political buy-in and participation from the top leadership of the MoH and NPHIL was secured. This included the participation of the Minister of Health and Director General of NPHIL during the project stakeholders' consultative meeting in Gbarnga and during the launch of the project toolkit (as shown in Table 6).

The principle of integration also guided the development of national and county health sector and health security plans and policy documents, as well as technical resources developed or supported by the project. Key among these was the integrated Health Sector Operational Plans that included emergency preparedness activities, the integrated health system SimEx package (which was used to test the functionality of the Rapid Response Teams for COVID-19), COVID-19 Preparedness and Response Plans (23) (national and county), and the national guideline for health service continuity planning in emergency context (27) (further detail in Table 6). Health system reporting and programming documents, such as the State Party Self-Assessment Annual Reporting (SPAR) and the antimicrobial resistance (AMR) stewardship programming, were also developed using the integrated approach.

The project achieved significant successes in institutionalizing joint working within and between the health sector and other sectors, such as education, academia, administration, security, internal affairs, agriculture, animal and environmental health, and civil society. Among the most successful examples of such institutionalized joint working and multi-sectoral collaboration were the project activities on public health emergency preparedness; the promotion of the One-Health platform to improve coordination with the animal and environmental sectors; and the national workshop to review and adapt the Health System SimEx package and set of indicators for resilience (Tables 5, 6). The collaboration between the private and public sectors was also strengthened, with the critical role of the private sector becoming evident during the COVID-19 pandemic.

Last, but not least, as far as integration and collaboration are concerned, the project achieved a high level of partner engagement, benefiting from the technical knowledge and skills of partners and driving the agenda for sustainability. As shown in Table 6, the project team has collaborated with Last Mile Health to develop the NHQS2023-27, USAID-STAIP project to conduct integrated training for healthcare workers and conduct health sector operational planning at the county level and the USAID-funded BRIDGE-U project to develop and launch the CPD guideline.

Liberia's successes in improving integration and multi-partner, multi-sectoral collaboration for resilience offer a broad range of steps for other countries to choose from. Within the country, there is a need to further institutionalize integrated health system resilience efforts, including linkages with funding streams and accountability mechanisms (28). The Ministry of Health and NPHIL need to work together to steer available and potential internal and external health investments to promote and operationalize an integrated approach to health system strengthening toward resilience, even when targeting specific health problems such as emergencies, specific diseases, or life-course issues. This would build the capacity of the system to effectively address the various health issues faced by populations served with better efficiency and sustainability.

### Health system resilience requires continuous learning and capacity building

The project's package of support (18) remains relevant and timely before, during, and between public health emergencies, thereby allowing continuous learning and improvement even as the health system goes from one public health event to the next. This continuous learning is core to the concept of recovery and long-term resilience building (2) promoted by the project. It contributes to making the health system more adaptive as illustrated by Falope et al. (29). Building on lessons from EVD, COVID-19, and other smaller events, the project established the multi-sector/multi-disciplinary learning platform for regular experience sharing on healthcare quality and resilience between health workers, managers, policymakers, community stakeholders, and partners (Table 6, rows 2 and 10). It also developed and launched the first national guideline for CPD, in collaboration with the University of Liberia and health professional boards (Table 6). Enhancing the competencies of medical directors, CHOs, and other managers and decision-makers through project activities has also enabled a trickle-down effect on frontline health professionals and non-health actors (at both national and sub-national levels) and has enhanced the application of systems thinking for resilience at frontline levels.

Despite the frequent use of the term “health systems resilience”, the understanding of the concept of integrating resilience building in routine health emergency and system functions and service delivery, particularly at sub-national levels and service delivery levels, is generally still growing (30). There is a need to cover more health professionals (especially from the frontline) in training and orientation on the required integrated approach to the concept of resilience, e.g., through more pre-service curricula reviews and CPD programs beyond the current project sites.

### Health system resilience requires the promotion of ownership and sustainability

The project has been intentional and systematic in creating a sense of ownership among national stakeholders and in enabling long-term sustainability. As described under *Guiding principles*, it used a “One-plan One-budget approach” from planning through implementation. Its overall work plan was jointly elaborated by national stakeholders from the MoH, NPHIL, EPA, and MoA during an inception meeting held in Bishoftu Ethiopia, and a stakeholders' consultation meeting in Gbarnga, Bong County in Liberia in 2019. Budgets for the implementation of activities were jointly reviewed and updated with the national counterparts, particularly the two project focal points, and approved by the Minister of Health or Director General of NPHIL before each activity was implemented. In addition, the planning and coordination of some key activities have been handed over to the national teams. For example, the Healthcare Quality Management Unit (HQMU) of the MoH leads the coordination of monthly teleconferences on quality of care (29) (Table 6).

Health system resilience efforts must be further decentralized (28) and owned by key actors within the system, especially at the sub-national levels with a focus on strengthening primary healthcare (22, 28, 31).

### Health system resilience requires new investment mechanisms to promote research and innovation

The “panic and neglect” cycle has been a major challenge in building resilience in health systems sustainably, using the recovery phase as a platform for applying learning and improving the quality of health services (6). Most donor funding disappears when the acute phase of an emergency is over. Foundational health issues remain unaddressed and under-prioritized. After the EVD response in Liberia, the country experienced a decline in external support to take forward the lessons from the outbreak. Among the current partners working on aspects of quality and preparedness with national authorities in Liberia, there are very limited resources and support available for promoting and scaling-up an integrated health system strengthening with health security in tandem, in the context of the decline in external support and economic contraction. This is further compounded by the global economic impacts of the COVID-19 pandemic and geo-political crisis.

The occurrence of public health events (PHE) such as the COVID-19 pandemic and the threat of the Ebola outbreak, while disruptive, provide increased momentum to work toward resilience, learning, and strengthening of the health system. The HSR project is an example of a health system initiative implemented from lessons learned from previous PHE, has piloted a series of technical tools to build health system resilience, one of which is the set of resilience indicators (Tables 5, 6). However, there is an increasing need to test this package of tools on a large scale, but also to prioritize the development of robust assessment tools and monitoring and evaluation systems through research.

National and sub-national stakeholders have been calling for the extension and scale-up for the wider impact of Liberia's Health Services Resilience project. The project team and their collaborators are continuing to advocate for the expansion of the project within and beyond the three pilot counties and focused investments beyond the current project scope and the COVID-19 pandemic. One of the major investment priorities identified is addressing the fragmentation in health information systems and enabling integrated and interoperable tracking of quality and resilience indicators at both national and operational levels.

#### Challenges

Even though the project applies a system approach, there are fundamental health system challenges that have remained outside of its scope, for example, the availability of basic infrastructure for water, sanitation, and hygiene, the procurement of medical supplies, or the retention of health workers. At the same time, the lack of such infrastructure affects both the implementation of the project and the preservation of its legacy. The project's advocacy and technical support for system-wide strengthening can be enhanced by pulling together wider health sector inputs and resources and by broadening partnerships in this area of work. The project team continues to scope out relevant options for collaboration with other partners in supporting the national authorities. Sector-wide and inter-sectoral planning and linking the plans with funding are also essential.

## Study strengths and limitations

### Strengths

This study presented ongoing, practical work on the operationalization of resilience in a health system of a resource-limited setting, a genre of studies that continues to be seriously under-represented in the academic literature. The operationalization of the concept of resilience in the health system has been limited partly by the limited progress made in developing standardized metrics (32), but also by the multitude of frameworks that have been proposed (30). The pre-existing evidence synthesized here was generated through a mixed methods approach, including a literature review (quantitative and qualitative findings), online survey and focus group discussions (primarily qualitative findings), and health facility assessments (observations, giving rise to both quantitative and qualitative findings). This was complemented by considerations and learnings arising from the study authors' direct involvement in the project. The evidence and learnings presented here are thus rich and multifaceted and have sufficient rigor and credibility, even if also limited in important ways (see below). The lessons shared in this paper can be applied to enhance resilience in similar health systems recovering from COVID-19 and seeking to build back a better health system, which continuously improves how it serves the needs of local populations and its capacity to withstand future pandemics.

### Limitations

A substantial part of the data summarized in this study has been collected from key informants, selected for their roles in the health system and by applying the catchment approach. This makes such data amenable to many biases, including sampling, recall, social desirability, and overall reporting bias. This was, to a degree, offset by data triangulation using other methods, including literature reviews and observations in health facilities. Overall, a breadth of evidence was synthesized. However, this was not done by applying a sufficiently structured and transparent research synthesis method. The prioritization of findings and lessons learned was made based on the co-authors' expertise as opposed to using a systematic approach to data extraction and synthesis. Finally, the public health intervention the study describes is still in its implementation phase. Follow-up studies are needed to explore the degree to which the reported achievements and impact are lasting and sustainable.

## Conclusion

The Health Service Resilience project as implemented in Liberia has demonstrated that it is possible to operationalize health system resilience in a low-income country and under resource constraints. It has also shown that the priorities of health security and universal health coverage can be advanced in tandem at policy, management, and service delivery levels. This has been achieved by ensuring national ownership and leveraging existing strengths and opportunities such as strong political will at national and sub-national levels and the existence of NPHIL and the Quality Management Unit of the MoH, which are products of the EVD experience.

The project was mainly implemented in three out of 15 countries of Liberia, and the nature of the support has been catalytic as opposed to transformative. However, the HSR project has made a valuable contribution to national efforts for building a resilient health system inspired by the EVD experience. These achievements from the project implementation supported the national preparedness and response efforts for COVID-19 that led to the successful containment of the COVID-19 outbreak in Liberia and are contributing to the gradual restoration of essential health services in the post-COVID-19 era. The project has generated a lot of momentum and enthusiasm among the beneficiary institutions and populations, especially with its integrated, catchment, and multi-sectoral approach.

The principles applied in this pilot and the best practices shared could guide the operationalization of resilience efforts in health systems in other resource-limited settings similar to Liberia.

## Author contributions

LA-E drafted manuscript (including method), conducted desk review and stakeholders' consultations, completed editing, referencing, and submission. RS drafted outline for the manuscript, contributed to the desk review (MEF), and reviewed and refined manuscript. SS reviewed and updated manuscript. SW reviewed manuscript to reflect the context. MB contributed to desk review and stakeholders' consultation. BN supported stakeholders' consultation, especially in the identification and engagement of key stakeholders from MoH. RG supported stakeholders' consultation, especially stakeholders from the National Public Health Institute. CO and CP proofread and update the manuscript. All authors contributed to the article and approved the submitted version.
