# Supplementary material for: Liberia health system's journey to long-term recovery and resilience post-Ebola: a case study of an exemplary multi-year collaboration
Source: Front Public Health. 2023 Jun 19;11:1137865. doi: 10.3389/fpubh.2023.1137865 (PMC10317185; doi:10.3389/fpubh.2023.1137865)
Supplement: Supplementary file 1 [file Data_Sheet_1.PDF]

Making Health Services Resilient with Quality and Preparedness for Emergency Response

LIBERIA SITUATIONAL ASSESSMENT REPORT

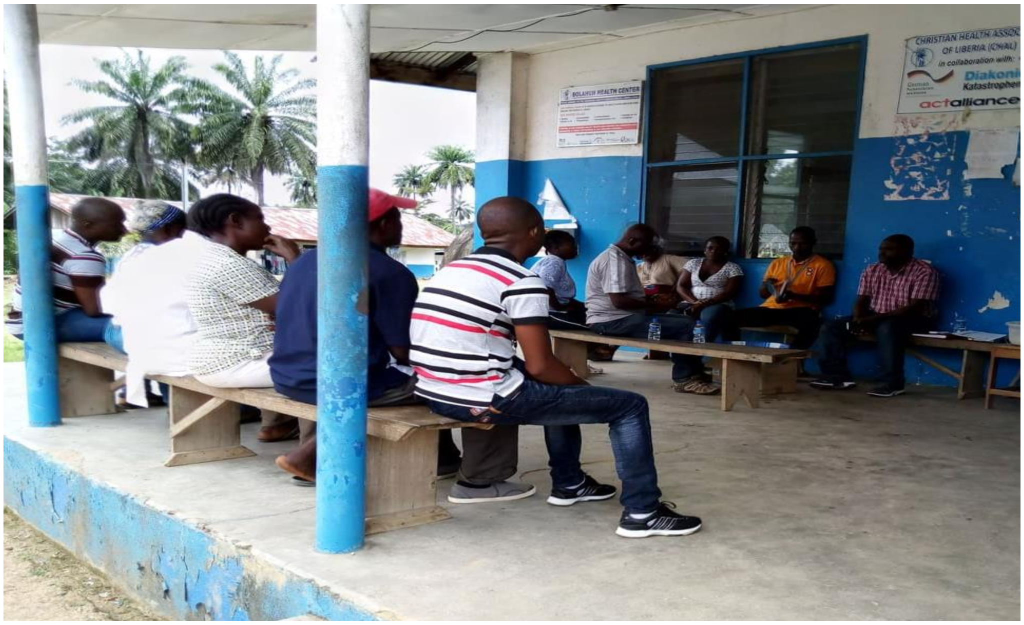

KOREA INTERNATIONAL COOPERATION AGENCY (KOICA)-FUNDED PROJECT

Table of contents

Contents

Table of contents i
List of tables iii
List of figures iv
List of photos v
Foreword vi
Acknowledgement vii
Acronyms viii
Executive Summary ix
1. Introduction and Context 1
1.1. Geography, Population and Demography of Liberia 1
1.2. Liberia Health system 1
2. Objectives of the situational assessment 3
3. Method 4
3.1. Desk review 4
3.1.1. Search strategy 5
3.2. Stakeholders' Consultations 6
3.3. Project site selection and situational assessment 10
3.3.1. Catchment and System Approach 10
3.3.2. Site Selection Matrix and Criteria 11
3.3.3. Health facility visit and assessment 13
4. Findings of the Situational Assessment 14
4.1. Review of legislations, policy and planning documents from quality and emergency preparedness perspectives . 14
4.2. Current state of implementation of relevant policies, plans and strategies on health service quality & emergency preparedness 15
4.2.1. Health service quality 15
4.2.2. Emergency preparedness 15
1.2.3. Laboratory services and emergency preparedness 16
4.2.3. Health workforce 18
4.2.4. Initiatives to strengthen health services quality and emergency preparedness 22
4.2.5. Integration of quality and preparedness in health services 22

4.3. Tools and measurement approaches in quality and emergency preparedness 23
4.3.1. Health Information System (HIS) 23
4.3.2. SARA plus Quality of Care 24
4.3.3. Measuring the outcomes of IPC Interventions Post-Ebola 26
4.3.4. Maternal and Newborn Quality of Care Standards 28
4.3.5. Joint Integrated Supportive Supervision (JISS) Quality Assurance Tool 28
4.3.6. Health Facility Accreditation 29
4.3.7. IHR Measurements for Emergency Preparedness 31
4.3.8. Performance of Veterinary Services (PVS) Gap Analysis 34
4.4. Authorities, stakeholders and their role in quality and emergency preparedness 37
5. Findings from the site assessment 39
5.1. Distribution of health facilities 39
5.2. Health worker distribution in selected health facilities 40
5.3. Leadership (policies/plans) 43
5.4. Information (measurement tools) 45
5.5. Patient and population engagement (stakeholders) 46
5.6. Regulation and standards 47
5.7. Organizational capacity in health facilities 49
5.8. Integration of quality and emergency preparedness 51
6. Levers, barriers, gaps and opportunities for integrated approach to building health service resilience 53
6.1. Levers 53
6.2. Key challenges and gaps 53
6.3. Opportunities 55
7. Limitations 57
8. Conclusion 58
9. Recommendations from the stakeholders' consultation meeting 59
9.1. On tools and measurement approaches 59
9.2. On health facility reporting on quality and emergency 59
10. Project next steps based on Stakeholders' Consultative Meeting 60
Reference 61
ANNEX 63

List of tables

Table 1: Summary of reviewed documents 5
Table 2: Inclusion and exclusion criteria 5
Table 3: Criteria and rating scale used in the appraisal of documents during the review 6
Table 4: List showing stakeholders consulted as part of situational assessments 7
Table 5: Selected health facilities for the field visit of the situational assessment 12
Table 6: Health worker distribution by county, SARA 2016 20
Table 7: Quality indicators monitored under priority areas (SARA, 2018) 25
Table 8: Summary of IPC measurement tools 26
Table 9: Selected IPC indicators for Liberia IAT 26
Table 10: Grading scale used in HF accreditation 30
Table 11: National average accreditation scores by facility types in 2013 31
Table 12: Distribution of health workers cadres by health facility and county 42

List of figures

Figure 1**:** Health Facility Density by county per 100,000 population, SARA 2018 ... 2
Figure 2: Catchment and system approach across relevant sectors and administrative levels ... 10
Figure 3: Site selection matrix ... 11
Figure 4**: Duration from sample collection to testing for IDSR priority disease by County, Liberia (January – December 2017)** ... 17
Figure 5**:** Core health worker density per 10,000 pop per county, SARA 2018 ... 19
Figure 6: JISS national baseline scores ... 29
Figure 7**:** Average health service score by clinical area ... 31
Figure 8: Distribution of health facilities assessed by type ... 39
Figure 9: Distribution of health facilities by district ... 40
Figure 10: Core workforce distribution per county ... 41
Figure 11: Proportion of facilities with a leadership structure for quality ... 43
Figure 12: Proportion of facilities with a leadership structure in emergency ... 44
Figure 13: Funding for quality and emergency at the facility level ... 45
Figure 14: Availability of reporting tools and feedback on quality and emergency ... 45
Figure 15: Data collection ledger used at the health facility level ... 46
Figure 16: Representation of health facilities per active engagement of their population in planning ... 47
Figure 17: Infrastructural and diagnostic capacity among health facilities assessed ... 48
Figure 18: Declared status of accreditation of health facilities ... 48
Figure 19: Proportion of health facilities in which personnel undergo some form of training in QI ... 49
Figure 20: Health facilities involvement in SimEx, AARs ... 50
Figure 21: Availability of strategic documents for quality and emergency at health facility level ... 51
Figure 22: Perception of an integrated model of care for resilience ... 52

List of photos

Photo 1: Consultation with LBNM 9
Photo 2: Consultation with LMDC 9
Photo 3: Engagement with TNIM trainees 9
Photo 4: Consultation with medical students and lecturers 10

Foreword

The integration of quality and emergency to build the resilience of health services for Public Health Emergency Preparedness and Response cannot be more timely than now. This approach is essential for health systems, like that of Liberia, which have been weakened by multiple public health events. Our health system was on the part of recovery from the impact of the 14-years civil crisis and the 3 years of Ebola Virus Disease (EVD) outbreak in West Africa until March 2020, when we confirmed the first case of the Coronavirus Disease (COVID-19). The ongoing pandemic has further unsurfaced the weaknesses and cracks in our health system as far as resilience is concern.

The Korean International Corporation Agency (KOICA)-funded Health Service Resilient project, implemented through the World Health Organization, is a five-year project to make the Liberian health system resilient with quality for emergency preparedness and response. As part of the foundational activities, the project team conducted a situational assessment in September 2019 in three counties (Bong, Lofa and Grand Cape Mount) to understand the existing health system architecture for quality of care, emergency preparedness and their integration therein.

Interestingly, the assessment revealed the fact that Liberia has a suitable policy environment for the integration of quality and emergency response activities to build resilience. Besides, there are existing structures (including institutions like the Health Quality Management Unit and the National Public Health Institute of Liberia), capacity building programmes (e.g. the Field Epidemiology Training Programme, FETP) and stakeholders such as various professional bodies and partners to support the integration of quality and emergency.

However, constraints in resources have impaired progress in the implementation of many public health policies and plans. The production, dissemination, roll out and compliance to various quality-related guidelines and SOPs have been compromised by limited resources. Also, infrastructure and supplies issues continue to undermine the quality of routine healthcare services provided in our facilities, including emergencies. For example, in 2018 Liberia participated in the SPAR and obtained an average C9 score of 33%, which is below the global and regional average scores.

Health system strengthening intervention like the one promoted by KOICA are very progressive and will support Liberia in the realization of its Investment Plan for Building a Resilient Health System. The recommendations and necessary next steps generated from this assessment should be taken seriously by the various stakeholders involved in the implementation of the project.

For achieve meaningful Universal Health Coverage, the Ministry of Health and partners need to invest on quality improvement of health services. With the understanding that health systems (services) and health security are two sides of the same coin, pursuing the integration of these two crucial areas in our health system is worthwhile.

Dr Francis Kateh
Chief Medical Officer, MOH Liberia

Acknowledgement

The Health Quality Management Unit (HQMU), on behalf of the Ministry of Health (MoH), is pleased with the Korean International Corporation Agency (KOICA) for funding the Health Service Resilience Project on 'Making Liberia Health Services Resilient with Quality for Emergency Preparedness and Response' which is implemented through the WHO Country Office of Liberia in three counties. This Situational Assessment has revealed the gaps in the health system of Liberia regarding quality, emergency and the integration.

The MoH is extremely grateful to Dr. Charles Ocan, Health Systems Strengthening (HSS) Advisor, Dr Louis Ako-Egbe (HSR project consultant), Moses Bolongei (National Project Lead), Zainab S. Sirleaf (National IPC Focal Person) and Quincy Goll (WASH Focal Person) at WHO Country Office Liberia for providing strategic support for the roll out of this activity in the three project counties.

Special appreciation to Dr Mesfin G. Zbelo, Acting WHO Representative to Liberia, under whose leadership this activity was conducted.

We appreciate Dr. Lekilay Tehmeh (Clinical Coordinator, HQMU/MoH), Comfort King Gbaie (Quality Assurance Officer, HQMU/MoH) and Garrison Kerwilliam (IPC Coordinator, HQMU/MoH) for their technical support to this activity.

We equally express appreciation to the County Health Teams, especially the county IPC focal persons and Clinical Supervisors, for providing guidance and technical support for the implementation of conduction of this assessment.

Dr. J. Ngormbu Ballah
Director, Health Quality Management Unit (HQMU), Liberia

Acronyms

AMR	Antimicrobial Resistance
ANC	Ante Natal Care
CEBs	Community event-based surveillance
CHA	Community Health Assistant
CHCs	Community Health Committees
CHDCs	Community Health Development Committees
CHT	County Health Team
CSO	County Surveillance Officer
DHT	District Health Team
DRT	Desk Review Team
DSO	District Surveillance Officer
EPR	Emergency Preparedness and Response
GHSA	Global Health Security Agenda
HMIS	Health Management Information System
HQMT	Hospital Quality Management Team
HQMU	Health Care Quality Management Unit
HR	Human Resource
IDSR	Integrated Disease Surveillance and Response
IHR	International Health Regulations
IPC	Infection Prevention and Control
JFK-MC	John F. Kennedy Medical Center
JISS	Joint Integrated Supportive Supervision
LMDC	Liberia Medical and Dental Council
LMHRA	Liberia Medical and Health Product Regulatory Authority
M & E	Monitoring and Evaluation
MFDP	Ministry of Finance and Development Planning
MOA	Ministry of Agriculture
MOE	Ministry of Education
MoH	Ministry of Health
NCD	Non-Communicable Diseases
NPHIL	National Public Health Institute of Liberia
OPD	Outpatient Department
PNFP	Private-not-for private
QA	Quality Assurance
QoC	Quality of Care
RRT	Rapid Response Team
SARA	Service Availability and Readiness Assessment
SWOT	Strengths, Weakness, Treats and Opportunities
UNFPA	United National Population Fund
USAID	United States Agency for International Development
US-CDC	United States Centers for Disease Control and Prevention
WHO	World Health Organization

Executive Summary

Past, recent and ongoing Public Health Emergencies around the world have highlighted the need to integrate quality of care (QoC) in to routine health care services and emergency preparedness to build resilience of the health system. For example, it was observed during the 2014-15 Ebola outbreak that poor preparedness, coupled with weakened health systems (poor quality) accounted for about 50% decline in routine health services with over 500 deaths of health worker in Liberia, Sierra Leone and Guinea. Quality has been highlighted as a key element in healthcare delivery for Universal Health Coverage. Therefore, an integrated approach to develop and sustain quality and emergency preparedness in tandem is necessary to ensure health system functionality to maintain routine and emergency related healthcare and response. The role of health facilities in the implementation of the Global Health Security Agenda (GHSA) under IHR (2005) is the focus of the Health System Resilient Project in Liberia funded by the Korea International Cooperation Agency (KOICA), through the World Health Organization. As its mission, KOICA seeks to contribute to addressing global development issues including health by pursuing global harmony and facilitating the sustainable socio-economic development of its partner countries, aiming to reduce poverty and improve the quality of life in developing countries by working towards the realization of the Sustainable Development Goals (SDGs).

The contents of the country situational assessment report reflect extensive stakeholders' consultative process that commenced in April 2019 with initial technical meetings and discussions, desk review, health facilities assessment in September 2019, stakeholders' consultative meeting which continued up to October 2019. The input from the stakeholders including regulatory bodies, academic institutions, professional organizations, and partners at the October meeting is guiding the development of package of support and indicators and the creation of blueprint for the project operationalization.

Overall, the activity was organized in two parts: a desk review of relevant legislation, policies, plans and strategies on quality of care and emergency preparedness from health services resilience perspective, and a site visit of health facilities to identify relevant stakeholders, levers, opportunities and barriers for integration of quality and emergency at health facility level. The joint health facility assessment further provided better understanding on the state of quality and emergency preparedness in the health system and further inform the implementation of this project. The assessment was conducted by a multi-disciplinary team from the Ministry of Health (MOH), National Public Health Institute (NPHIL) and WHO Country Office (WCO) in collaboration with country and district health teams using an assessment tool that was co-developed by the team.

A total of 19 health facilities (comprising 10 clinics, 4 health centers and 5 hospitals) were selected from 3 counties and 10 health districts using a catchment approach. The situational assessment revealed the presence of national policies, regulations or legislations policies to support initiatives in building health services resilience by improving quality improvement and emergency preparedness. However, health services quality and preparedness were mostly considered in silo. Among the 79 documents reviewed, 36 were policies/regulations/legislations and plans. From the plans and policy documents reviewed; 35 (97%) highlighted quality of care (QoC) in isolation, 24 (67%) focused on emergency preparedness and response only, while 12 (33%) mentioned an integrated approach to QoC and emergency preparedness.

A deeper dive into the current state of implementation of these policies and plans found that 18 health facility initiatives based on relevance to the subject exist. From these, 10 are health service quality-related initiatives (5 are ongoing), while 8 are emergency-related initiatives (6 are ongoing). The 2015-2021 Investment Plan for Building a Resilient Health System is a key policy document which makes explicit mention of integration of quality and emergency preparedness. There is a national drive for integration, however, this integrated approach is yet to be well-developed and embedded within health system and health security initiatives aiming to strengthen health services.

The assessment also identified many measurement approaches and tools concerned with quality in health services (e.g. HMIS, SARA plus QoC, IPC assessments, WASH-FIT, Maternal and Newborn Quality of Care Standards etc) as well as health security. Most of the QoC measurement approaches are EVD-based and mainly on IPC. International Health Regulation (IHR) measurements for emergency preparedness include; State Party Self-Assessment Annual Reporting (SPAR), Joint External Evaluation (JEE), Simulation exercise (SimEx), After Action Review (AAR), and Performance of Veterinary Services (PVS) Gap Analysis (2013). Current tools and protocols are largely based on IHR (2005) MEF, except the PVS tool. Liberia successfully participated in SPAR in 2018 and reported C9 - Health Service Provision capacity, with a score of 33% which is below regional and global averages. There is currently a limited integrated approach to measure quality and preparedness aspects in health services; limited SimEX exercises and screening at Ports of Entry (POE) due to limited support.

There are several relevant stakeholders in the system to support the integration of quality and emergency for health system resilience. For example, at the national level there is the Healthcare Quality Management Unit (HQMU) within the MOH, National Public Health Institute of Liberia (NPHIL), Environmental Protection Agency (EPA) and National Disaster Management Agency (NDMA). At the subnational level: County Health Teams (CHT), County Health Boards (CHB), District Health Teams (DHTs), Health facilities (public and private), Health workers including Community Health Workers such as community health assistant, general community health volunteers etc. Also, there are academic and professional training institutions as well as regulatory agencies like the Liberia Board of Nursing and Midwifery (LBNM), Liberia Medical and Dental Council (LMDC). Partner organizations constitute an important group of stakeholders to integrate quality and emergency in Liberia. However, there is currently fewer partners supporting the integration of quality and emergency preparedness in Liberia as compare to 3-4 years ago (donor fatigue).

There are several important assets across the health system for the integration of quality and emergency. For example, 1) previous and existing training programs (e.g. FETP, SQS) can inform an integrated approach to trainings, 2) availability of an electronic integrated monitoring and measurement tool for data collection and supervision, 3) availability of trained workforce in Field Epidemiology, some VHF isolation and management capacity, 4) existing QI practices: IPC, AMR and WASH practices, supervision and coaching, 5) the presence of a National Reference Laboratory (NRL) with two operational regional labs, 6) existence of SOPs and guidelines (IPC, WASH, IDSR, specific disease contingency plans), and 7) Strong interest amongst stakeholders (e.g. LBNM, LMDC) for integrating healthcare quality with public health emergency preparedness.

Despite some assets for integrating quality and emergency, several critical challenges have been identified with the potential to impair the integration of quality and emergency preparedness in Liberia, including: (1)

inadequate workforce capacity with few health professionals, who are unequally distributed; (2) limited capacity of frontline health workers in emergency preparedness such as SimEx; (3) weaknesses in the management of the healthcare systems; (4) weak multi-sectoral coordination of actors/partners; (5) limited emphasis placed on the dimensions of quality and emergency in the training curricula of medical, nursing, midwifery and laboratory trainees, including the absence of systematic Continuous Professional Development (CPD) for in-service personnel; (6) inadequate diagnostic capacity at healthcare facilities, including the NRL, especially the pathogen detection capacity and improve information management system (the newly created regional laboratories need investment to be fully operational); (7) weak information management architecture of the health system (paper-based and electronic data systems) and (8) the need for behavioral change to improve adherence to guidelines, discourage territorial interest and fragmented approach to project implementation by both national and international agencies.

At the end of the country situational assessment, a stakeholders' consultative meeting was organized to validate the findings from the assessment to confirm and consolidate the information presented through reviews, additions, recommendations and refinements, and mapping of the project next steps. This meeting was marked by huge enthusiasm from participants and the HSR project pilot sites and next steps were clearly articulated and endorsed. The results demonstrate that there is a consensus amongst stakeholders for the project support to enhance resilience in health system with quality and emergency preparedness.

1. Introduction and Context

1.1. Geography, Population and Demography of Liberia

Liberia is on the West Coast of Africa. It is bordered by Sierra Leone to the west, Guinea to the northwest, Ivory Coast to the northeast and east and the Atlantic Ocean to the south. It covers an area of 111,369 square kilometers and 43,000 square miles with an estimated total population of 4.94 million people (2019), with over half of the population living in urban areas[1]. Liberia is a low-income country with an estimated GDP per capita of USD 674 (2018), a decrease of USD 24 from 2017 (when it was USD 698)[2]. Liberia's economy expanded by an estimated 1.2% in 2018, a significant slowdown from a growth rate of 2.5% in 2017. This is evident by the all-time high inflation rate 28.5% recorded by the end of December 2018[3].The country's Human Development Index value for 2018 was 0.435, which puts the country in the low human development category—positioning it at 181 out of 189 countries[4].

According to the United Nations Human Development Index (HDI) Report (2018), life expectancy in Liberia is 63.0 years. Liberia has a neonatal mortality rate of 38 deaths per 1000 live births, while the infant mortality rate is 22 deaths per 1000 live births and under-five mortality of 94 deaths per 1,000 live births. Meanwhile, the maternal mortality ratio stands at 1,072 deaths per 100,000 live births. HIV prevalence rate among adults aged 15-49 was estimated at 1.9 %[5]

1.2. Liberia Health system

The country is divided into five (5) regions and 15 political sub-divisions called counties. These also correspond to the 15 health counties, which are further divided into 92 health districts[3]. The health system is organized into three tiers: primary, secondary and tertiary. The recent Service Availability and Readiness (2018) showed that there are 831 health facilities across the 15 counties in Liberia, with a majority (55%) of them being public facilities[6]. There are relatively few hospitals (5%), and health centers (7%) in the country. Clinics make up the majority (88%) with an almost equal distribution between rural (49.6%) and Urban (50.4%) areas. Most of the population is within close proximity to health facilities as demonstrated by a health facility density of approximate of 2 per 10,000 population. However, deplorable road conditions, limited referral facilities and the unavailability of an efficient public transport system have been identified as major obstacles to accessing health facilities[7].

---

[1] World Population Prospects. (2019 Revision). United Nations population estimates and projections, available on http://worldpopulationreview.com/countries/liberia-population/. Accessed 15 July 2019.
[2] https://countryeconomy.com/gdp/liberia, Accessed 4 October 2019
[3] https://www.worldbank.org/en/country/liberia/overview
[4] United Nations Development Program (2018). Human Development Indices and Indicators, statistical updates http://hdr.undp.org/sites/default/files/2018_human_development_statistical_update.pdf. Accessed 15 July 2019.
[5] WHO country cooperation strategy, Liberia 2018
[6] MOH. 2018. Service Availability Readiness Assessment (SARA)
[7] Ibid

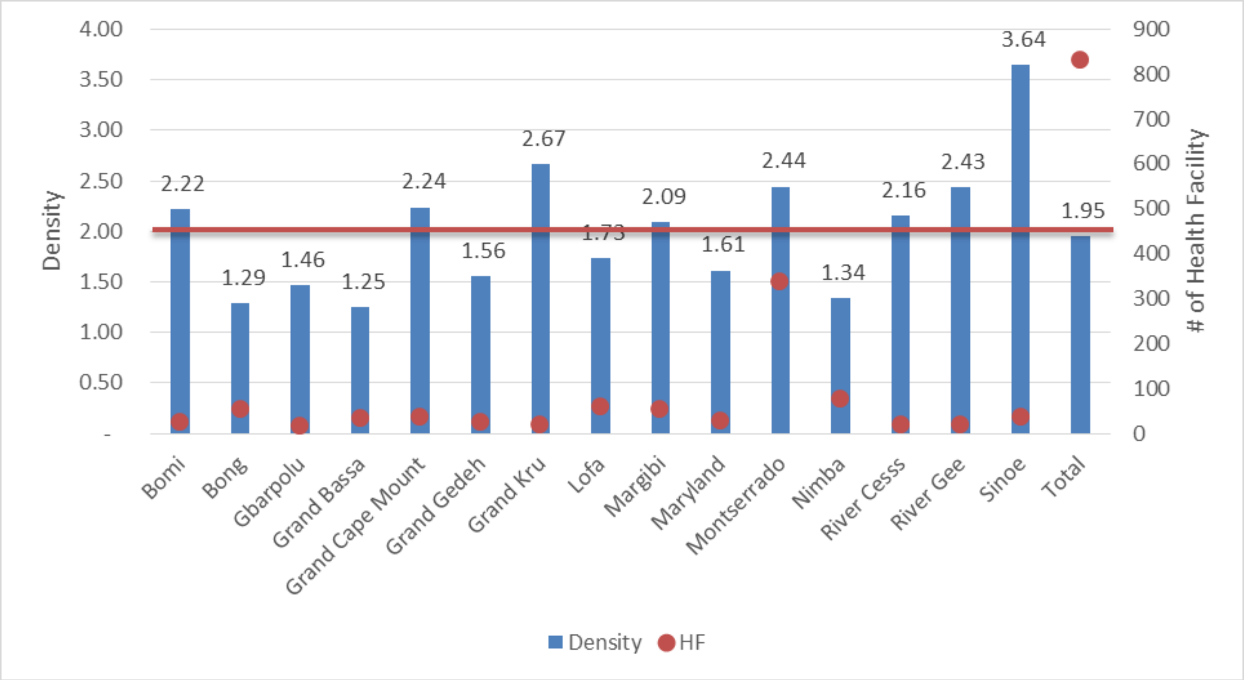

Figure 1: Health Facility Density by county per 100,000 population, SARA 2018

The Liberian health system has experienced many shocks in past and recent times. About ten years after a 14-year civil war, Liberia suffered an Ebola virus disease (EVD) outbreak which lasted for almost three years (2014 – 2016). These outbreak, and other events, have highlighted the critical importance of quality essential health services with emergency preparedness of health systems. As seen during the 2014-15 Ebola outbreak, poor preparedness, coupled with weakened health systems, accounted for about a 50% decline in routine health services delivery and the death of over 500 health workers[8]. These and many other incidences motivated the launching of the Global Health Security Agenda (GHSA) to cause countries to commit to the revised IHR (2005). Quality has been highlighted as a key element in healthcare delivery for Universal Health Coverage[9]. Therefore, an integrated approach to develop and sustain quality and emergency preparedness in tandem is necessary to ensure health system functionality to maintain routine and emergency related healthcare and response.

The Korean International Cooperation Agency (KOICA) funded five-year project (2018 – 2023) on Building resilient health care services to improve emergency preparedness in Liberia aims to strengthen and build resilient health care services to improve emergency preparedness, whilst maintaining quality routine health service provision. An important mile stone in the realization of this project is the situational assessment on the state of quality and emergency preparedness

[8] Brolin Ribacke KJ, Saulnier DD, Eriksson A, von Schreeb J. Effects of the West Africa Ebola virus disease on health-care utilization–a systematic review. Frontiers in public health. 2016 Oct 10; 4:222

[9] Kruk ME, Gage AD, Arsenault C, et al. High-quality health systems in the Sustainable Development Goal era: time for a revolution. Lancet Glob Health; 2018.
WHO community engagement frameworks for quality, people-centered and resilient health services. Geneva: World Health Organization; 2017.

and response in the country. This report aims to highlight findings on quality and emergency preparedness from the situational assessment conducted in Liberia as part of the KOICA Project.

The project has also undertaken a global desk review which complements this report by providing a global perspective on the same topic.

2. Objectives of the situational assessment

The overall aim of the situational assessment was to ascertain the current state of quality and emergency preparedness and their integration to build health service resilience in Liberia.

Specific objectives

1. To review national to subnational health sector policy and planning from quality and emergency preparedness perspectives of health services
2. To elucidate current state of implementation of relevant national policy, plan and programs in making health services resilient
3. To examine the application of tools and measurement approaches in quality and emergency preparedness of routine health services
4. To identify authorities and stakeholders and their role and influence on quality care and emergency preparedness in the context of health service
5. To elucidate levers, barriers, gaps and opportunities to integrate quality and PHE preparedness and response within the Liberia health service delivery
6. To propose a list of facilities (applying a catchment approach) for operationalization of the project

3. Method

In order to gain an understanding of the state of health service quality and emergency preparedness considerations in Liberia health system, a systematic, multi-dimensional approach was necessary in conducting the situational assessment. To guide the process, a Terms of Reference (ToR) was developed with objectives and methods to be applied.

The multi-dimensional, systematic approach applied in conducting this situational assessment involved;

- A **desk review** to secure a foundational understanding of the health system of Liberia by analyzing key national documents on quality and emergency. Selection of the various documents was informed by MOH and NPHIL engagement, including periodic meetings with WHO project team
- **Stakeholder consultations** and interviews with key actors from across all levels of the health system and related institutions e.g. MOH, county, district, training institutions.
- **Health facility visits**, including hospitals, clinics and health centers across three selected counties (Bong, Cape Mount and Lofa). These involved both stakeholder consultations and observations on aspects of quality and emergency preparedness and response guided by a site selection matrix and a site assessment tool developed by the project team.

3.1. Desk review

The project team conducted a wide search using key search terms and questions to identify and review relevant publications and documents from international and national databases including MoH, NPHIL, other line ministries and WHO. Key persons from MoH, NPHIL, WCO, and other partner organizations were also consulted to collect relevant documents which were not in the public domain. These consultations included joint technical working group sessions between WHO project team and government-designated project focal persons from MoH and NPHIL (Annex I). Materials identified included government policies, plans and strategies, assessment and evaluation reports and training curricula relevant to health systems resilience from quality and emergency preparedness perspectives. The review focused on documents relevant to Liberia context and covering the period from 2015 (post-Ebola) to September 2019, to include recent reforms of the MoH and related agencies and structures.

Table 1: Summary of reviewed documents

Category	Reviewed
**Policies & legislations**	21
**Plans & strategies**	16
**Technical reports**	24
**Other technical documents (manuals, SOPs, guidelines etc)**	18

3.1.1. Search strategy

To search for relevant documents in the health databases, key search terms based on the focus of the review topic were used including; health emergencies, disease outbreaks, epidemics, pandemic, emergency preparedness, emergency readiness, quality health services, quality of care, quality improvement, safe health services, people-centered care, resilience, health system resilience, health system strengthening, integration, effectiveness, and health security. The key words were combined using Boolean Operator, AND, OR, quotation marks, and variants of terms as applicable[10]. For online database search (Medline, PubMed, Google scholar, Embase). Moreover, grey literature was searched for relevant documents mostly through key stakeholder recommendations (snow-balling).

The search yielded over 100 documents, of which 60 were retained for review. From the latter, 11 were peer-reviewed articles (Google Scholar-5, pubmed-4; Embase-2) and the rest grey literature. Over half of the papers (n=7; 63%) were discarded for duplication and non-relevance to the subject matter. Five potentially relevant studies were identified from citations in reviewed articles.

Table 2: Inclusion and exclusion criteria

**Inclusion criteria**
English language
Relevant to post-Ebola period (2015 to date)
Relevant to Liberia context
**Exclusion criteria**
Not relevant to public health emergency resilience
Not relevant to health services quality
Not relevant to health service preparedness

Some of the selected and reviewed documents are listed and analyzed in a synthesis table (Annex II) to identify their relevance to; emergency preparedness and response, health service quality (safety, effectiveness, person-centeredness), health system resilience and the notion of integration between quality and emergency. The documents were identified under six different categories; policies, reports, strategies, plans, guidelines and others (manuals, protocols, SOPs) (table 1). Documents in each category were jointly reviewed by the Technical Working Group (TWG), and qualitative and quantitative data extracted to develop this situation assessment report.

A generic scale (table 3) was developed and applied in the review process to appraise policy and strategy documents and other relevant documentation identified in the desk review.

[10] An Roinn Sainte. (2013). How to conduct a literature search? Internal staff guide

Table 3: Criteria and rating scale used in the appraisal of documents during the review

Rating	Integrated approach to Quality of Care (QoC) and Health Facility Emergency Preparedness in national policy, plan and services
3	Evidence for an integrated and coherent approach to priority QoC domains (safety, effectiveness, people-centeredness) with health facility level preparedness identified
2	Reference to an integrated approach identified between documentations but lacks in coherence to operationalize priority QoC domains (safety, effectiveness, people-centeredness) with health facility level preparedness in tandem
1	Reference to priority QoC domains (safety, effectiveness, people-centeredness) or health facility level preparedness identified between documentations in isolation without any coherence
0	No reference to Quality of Care (QoC) and Health Facility Emergency Preparedness in national policy, plan and services

Coherence in this context referred to evidence for a consistent emphasis to consider QoC and emergency preparedness in tandem from national legislation, policy, plan, strategy to operational materials. Strategy and Operational materials in this context are what will be implemented at health facility level or/by district heath management team in reference to clearly defined national policy and legislative requirements.

3.2. Stakeholders' Consultations

The situational assessment involved engagement with and participation of national and subnational stakeholders including leadership and technical officers from relevant health system and emergency units of MoH, NPHIL, WCO, professional bodies in health, partner organizations, county health teams, district health teams, health facility management and healthcare workers. Through email and face-to-face communications with various stakeholders, the project team gathered information on previous, ongoing and planned initiatives related to health service resilience (quality and preparedness) in the country. These consultations also informed the selection of project sites and the approach for the field visit. To finalize this report, a stakeholders' consultative meeting was held to further validate findings of desk review and field assessment and also confirm the first wave of health facilities for the project pilot. Feedback from the workshop has been incorporated into this final situational assessment report.

Table 4: List showing stakeholders consulted as part of situational assessments

Level	Stakeholders Organization	Position and Role	Consultation Objectives
**National**	MoH Quality Management Unit (QMU) and related departments like research, human resource	Leadership (Assistant Minister, Directors etc.) and technical focal points	• Examine how quality and preparedness are placed within the Liberian health systems<br>• Explore the role of quality and emergency preparedness settings<br>• Explore the interlinkages between the health service delivery, public health and animal sectors in relation to emergency preparedness and resilience
	NPHIL	Leadership (DGs) and technical focal points	• Explore the interlinkages between the health service delivery, public health and animal sectors in relation to emergency preparedness and resilience
	WHO country offices (Health Systems /Essential Health Services, emergency program)	Leadership (HSS team lead, WHE team lead etc.) and technical focal points	• Identify and discuss WHO's role, recent and ongoing efforts in integrating quality and emergency preparedness at the service delivery level
	Health professional councils *(education, licensing, accreditation, registration etc.)*	Nursing and midwifery board officers	• Discuss the role of professional councils in ensuring quality health services delivery and institutionalizing quality and emergency preparedness in training curricular
	NGOs/Partners implementing quality and emergency initiatives	Leadership and technical focal points	• Identify and discuss recent, ongoing and planned initiatives by partners in improving health systems resilience by integrating quality and emergency preparedness in health services
**Sub-national**	County Health Teams	County Health Officer, County Surveillance Officer and technical teams and	• Discuss implementation of national policies and plans in relation to quality of care and emergency preparedness<br>• Examine regional policies/strategies for emergency preparedness and quality of health

Level	Stakeholders Organization	Position and Role	Consultation Objectives
		other technical officers	services from the perspective of health service resilience<br>• Explore the interlinkages between the health service delivery, public health and animal sectors in relation to emergency preparedness and resilience
	District Health Teams	District Health Officer, District Surveillance Officer, Leads and technical officers in quality and other related programs	• Examine facilitation role of districts and operational plans for QI practices and emergency preparedness<br>• Explore the state of consideration and integration of quality and emergency preparedness at the district level<br>• Explore the interlinkages between the health service delivery, public health and animal sectors in relation to emergency preparedness and resilience
**Health facility**	County/District hospitals**<br><br>Clinics**<br><br>Primary Health Care facilities** *(public, private, faith-based)*	Heads or focal persons responsible for quality and public health emergency activities	• Discuss the various QI measures in the hospital, the role of the hospital in PHEP&R, and the bridge between these two aspects<br>• Investigate measures for quality management in practice<br>• Discuss the quality of routine health services and the role of the hospital in health emergency preparedness and response<br>• Examine areas related to safety, people-centeredness and clinical effectiveness for quality of care<br>• Understand barriers, enablers, gaps and opportunities to apply QI measures to enhance emergency response

***Public, Private-for-profit, Private-not-for-profit*

At the national level, the key stakeholders consulted included; 1) the leadership of the Healthcare Quality management Unit (QMU), Ministry of Health which is in charge of quality planning, control and improvement in the health system of Liberia; 2) the Liberia Medical and Dental Council (LMDC) which has a mandate to accredit healthcare facilities (public and private), 3) Liberia Board of Nursing and Midwifery (LBNM) which develops and reviews training curricula and regulates nursing and midwifery practices and 4) the Liberian Medical College (i.e. Liberia College of Physicians and Surgeons (LCPS).

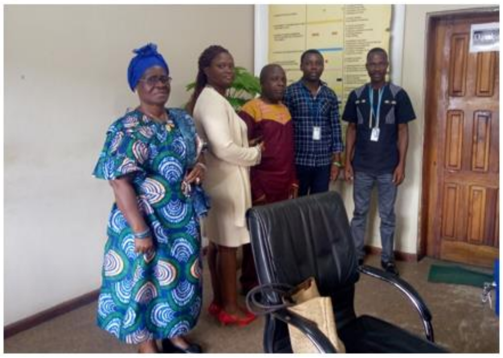

Photo 1: Consultation with LBNM

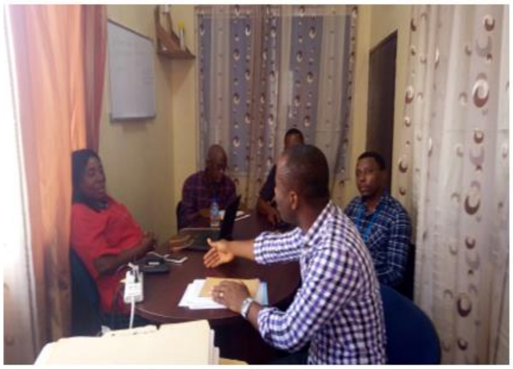

Photo 2: Consultation with LMDC

Engagement with nursing & midwifery trainees from the Tubman National School of Medical Arts (TNIM) and medical students from A.M. Dogliotti College of Medicine, University of Liberia, alongside their tutors revealed existing gaps in quality improvement in health service delivery, including teaching curricular on patient safety concepts in the Liberian academic programme for health personnel. The stakeholders express the paucity of quality and emergency preparedness and response in the training curricula for health personnel.

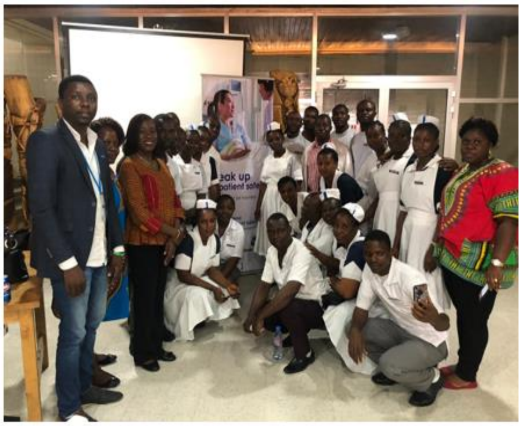

Photo 3: Engagement with TNIM trainees

At the subnational and community levels much remains to be done to ensure effective participation of communities and patients in the decision-making process in routine healthcare delivery, especially concerning quality. However, some donor organizations like the Partnership for Advancing Community-based Health Services (PACs) support Community Health Workers to conduct community surveillance for diseases with epidemic potentials.

Group discussions with Civil Society Organizations (CSOs) identified the need to provide patients and their communities with the right knowledge on patient rights and safety, and develop mechanisms to capture the experiences of patients in health facilities. CSOs and CBOs expressed their readiness to fill this void if provided with training, material and financial resources.

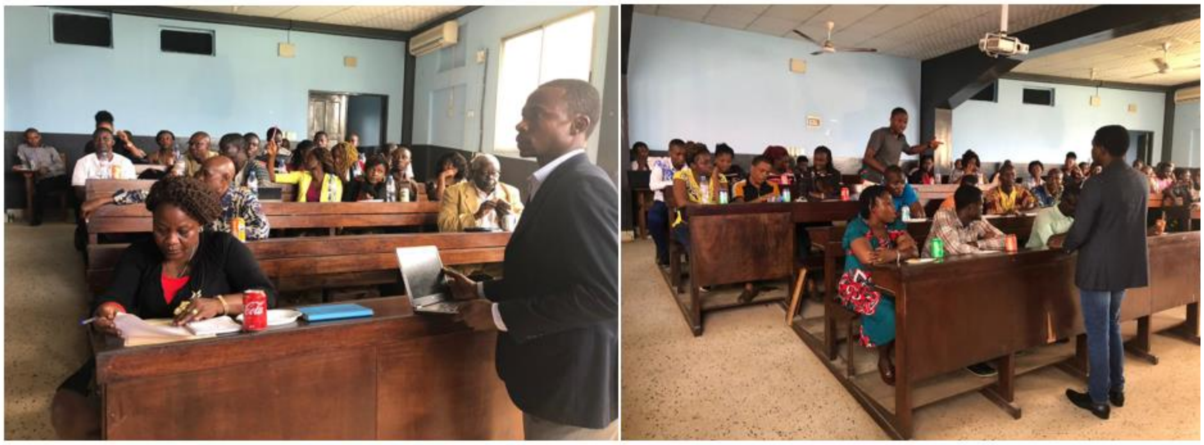

Photo 4: Consultation with medical students and lecturers

3.3. Project site selection and situational assessment

3.3.1. Catchment and System Approach

A catchment/system approach was applied in the selection of health facilities for operationalizing the project at all levels of the health system, while supporting interlinkages with human/public, environmental and animal health functions. This will enable a systematic consideration of quality and emergency preparedness in project implementation; starting with three counties in the first phase and gradually scaling-up as resources allow. Below is the project's concept for a catchment approach (simplified schematics) in site selection to embed quality and emergency at different tiers of health services in coordination with Public Health and Animal Health.

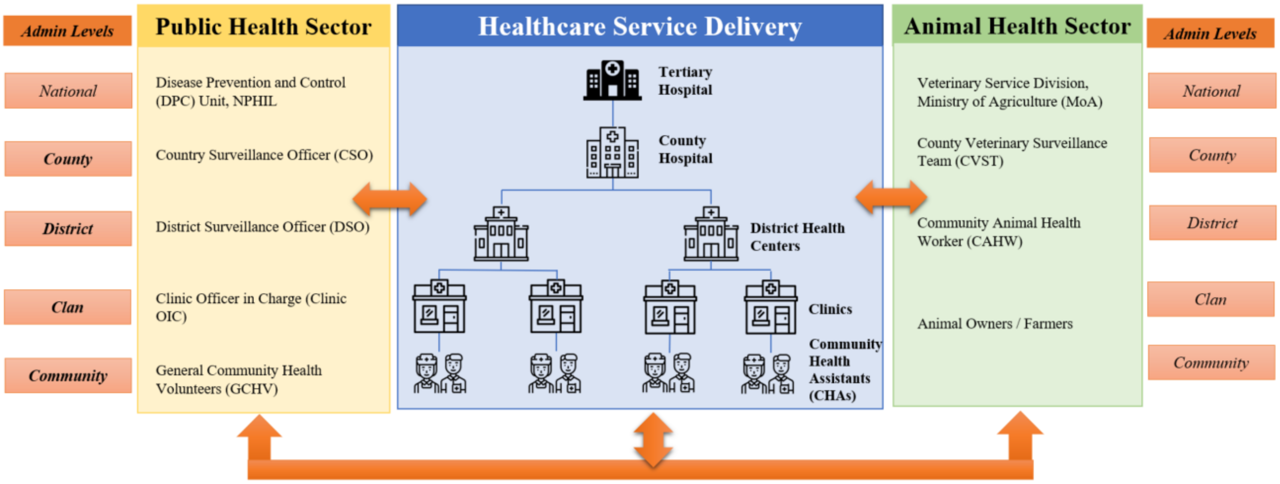

Figure 2: Catchment and system approach across relevant sectors and administrative levels

Legend

Public Health Functions

DPC: Disease Prevention and Control; CSO: County Surveillance Officer; DSO: District Surveillance Officer; Clinic OIC: Clinic Officer in Charge; GCHV: General Community Health Volunteers;

Healthcare Service Delivery

District HC: District Health Centre; CHA: Community Health Assistant

Animal Health Functions (for Zoonotic Disease)

MoA: Ministry of Agriculture; CVST: County Voluntary Surveillance Team; CAHW: Community Animal Health Worker

3.3.2. Site Selection Matrix and Criteria

The project site selection matrix incorporating selection criteria was used to map health facilities in the country while applying a catchment approach. Data from the matrix was used to guide the selection of counties and project sites (facility), while taking into considering the status and focus of other initiatives being implemented in these counties to avoid duplication and identify potential opportunities for collaboration. The selection criteria considered in selecting the health facilities to implement the initial stage of the KOICA project were as follows:

1. **Burden of emergencies**: Health facilities in counties which are currently or were recently affected by a public health emergency were prioritized.
2. **Stability & access**: Facilities were selected from counties and districts which are politically, socially and economically stable. Geographical assess to the health facilities was also considered.
3. **Local interest**: The level of interest demonstrated by the county and district health teams and health facility managers was taken into account during the selection process. We selected health facilities from which the interest from management was high.
4. **Basic infrastructure and functionality**: Selected health facilities are supposed to be functional with basic essential supplies, equipment and human resources.

Site selection matrix (KOICA project: Making Health Services Resilient)																								
Facility	Catchment														Ongoing Efforts in Health Service Strengthening and Emergency Preparedness								Criteria for Consideration in project implementation				
	Level					Location			Rural/Urban		Ownership			Population	Description of Initiative						Status / Duration of						
Name	Tertiary	Secondary	Primary	Number of health centers in the	Number of clinics in the catchment	County name	District name	Clan/zone name	Urban	Rural	Public	Private-for-profit	Private-not-profit	Size of population serving	Program/Project/ Initiative name	Related thematic area of focus (quality	WHO-led: Name of unit, objectives	Partner-led: Name, objectives and	MoH-led: Name, objectives and scope	Multiple partner led Names, objectives,	Ongoing?	If yes, duration	Stability: Currently affected by PHE or	Authorities are interested to participate	Basic infrastructure are in place	Site and facility are stable and	Proposal for selection?

Figure 3: Site selection matrix

Based on the site selection matrix, MoH and NPHIL consultations, three counties (Bong, Lofa and Grand Cape Mount), were prioritized for the first phase of the project operationalization. Through onsite engagement of county, zonal and district health teams, 19 health facilities (ten clinics, five hospitals and four health centers) were selected (table 5).

Table 5: Selected health facilities for the field visit of the situational assessment

County	District	Health Facility	Type	Ownership
Bong	Jorquelleh	Baptist clinic	Clinic	Private non-profit
		CB Dunbar	Hospital	Public
	Kpaai	Palala	Clinic	Public
	Salala	Salala	Clinic	Public
	Suakoko	Phebe	Hospital	Private non-profit
	Zota	Belefanai	Health Centre	Public
GCM	Common Wealth	Madina	Clinic	Public
		St Timothy	Hospital	Public
	Garwula	Sinje HC	Health Centre	Public
	Tewor	St. Tienni	Clinic	Public
		Bo Water	Clinic	Public
		Devos	Health Centre	Private non-profit
Lofa	Kolahun	Korworhun	Clinic	Public
		Bolahun	Health Centre	Public
		Kolahun	Hospital*	Public
	Voinjama	Bondi	Clinic	Public
		Barkedu	Clinic	Public
		Free Pent	Health Centre	Private non-profit
		Tellewoyan	Hospital	Public
**3**	**10**	**19**		

**District hospital*

3.3.3. Health facility visit and assessment

The choice of health facilities for the field assessment was informed by an initial project site selection done by applying the catchment approach as elaborated in the previous section. The field visit was to consolidate the findings made from the desk review and the stakeholders' engagement. This exercise was an important component of the project to elucidate the current state of quality and emergency preparedness at the facility level. This segment of the situational assessment report includes; the health facility selection matrix, baseline data from the three selected counties, aspects of quality and emergency response, barriers and levers for integration of quality into emergency response for resilience as these applies in the field. These findings will inform alignment of project activities to current capacities and gaps related to health services resilience in Liberia.

Health facilities visits and assessment involved three teams, each to carry out assessment in Grand Cape Mount, Lofa and Bong counties, respectively. Each team consisted of technical officers from the MOH, NPHIL and WCO Liberia, and two county officers; a clinical coordinator and a disease surveillance focal person. A jointly-developed assessment tool (Annex III) was used by the teams to guide consultations and interviews with the County Health Teams (CHT), District Health Teams (DHT) and health facilities. The assessment adopted individual interviews, small group discussions and observations of the health facility and practices, where appropriate.

4. Findings of the Situational Assessment

4.1. Review of legislations, policy and planning documents from quality and emergency preparedness perspectives

The findings from the desk review are presented in the table in **Annex II**.

Highlights from the desk review

- Over 100 documents were searched for relevance in quality and emergency preparedness in Liberia.
- 79 documents were selected for the review to which the appraisal criteria (table 4) was applied.
- Out of the 79 documents reviewed, 36 were policies/regulations/legislations and plans. From the plans and policy documents/regulations/legislations reviewed; 35 (97%) highlighted QoC in isolation, 24 (67%) made mention of emergency preparedness and response only, while 12 (33%) mentioned an integrated approach to QoC and emergency preparedness.

The review of relevant legislation and policies showed that:

There are national legislations and policies to support initiatives in building health services resilience by improving quality improvement and emergency preparedness.

However, quality and emergency preparedness are generally considered separately in national legislations and policies. There is need for an integrated approach to quality and emergency preparedness to strengthen resilience of the health system to respond to emergencies.

Noted limitations and challenges of the desk review included; 1) the limited access to some of the relevant documentation. For example, absence of reports on users' experience of care and a crashed online directory for health workforce distribution (IHRIS), 2) there were no documents from the Quality Management Unit (QMU) of the MOH elaborating quality of care indicators, 3) multiple versions of the same document produced by different institutions (duplication).

4.2. Current state of implementation of relevant policies, plans and strategies on health service quality & emergency preparedness

Various national plans have been developed to guide implementation of the above legislations and policies as outlined in **Annex IV**. Most of the plans maintain a parallel focus on either health service quality or emergencies preparedness and response. However, the Investment Plan for Building Resilient Health System incorporates health services quality and emergency preparedness as key elements for building health system resilience. The following aspects were analyzed.

4.2.1. Health service quality

The National Health Policy and Plan (NHPP) is designed to be fully implemented by 2021, with incremental achievements per year based on yearly operational plans. The Liberia health system resilience investment plan is based on the NHPP and lessons learnt from the 2014-2015 Ebola outbreaks. It prioritizes restoration and enhancement of health services quality for patients and health workers' safety. Progress has been made in this area of investment, including; establishment of the HQMU-MoH, development of National Health Quality Strategy (NHQS) and national IPC guidelines. Infection Prevention and Control (IPC) and WASH structures and standards, monitoring and supervision have also been integrated into routine health services as part of quality improvement measures and considered as part of emergency preparedness measures. The country is also in the process of developing guidelines for safe management of healthcare waste. However, training and monitoring of healthcare services based on the recently developed IPC guidelines and NHQS are yet to be accomplished; this has hampered the implementation of these guidelines in health facilities. The 2018 evaluation of the health sector (2006-2017) found that critical gaps persist in the quality of health services, including observation that recent increase in skilled health workers did not improve the quality of care and outcomes e.g. maternal and child health services.

4.2.2. Emergency preparedness

One of the main areas of investment for building health system resilience is in strengthening epidemic preparedness, surveillance and response[11]. In line with priorities identified for this investment area, Liberia established the National Public Health Institute of Liberia (NPHIL) and a Public Health Capacity Building Center, and EOCs /IMS at national and county levels as core structures for the implementation of IHR (2005). The Integrated Disease Surveillance and Response (IDSR) and the Early Warning, Alert and Response Networks (EWARN) have also been established at national, county, district and community levels. As of 2016, 11 out of 15 counties were implementing Community Event-Based Surveillance (CEBS) through trained community

[11] Ministry of Health (2015). Investment plan for building a resilient health system 2015-2021. Republic of Liberia

volunteers[12]. Over 92 district surveillance officers, 22 zonal surveillance officers and 15 county surveillance officers have been trained in Field Epidemiology Training Program[13] (FETP)**.** National and county EPRPs are in place and based on the national EPRP, rapid response mechanisms have been established at national, county and district levels[14]. However, there is limited information on healthcare facility-level preparedness activities.

1.2.3. Laboratory services and emergency preparedness

Laboratory diagnostic capacity can help in detecting emerging or re-emerging pathogens in a timely manner and can support the diagnostic capability of a country. For years, the public-sector laboratory services and systems have been severely neglected in Liberia, and this was further weakened by the 2014 Ebola Virus Disease (EVD) outbreak across West Africa[15]. The EVD outbreak demonstrated the critical role a well-coordinated and operational public health laboratory network plays in detection and response to emerging infectious diseases. This, however, can be best optimized within a supportive network that encompasses human, environmental and animal sectors, to promote integrated health systems strengthening for health security.

Referral system

The Public Health specimen transportation system for Liberia is under-resourced. Specimen transportation within the country is conducted by Riders for Health (Riders). Using a linked pathway, specimen from pick-up sites are delivered to the respective testing laboratories. CHTs and partners support transportation of specimens from peripheral facilities to the pick-up sites for onward transportation by Riders. Transportation of specimens for international testing or quality control is conducted through international courier agencies including DHL, world courier, among others, when needed. The road network in Liberia is not very well maintained and it can be very difficult to transport materials in a timely manner, especially during the rainy season. The poor road network in remote areas negatively impacts on timely specimen referral for testing, especially during the rainy season. Cold-chain management, collection and transportation of samples continue to be challenges.

In 2017, a total of 2365 specimen were collected across all 15 counties for laboratory testing for 8 of the 9 immediately reportable epidemic prone-diseases requiring laboratory confirmation, including: Ebola Virus Disease (EVD), Meningitis, Severe Acute Watery Diarrhea (SAWD), Acute Bloody Diarrhea (ABD), Measles/Rubella, Yellow fever, Lassa fever, and Acute Flaccid Paralysis (Polio). Overall, 50% and 25% of alerts received laboratory testing (results) within four days and

[12] (Joint Annual Health Sector Review, 2016).
[13] Join Annual Health Sector Review, 2016
[14] Join National Action Plan for Health Security, 2018-2022
[15] Ministry of Health (2019). Five year strategic plan for the national laboratory system of Liberia, 2019-2024

seven days of alert notification, respectively (figure 1). The overall turn-around time improved by 15%, from 35% of alerts being confirmed or ruled-out within four days in the first half of the year to 50% overall by the end of the year [16.]

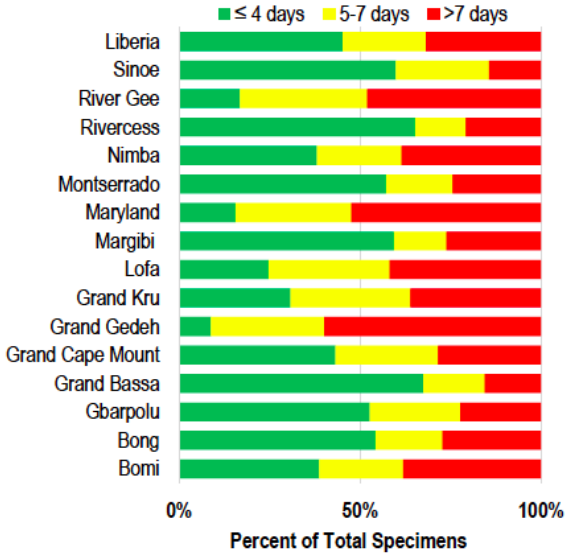

Eighty-five percent (85%) of all the specimens collected from alerts reached the respective laboratories within 72 hours from the time of specimen collection (Figure 24). A proportion of specimens from some Counties, namely; Grand Gedeh, Grand Kru, Lofa, Maryland, Rivercess and River Gee, took longer to reach the testing laboratories mainly due to longer distance to the testing laboratories as well as very challenging road conditions. [17]

Figure 4: **Duration from sample collection to testing for IDSR priority disease by County, Liberia (January – December 2017)**

State of interlinkages and capacity in laboratory services - human and animal health

Formal linkages (in terms of laboratory information sharing) between the animal health and human public health sectors are currently unclear. While there is appreciable human resource capacity in human health at national level, animal health is grossly under-resourced[18]. Animal disease testing and surveillance capabilities are limited owing to a lack of infrastructure and equipment. The One Health approach is therefore a critical area that needs improvement. Liberia is exploring ways of retaining trained animal and human health personnel, such as FETP graduates and those that were trained during the recent EVD outbreak, through mobilization of Government and donor funding, as well as offering contracts which engages/compels the trained personnel to serve the government for a specified time period (bonding).

There is a need to collaborate with training institutions to review pre-service training curricula to ensure that One Health, IHR (2005), IDSR and disaster management are addressed. There is need to clearly define linkages between private sector laboratories, clinical and public health laboratories, with veterinary laboratories. The government aims to improve communication about zoonotic diseases by designating a representative from various sectors, including the

[16] Ibid
[17] Ibid
[18] Ibid

Ministry of Health, Ministry of Agriculture, Forestry Development Authority and Environmental Protection Agency, to attend One Health Coordination Committee working sessions.

Current surveillance for animal bites (suspected rabies) under IDSR has established a “link” between human and animal health through collaboration between the MOH and the Ministry of Agriculture (MOA). However, the practice of information sharing is inconsistent, non-systematic or inadequate. Much remains to be done to facilitate inter-sectoral collaboration between animal and human health. However, great efforts are being made for all laboratories including public health and veterinary services to operate under the One Health framework. There are ongoing efforts to upgrade the National Reference Laboratory (NRL) to level 3 biosafety standard and national laboratory policies and strategies. The country has also improved the capacity of public health laboratories and established a national biobank.

Despite the above achievements, inadequate logistics and supplies, suboptimal isolation unit capacity, inadequate resources to conduct and scale-up simulation exercises, poor data management systems, human resource shortage, coupled with poorly motivated health workforce, insufficient funding, poor inter-sectoral coordination remain some of the major challenges impairing the optimization of emergency preparedness in the health system[19].

4.2.3. Health workforce

Health workforce density

According to Health Sector Performance Evaluation Report (2006-2017) the MOH is on course in achieving its target of employing 15, 000 healthcare workers between 2011 and 2021. By the end of February 2016, MoH had employed a total of 10,406 healthcare workers. Health workforce density is an important indicator for measuring health workers’ availability with reference to the population. WHO global target for health workers’ density for 10,000 population is 23. The current national health worker density per 10,000 population is 11.7 with variation across counties (SARA, 2018)[20].

[19] Ministry of Health (2018). Health Sector Performance Evaluation Report (2006-2017). Republic of Liberia

[20] 2015/2016-health workforce census in Liberia.

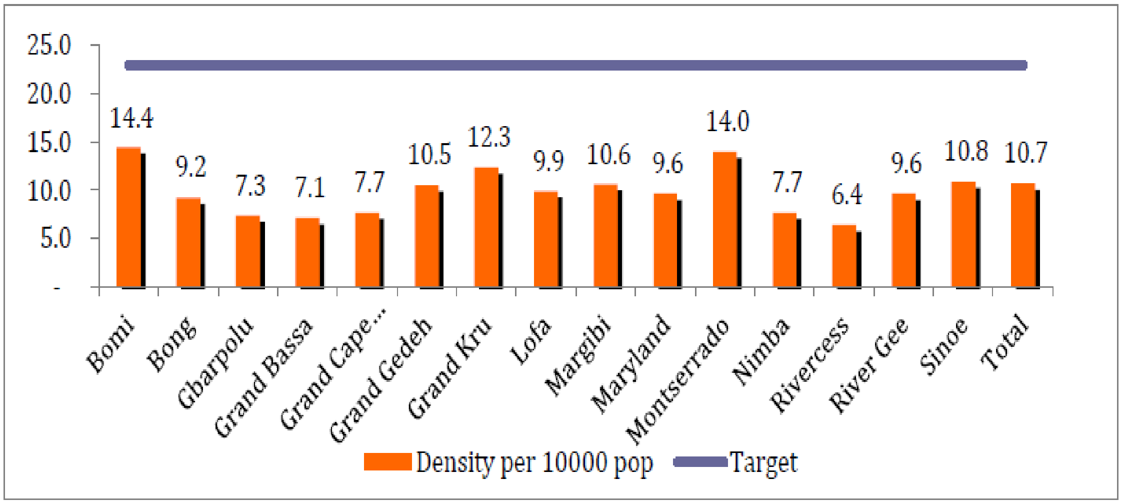

Figure 5: Core health worker density per 10,000 pop per county, SARA 2018

Projections from the National HR for Health policy and plan estimated a total of 8,742 skilled professionals (55% of total workforce) by 2021, and health workforce density of 19.2 per 10,000 population. There was an increase in clinical health workforce density from 8.6 in 2015 to 11.8 in 2016, based on most recent health workforce population census (2016). The 2018 SARA reported a density of 10.7 clinical health workers per 10,000 population. The statistics remains far below the WHO recommendation.

Health workforce distribution

The highest number of health workers was found in Montserrado, Nimba, Lofa and Bong counties. However, these counties also have the highest concentration of population as well as health facilities distribution in Liberia. Clinical health workforce (including Aides and health technicians) constituted over half (56.4%) of the overall workforce. The census recorded 4,756 core clinical health workers (Midwives, Nurses, Physicians and Physician Assistants) across the 15 counties of Liberia in both public and private facilities. Registered Nurses accounted for the highest number of core clinical workers 64.7% (3,077/4756), followed by midwives (19.5%), physician assistants (10.9%) and physicians (4.9%). Four counties namely: Montserrado, Nimba, Bong and Lofa accounted for 68.2% of the workforce, with Montserrado alone hosting 30.6%. This shows existing imbalances in the availability and distribution of various cadres to meet the health sector needs.

In its HRH policy/plan document (2011-2021), the MoH highlighted health workers' deployment to rural areas as a major challenge because of inflexible salary scales and generally inadequate incentives to retain skilled providers in remote communities, where the need is critical. Based on the recent Health Sector Performance Evaluation (2006-2017)[21], while there has been significant

[21] Ministry of Health (2018). Health Sector Performance Evaluation Report (2006-2017). Republic of Liberia

health workforce improvement, improving retention of skilled health workforce availability in rural areas remains a challenge in the country.

Table 6: Health worker distribution by county, SARA 2016

Professional Details	Grand Total	Bomi	Bong	Gbarpolu	Grand Bassa	Grand Cape Mount	Grand Gedeh	Grand Kru	Lofa	Margibi	Maryland	Montserrado	Nimba	River Gee	Rivercess	Sinoe
Administrators	1,404	33	93	21	47	48	36	22	108	78	53	638	137	15	30	45
Administrative Support	4,311	129	320	73	184	130	217	103	384	228	131	1,564	525	114	85	124
Clinical Support	3,601	128	220	49	111	99	177	71	269	163	128	1,502	416	83	48	137
EHT	285	9	14	6	15	7	20	2	15	16	7	117	40	4	5	8
Dentist	14	0	0	0	0	0	0	0	2	0	0	11	1	0	0	0
Lab Technician	300	6	35	2	8	6	7	4	12	22	11	139	31	6	3	8
Midwife	927	32	110	25	33	34	35	24	97	40	35	316	79	17	20	30
Registered Nurse	3,077	111	286	55	143	77	77	36	245	162	81	1,270	328	59	51	96
Pharmacist	109	4	5	0	4	3	2	2	5	13	2	54	9	2	3	1
Pharmacy Workers	962	31	78	17	38	38	42	17	75	48	38	377	84	15	23	41
Physician	234	3	20	3	7	4	3	2	9	13	6	128	30	3	1	2
Physician Assistant	518	13	19	9	18	33	23	15	38	24	14	209	49	15	20	19
Public Health Specialist	68	0	1	0	4	2	0	0	0	1	1	56	2	0	0	1
Social Workers	254	5	14	2	3	3	11	2	12	32	6	135	21	2	1	5
Total	16,064	504	1,215	262	615	484	650	300	1,271	840	513	6,516	1,752	335	290	517

Health workforce development

Health workforce development is part of the strategic plan for human resource for health with emphasis on strengthening pre-service, in-service and continuing professional education. The MoH has made significant strides in workforce development to fill the critical training gaps identified during the Ebola crisis. These include improvement in coordination and quality of pre-service, and in-service training. However, recent Health Sector Performance Evaluation (2018) identified the need to revise pre-service training curriculum to provide up-to-date knowledge and skills in elements of health service quality and emergency preparedness and response, including IPC and epidemiological surveillance, to support implementation of global health security agenda and health service quality improvement. The evaluation also highlighted the

need to increase qualified health workers, improve incentives and regulatory frameworks to optimize performance and productivity[22].

There exist post-training/in-service training opportunities for frontline health workers on quality and emergency preparedness. An example is the Field Epidemiology Training Programme for county and district health personnel. Periodic sessions of SimEx and AAR have been organized in the counties. However, there is limited involvement of healthcare facilities and services providers. In-service training in quality has mostly taken the form of workshops on IPC and WASH. However, there is need to emphasize pre-service training and adopt a systematic approach to in-service CPD in quality and emergency preparedness and response in Liberia.

[22] Ministry of Health (2018). Health Sector Performance Evaluation Report (2006-2017). Republic of Liberia

4.2.4. Initiatives to strengthen health services quality and emergency preparedness

In accordance with various health sector policies and plans the MoH, NPHIL and Partners have developed and implemented about ten quality improvement initiatives and around eight emergency-related projects since 2015 to improve quality of health services and emergency preparedness and response as outlined in **Annex IV**.

4.2.5. Integration of quality and preparedness in health services

An important feature of a resilient health system is that it is able to anticipate, detect early, respond to and, therefore, quickly recover from health emergencies, while avoiding interruption of routine services during these emergencies (NHSIP, 2015 – 2021)

The 2015-2021 Health System Resilience Investment Plan seems to be the only document identified during the review that considered health services quality and emergency preparedness as essential for building resilience. However, this integrated approach is yet to be well-developed and embedded within health system and health security initiatives aiming to strengthen health services. Notably, the recent Annual Health Sector Review (2016) and the Health Sector Performance Review (2006-2017) prioritized evaluation of emergency preparedness in tandem with quality of care. However, emergency preparedness aspect was mostly at the administrative levels, indicating the need to increase focus at the health facility level.

Despite significant progress being made in the implementation of the integrated disease surveillance and response (IDSR) post-EVD, 18% of the outbreaks experienced in 2017, and 20% in 2018 have not been promptly detected at county, district, health facility and community level, as evidenced by the 11% community case detection rate in 2017, 22% in 2018 and 26% in quarter one of 2019, far below the national target of 80%[23].

Liberia is a favorable ground for disease outbreaks[24,25]. From January 2016 to August 2019 a total of 223 outbreaks have been detected and responded to in Liberia. These ranged from Lassa fever, Pertusis, Measles, Scabies, Cholera, Chicken pox, Meningococcus to chemical contamination and Pestes outbreaks. The National Reference Laboratory has been in charge of confirming these outbreaks.

However, the capacity of the National Laboratory to diagnose and confirm disease outbreaks, although much improved compared to 2014, is still limited to eight (8) out of the 18 priority diseases. Among the reasons accounting for the limited capacity are: the samples collection and

[23] WHO. (2019). Liberia Country Support Plan, 2019-2020
[24] https://www.cdc.gov/vhf/ebola/outbreaks/2014-west-africa/case-counts.html
[25] Liberia IDSR Epidemiology Bulletin 2017 Epi Week 39 (September 25 – October 1, 2017)

supply chain system is inefficient leading to frequent and prolonged stock-outs of essential commodities; inefficiencies in the health management information system; the limited number and quality of skilled human resources for health; and inadequate funding. These factors further crippled the progress that was made post-EVD outbreak, both for surveillance and management of cases, resulting to limited IHR core capacities as evidenced by the JEE (2016) report.

4.3. Tools and measurement approaches in quality and emergency preparedness

The main tools and measurement approach currently in use for measuring quality of healthcare services include routine data from health facilities, district and county health teams, PBF checklist, SARA/Quality of Care tool, IPC compliance monitoring and Emergency preparedness indicators.

4.3.1. Health Information System (HIS)

The Health Information System (HIS) is used to collect health service data from all health facilities (public and private alike) in the Liberian health system. The HIS data is meant to capture national indicators embedded in the NHPP, Investment Plan for Building a Resilient Health System and other national plans for health services improvement. However, there is need to review and revise indicators to monitor the quality of health services nationwide. This responsibility lies on the HQMU-MoH as stated in the recently published National Health Quality Strategy for Liberia, NHQS (2018). Apart from the HIS the NHPP implementation and the Investment Plan are also expected to be monitored using its proposed M&E framework, annual reviews, and medium-term evaluations. Health service emergency preparedness indicators were not found as part of the indicators in the NHPP M&E framework but are inclusive of indicators listed in the monitoring framework of the Investment Plan.

The reporting system for health statistics through which data is collected from the community and health facility level, synthesized and transmitted through the district and county levels to the national level. This has been further upgraded through the institution of the electronic Joint Integrated Supportive Supervision (eJISS) tool which is currently been implemented in all 15 counties. However, the downstream flow of strategic policy documents and dissemination of information on good practices in quality or emergency seem to be dysfunctional. Data collection is done through a variety of tools among which are checklists, ledgers, reporting forms. Program-specific tools for quality include the maternal and newborn quality of care assessment tool and the IPC Practicum tool for supervision and mentoring. However, there is a need to elaborate an agreed set of indicators for quality and emergency preparedness indicators for the country.

4.3.2. SARA plus Quality of Care

The SARA methodology was developed through a joint World Health Organization (WHO) – United States Agency for International Development (USAID) collaboration.[26] It is built upon previous and current approaches designed to assess service delivery, including the Service Availability Mapping (SAM) tool developed by WHO, and the Service Provision Assessment (SPA) tool developed by ICF International under the USAID-funded MEASURE DHS project (monitoring and evaluation to assess and use results, demographic and health surveys). It draws on best practices and lessons learned from the many countries as well as guidelines and standards developed by WHO technical programs and the work of the International Health Facility Assessment Network (IHFAN). The tool is designed for use at health facility level, to assess and monitor the service availability and readiness of the health sector and to generate evidence to support planning and managing of a health system.

Liberia suffered a 3% drop in the overall readiness index for its healthcare services between 2016-2018. Readiness domain scores, 2018:

- **Basic equipment, 60%**
- **Essential medicines, 35%**
- **Diagnostics, 39%**
- **Standard precaution, 68%**

In Liberia SARA tool was adapted to include selected national indictors for quality of care, hence the name SARA and Quality of Care (SARA$^+$). This assessment has been completed twice in Liberia; 2016 and 2018. The SARA and QoC tool aimed to provide key data and information to enable MOH and partners to fill data gaps and verify the quality of routinely reported data to inform progress and performance in the health sector.[27] It provides a snapshot about the types of health services offered in various health facilities by type (i.e. clinics, health centers and hospitals) and whether those services are provided in line with the NHPP and Investment Plan for Building a Resilient Health System post-Ebola. The domains of service availability and readiness covered in the assessment are; basic amenities, standard precautions, basic equipment, essential medicine and diagnostics. QoC component has only been assessed in hospitals and health centers based on selected priority program (TB, Malaria, Antiretroviral Therapy (ART), and Elimination of Mother -to -Child Transmission (EMTCT) of HIV.

[26] World Health Organization (2015). SARA. An annual monitoring system for service delivery Reference Manual; Version 2.2 Revised July 2015.
[27] Ministry of Health (2018). SARA and Report

Table 7: Quality indicators monitored under priority areas (SARA, 2018)

ART	TB/HIV	Malaria	PMTCT
1)Patients receiving INH preventive treatment by county<br>2)Patients receiving INH preventive treatment according to guidelines<br>3)Patients currently enrolled in TB treatment by county<br>4)Patients' whose TB status was recorded by county<br>5)Patients eligible for Cotrim^{R} according to national standard by county<br>6)Patients' CD4 count documented within the record<br>7)Patients viral load documented<br>8) Patients with ART eligibility criteria documented in the patient record/register prior to beginning ART?	1)Patients eligible for Cotrim^{R} preventive therapy per national guidelines<br>2)HIV positive patient referred/enrolled in ART<br>3)Patients tested for HIV and results recorded by county<br>4)Clinical monitoring checking for symptom and changes documented every visit by health facility<br>5)TB patient household member screened for TB by county<br>6)Patient diagnosed based on 2 of 3 sputum specimens being positive by county	1)Blood test done<br>2)Patients' ACT prescribed dose as per guidelines<br>3)Patients' Malaria blood test prescribed/performed<br>4)Patients clinically diagnosed for Malaria<br>5)Patients whose physical exams results were documented	1)Infants test result performed within 2 months of birth recorded by county<br>2)Infants started on Cotrim^{R} prophylaxis within 2 months of birth by county<br>3)New born receive ARV prophylaxis dose after birth<br>4)HIV Positive Women partners that were tested<br>5)HIV Positive Women that receive Cotrim^{R}<br>6) HIV positive women who did not receive ART after delivery by county.<br>7)Women started on ART during ANC<br>8)Patients' whose physical exams results were documented by county

The review revealed that there are variations between the QoC indicators for 2016 and 2018 in the SARA tool. However, the same priority areas of QoC assessment (table 9 above) were maintained.

There is need to develop a harmonized set of quality indicators which are reflective of the domains of quality (safety, effectiveness, people-centeredness, efficiency, timeliness and equity) to assess quality in the health system of Liberia.

This need is also highlighted in the National Quality Strategy of the Quality Management Unit in the Ministry of Health. Indicators measuring health services preparedness for public health

emergencies also need to be captured in health services assessment tools. It was also found that the application of quality indicators to assess health facilities was inconsistent. For the purpose of improvement, it is important for the national quality indicators to be used consistently (annually) during the SARA and other assessments, in order to track progress and areas which need improvement.

4.3.3. Measuring the outcomes of IPC Interventions Post-Ebola

In addition to the SARA+, the following measurements have been used to evaluate health facilities compliance with IPC standards.

1) Interim Assessment Tool (IAT)

Using the Interim Assessment Tool (IAT), Liberia selected 11 out of 25 global IPC indicators (table 10) to monitor and evaluate adherence of health facilities to standardized IPC practices. The assessment covered all levels of health care delivery (tertiary to primary) measuring the national average compliance of 761 (2016) and 746 (2017) healthcare facilities. Results showed an increase in compliance from 41% in 2016 to 60% in 2017.

Table 8: Summary of IPC measurement tools

Tool	Compliance (year last assessed)
**IAT**	60% (2017)
**WHO,**<br>**-IPCAF**<br>**-IPCAT**	 57% (2018)<br>47%
**Hand hygiene audit**	Active (quarterly)
**HHSAF**	67% (2018)
**VHF isolation & mgt capacity**	62% (2018)
**IPC ring assessment**	Activated

Table 9: Selected IPC indicators for Liberia IAT

No.	IPC Indicators – Liberia
**IPC-1**	Dedicated person (s) for IPC and WASH in place
**IPC-2**	Existence of IPC Committee/Quality Management Team with TOR and written minutes
**IPC -3**	Annual in-service training plan with IPC component share with HCWs, no. of existing HCWs trained in IPC/WASH and recorded
**IPC-4**	Availability of water supply, reliable drinking water point for staff and patients
**IPC-5**	Safe use and management of water containers and quality testing of water at the HCF
**IPC-6**	Adequate, accessible and appropriate sanitation for patients, staff and care givers
**IPC-7**	Functional Hand Hygiene station available at all point of care and good management
**IPC-8**	Waste Management (segregation, label and disposal)
**IPC-9**	Mechanism to track IPC supplies
**IPC-10**	Screening of all patients, staff and visitors and isolation set up
**IPC-11**	Health workers exposure to needle stick injuries

2) **WHO IPC assessment framework (IPCAF) and IPC core components assessment tools (IPCAT)**

Baseline assessment to measure healthcare facilities' IPC compliance was conducted in 2018 using WHO IPCAF and IPCAT. Thirty-two public and private hospitals nation-wide (15 counties) were included in the assessment and obtained average scores of 57% and 47% for IPCAF and IPCAF, respectively. Based on feedback to health facilities, action plans were developed to address identified gaps. There are plans to evaluate implementation of the action plans through follow-up assessments. There are however funding gaps to monitor and supervise implementation of various action plans developed to address gaps across all hospitals in the country.

3) **Hand Hygiene Audit**

National hand hygiene compliance rate among health care workers in hospitals is being monitored quarterly basis (4 times a year). The assessment is facilitated by IPC focal persons from County Health Teams (CHTs) and hospitals with support from WHO and partners. The county level IPC focal persons report to the national IPC coordinators (HQMU-MoH). Data analysis at national and subnational levels helps to inform decisions and areas of focus for interventions. There is need for data management skills for national level staff at the HQMU-MoH to facilitate timely analysis and provision of feedback to subnational levels.

4) **Hand Hygiene Self-Assessment Framework (HHSAF)**

The WHO HHSAF tool is used for national hand hygiene compliance assessment in all counties, with 22 hospitals included in 2017 and 30 hospitals in 2018. The assessment is conducted to identify hand hygiene gaps in health facilities and support improvement in hand hygiene programs, it is to be implemented on a yearly basis in Liberia. Results showed that compliance increased from a 50% baseline to 67% between last quarters of 2017 and 2018 respectively.

5) **VHF-specific IPC compliance**

The Viral Hemorrhagic Fever (VHF) Facility Isolation and Management Capacity Assessment Tool (IMCAT) is used to evaluate the healthcare facility capacity to safely isolate and manage any VHF

An overall VHF isolation and management capacity of 62% gives assurance that on average facilities have the capacity to manage VHF, that the transition of EVD care from Ebola Treatment Units (ETUs) to routine health services has been partially achieved.

---

case (suspected or confirmed). The tool assesses the following parameters of the facility: screening system, facility isolation's infrastructure, staffing, training/SOPs/ job aids, clinical management, IPC/WASH, waste management, supplies, and laboratory. The IMCAT assessments were conducted in 39 HCFs within the 15 counties of which; 26 (67%) were public hospitals and 13 private hospitals (33%), 13 were health centers (33 %) and 25 clinics (64%). The overall national VHF isolation and management capacity was 62%; giving the country an intermediate VHF isolation and management capacity level.

6) IPC ring assessment

As part of emergency preparedness and response, this approach is used to reinforce IPC compliance in a cluster of health facilities within marked high-risk areas for infectious disease outbreak following identification of a case in the area. It also helps to increase health facilities' capacity to identify and isolate suspected cases to ensure staff and patient safety as well as contain the outbreak. When a ring is activated, rapid IPC assessments and immediate interventions are carried out in all health facilities within the ring, through MoH, NPHIL and the support of stakeholders in the response.

4.3.4. Maternal and Newborn Quality of Care Standards

This was recently developed in 2018 by Family Health Division of MoH in collaboration with WHO Family and Reproductive Health (FRH) Unit, Liberia. The tool is designed to assess maternal and newborn quality of care based on WHO standards for improving quality of maternal and newborn care in health facilities. It was piloted in three healthcare facilities in rural Montserrado districts. Nation-wide implementation is yet to be rolled out.

4.3.5. Joint Integrated Supportive Supervision (JISS) Quality Assurance Tool

In 2013, MOH and partners developed an integrated supportive supervision tool to facilitate quarterly supervisions in the various counties to address cross-cutting areas for improvements.[28] Since the development of the tool, some improvements have been reported from counties during follow up process for supportive supervision. This quality clinical assurance tool was revised in 2017 to be used in assessing the quality of all aspects of health services provided at the primary level across the country.[29] The results from the assessment is meant to inform the MoH on the status of health services at individual health facilities and existing gaps in service delivery which require improvements. The tool was recently used to introduce the concept of Quality Management Teams at the County, District, health facility levels, and quality assurance (including IPC/WASH), in addition to the technical areas.

[28] Ministry of Health. (2013). EPHS accreditation final report
[29] Ministry of Health. (2017). Joint Integrated Supportive Supervision Tool, Revised 2017.

The JISS quality assurance orientation has been carried out in 3 health regions of the country. The tool was initially implemented in 3 counties using the paper-based, which is now been automated and linked to DHIS2 pilot project in Bong County. There are however funding gaps to scale up the tool to all primary healthcare facilities across the 15 counties, and the need for improved feedback and follow up process from disease-specific programs to support counties in addressing gaps identified has been stressed to improve health service delivery at the facility level.

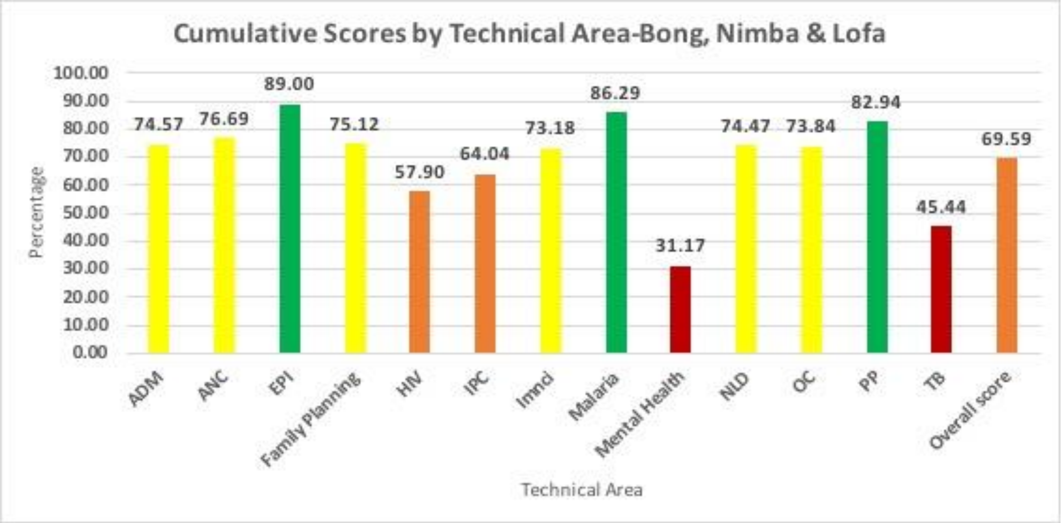

Legend

Score	% Achievement	Interpretation	Color Code
0 - 14	0- 49%	Inadequate	Red
15 - 20	50 – 69%	Basic	Amber
21 - 23	70 - 79%	Intermediate	Yellow
24 - 30	80% and above	Adequate	Green

Figure 6: JISS national baseline scores

4.3.6. Health Facility Accreditation

The Essential Package of Health Services (EPHS) accreditation process was instituted by the then Ministry of Health and Social Welfare in 2012. It was intended to help health facilities; CHTs, partners and the MOH identify shortfalls and improve the services they are providing. The last EPHS Assessment was conducted in 2013 at all functioning government health facilities and some Faith-Based Organizations (FBOs) receiving support from the government under the FARA project.

The accreditation tool used was made of questions targeting 12 technical areas in health care[30]:

1. Quality Assurance (ANC, NLD, OC, PP, FP, MAL, TB, HIV/AIDS, EPI, IMNCI and Mental Health)
2. Policy and Standards (SOPs, guidelines etc)
3. Human Resources
4. Drugs, supplies and equipment
5. Pharmacy, Dispensary and Store Room
6. Laboratory
7. Infrastructure
8. Infection Prevention and Control
9. Medical Record Management
10. Referrals and Community Health Outreach
11. Mental Health
12. Prison Health

Scoring

The last health facility accreditation assessed a total of 396 facilities (341 clinics, 24 health centers and 31 hospitals) including 15 prison health facilities in the 15 counties in Liberia. It entailed the grading of health facilities according to the scale in table 11 based on a set of indicators outlined above. A cut off score of 70% was used in 2013 assessment to determine the number of health facilities providing the EPHS in Liberia. Only 14% (55) of assessed facilities met this cut off score. However, a new cut off score of 60% was adopted after evaluating the 2012 and 2013 assessments.

Table 10: Grading scale used in HF accreditation

Grade	Score
**Gold (Two star)**	90 – 100%
**Silver (One star)**	80 – 89.9%
**Bronze (Half star)**	70 – 79.9%
**No Award**	<70%

Although the accreditation system was not meant to measure QoC, it provided information on facility performance in meeting basic standards which are essential for quality health services, based on the Basic Package for Health Services (2008-2011)[31].

Table 13 below presents a summary of the performance of the three categories of health facilities assessed during the 2013 accreditation process.

[30] 2013 EPHS Accreditation Final Report
[31] Cleveland, E. C., Dahn, B. T., Lincoln, T. M., Safer, M., Podesta, M., & Bradley, E. (2011). Introducing health facility accreditation in Liberia. *Global Public Health*, *6*(3), 271-282.

Table 11: National average accreditation scores by facility types in 2013

Level	Clinic	HC	Hospital	Overall	Percentage
**Two stars (>90%)**	0	0	0	0	**0%**
**One star (>80%)**	8	2	0	10	**3%**
**Half star (>70%)**	34	2	5	41	**10%**
**<70%**	299	20	26	345	**87%**
Total	**341**	**24**	**31**	**396**	**100%**

From the last accreditation carried out, the average national score was 54% and the highest performant county was Grand Gedeh, while Grand Kru performed the poorest. Concerning the healthcare area, TB services had the lowest score (25%). However, none of the areas of healthcare attained a quality score above 50% as demonstrated in the figure below.

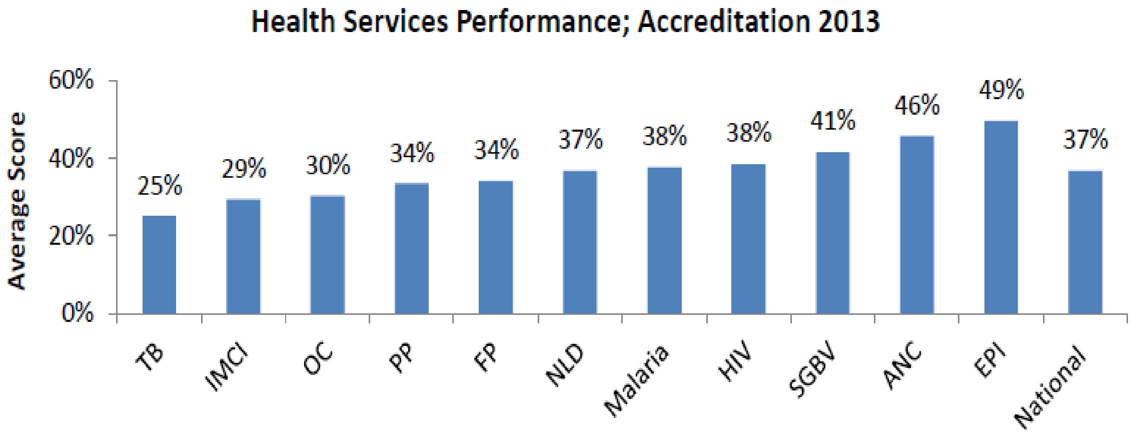

Figure 7: Average health service score by clinical area

In line with the upgrade of BPHS (2008) to EPHS (2011), the accreditation standards are currently being revised by the Liberian Medical and Dental Councils (LMDC), MoH and partners to reflect the EPHS (2011) and include quality improvement indicators. However, technical and financial support are needed to finalize and roll out the new accreditation standards[32]**.** LMDC is responsible for leading the regular accreditation process of health facilities in collaboration with MoH and partners.

4.3.7. IHR Measurements for Emergency Preparedness

State Party Self-Assessment Annual Reporting (SPAR)

The aim of this assessment is to evaluate countries capacity to prevent, detect and respond effectively to public health threats, based on IHR (2005) and National Action Plan for Health Security (2018). The country continues to report annually on the IHR (2005) core capacities in the

[32] Ministry of Health (2015). Investment plan for building a resilient health system in Liberia.

SPAR tool, since 2015. Each of the 13 core capacities has a number of indicators graded into 5 levels of performance, capturing various elements of emergency response at national and subnational levels.

This tool has recently been revised by WHO to include a health service capacity (C9) namely;

- Case management capacity for IHR related hazards (C9.1)
- IPC and chemical and radiation waste decontamination capacity (C9.2)
- Access to essential health services (C9.3)

Liberia is one of the countries which reported using the revised IHR SPAR tool in 2018, with overall score of 33% in the health service provision capacity (C9). The regional and global averages (39% and 59% respectively, were also low. Scores in health services provision capacity indicators were; Case management (20%), IPC/chemical and radiation decontamination (40%), and access to essential health services (40%).

Joint External Evaluation (JEE)

The Ministry of Health in collaboration with relevant line ministries, agencies and development partners conducted the first IHR-JEE in September 2016 which revealed strengths, gaps and made recommendations which informed the development of the NAPHS (2018). Some strengths reported in the 2016 JEE included; (1) strong political will for developing IHR capacities using multi-sectoral health approach, strong partnerships at subnational, national, regional and global levels; (2) significant progress after the EVD-crisis in all domains of human and public health (including robust surveillance system at national and subnational levels in the human health sector); (3) establishment of Field Epidemiology Training Program (FETP), emergency operations centers (EOCs); (4) Incident Management System (IMS) at national and intermediate levels; (5) strong foundation in IPC practices in health facilities through the safe and quality health services (SQS) training program. [33]

However, the 2016 JEE noted that major weaknesses continue to exist including, (a) weak national laboratory network, quality and management system; (b) shortage of a multidisciplinary workforce to implement the IHR core capacities requirements; (c) absence of a multi-hazard National Public Health Emergency Preparedness and Response Plan; (d) poor multidisciplinary coordination and communication mechanism for IHR implementation in the animal and environmental health sectors; (e) poor integration of National public health EPRP with the points of entry (POEs) emergency plans; (f) lack of AMR detection capacity using One Health approach and (g) insufficient funding for IHR core capacity building from domestic and international sources.

[33] Liberia 2016 International Health Regulation Join External Evaluation Report

The next JEE is planned for 2021, while ongoing implementation of NAPHS is being monitored using its monitoring and evaluation framework. The National Public Health Institute of Liberia (NPHIL) is the lead agency for developing NAPHS and joint supervision involving MoH, One Health Secretariat and partners. The JEE is to be conducted to monitor progress of implementation of the NAPHS (2018). Most indicators included in NAPHS are being monitored through the M&E plan of MoH, NPHIL strategic plan, GHSA, and other program specific plans [34].

Simulation exercise (SimEx)

Simulation exercise can be an effective method of testing the capacity of health systems to deliver quality health services in response to emergencies. The NAPHS proposes that Liberia conducts at least one SimEx annually at national and county levels to test the functionality and to validate the functional capacities of the IHR (2005). The findings from the SimEx are expected to provide an indication on the level of capacities across 19 technical areas[35]. Both the IHR-JEE (2016) and NAPHS (2018), emphasize the needs for the country to undertake integrated capacity development (training, simulation exercises, experience sharing) and encouraged inter-sectoral collaboration through inclusive SimEx at both national and county levels to test the emergency preparedness plans.

In November 2018, a two-days field- based SimEx was carried out by NPHIL in collaboration with the MOH and partners in Margibi and Montserrado Counties with the objective of assessing knowledge and capacity at the port of entries (PoEs) related to EVD preparedness and response.

Findings showed that PoE health staff lacked knowledge and skills on priority diseases case definition, initiation of contacts tracing, lack of logistics (including lack of fuel for ambulance, IPC supplies, POE SOPs), poor waste management, and staff did not use risk appropriate PPEs.

Another SimEx conducted in Liberia in 2018 was the global EOC SimEx, which was a three-days, exercise featuring over 40 countries, 30 participants from key stakeholders (NPHIL, MoH, MoA, EPA, NRL and partners CDC, GIZ, ACCEL, Africa CDC) with support from WHO. Findings helped to improve the country's readiness to hazards of epidemic potential[36].

[34] NAPHS (2018)
[35] Ibid
[36] WHO. 2018. WHO Liberia Country Office Report

Despite the usefulness of the various SimEx in testing several areas of health system preparedness for public health emergencies and resilience, reviews and reports on simulation packages implemented in Liberia found that there are significant areas needing improvement.

Some of the areas for improvement are; testing health sector capacity to maintain routine health services during emergency response, integration of business continuity plans with emergency preparedness plans, inclusion of primary level health facilities and using inter-sectoral approach. These agree with a recent WHO global mapping of simulation exercises materials.[37]

Based on recent national reports, issues of staff retention have created setbacks in PoE and RRT capacity building in Liberia[38]. Approximately 60% of PoE staff trained in 2016 have either left or changed positions making them no longer part of the original cohort of trained and tested county, district RRTs[39]. Surge capacity is provided by the 15 County RRTs which were established and trained in 2016, but some of these gains have been lost due to poor staff retention[40]. Urgent attention is needed to strengthen the PoEs and RRTs as part of public health emergency preparedness.

After Action Review (AAR)

AAR helps to review actions taken to respond to an emergency or outbreak. It provides an opportunity to identify what worked well, challenges, lessons learned and best practices. As recommended by IHR (2005) Liberia has conducted national and county level AARs following response to public health events in the country. In 2018, meningitis AAR was held in Lofa County with 36 participants. Lassa fever AAR held in Montserrado county brought together about 57 participants.[41]

4.3.8. Performance of Veterinary Services (PVS) Gap Analysis

This assessment in human and animal health by relevant agencies is meant to assess implementation of the Liberia NAPHS as part of emergency preparedness initiatives. In Liberia, few IHR PVS measurements have been undertaken including, but not limited to, PVS evaluation mission conducted in January 2013. This PVS evaluation was conducted with support of the World Organization for Animal Health (OIE). The key findings from this assessment were: insufficient staff at all levels to conduct animal health activities effectively and efficiently; staff do not benefit from continuous training; most services have inadequate facilities at the local level, particularly at border crossings and the country has only one central veterinary laboratory. The findings also showed that other laboratories are not functional due to lack of equipment and operating budget; the diagnosis of diseases is made on the basis of the observation of clinical symptoms;

[37]World Health Organization. (2019). Global mapping of simulation exercise materials: a health system review, technical report.
[38] Ministry of Health. (2018). Simulation exercice report
[39] Ministry of Health. (2018). Simulation exercise report
[40] Liberia 2016 International Health Regulation Join External Evaluation Report
[41] Ibid

there is no surveillance program (passive or active) due to the lack of technical skills and diagnostic capacities in laboratories, limited veterinary services capacity to prevent and control diseases of animal origin; border controls remain very weak and the risk of disease introduction through illegal cross-border movements is high; state financing of veterinary services is insufficient and inadequate in relation to the country's needs. [42]

The assessment proffered some recommendations, such as strengthening the following services: veterinary services, surveillance, initial and continuing training of veterinarians, allocating adequate budget to veterinary services, updating veterinary and health laws and regulations, improving infrastructure and developing basic diagnostic capacities, etc[43]. A IHR-PVS (OIE, FAO, and WHO) national bridging workshop held 2018 in Buchanan, provided an opportunity to the human, animal and environmental health services to build on the reviews of performance, gaps and discussions for improvement conducted in their respective sectors, and to explore options for improved coordination between the sectors, to jointly strengthen their preparedness for, and control of, the spread of zoonotic diseases. Priority areas identified for strengthening included coordination at local level, laboratory capacity, surveillance and field investigation, and risk communication and emergency response capacity.

[42] National Bridging Workshop on the IHR and the OIE-PVS Pathway, 2018
[43] Bastiaensen, P. et al. (2016). OIE-IHR-PVS gaps analysis mission report, Republic of Liberia

Highlights of monitoring and measurement of health care quality and emergency preparedness

- There is currently a lack of integrated approach to measure quality and preparedness aspects in health services. SPAR C9 can be considered as example of good practice which would require strong input of health service delivery professionals from national to district levels for adequate representation.
- The efforts of quality measurements are centered around EVD based efforts and mostly related to IPC and to an extent MCH. The long-term sustainability for this focused effort is uncertain.
- MoH has successfully applied accreditation tool to ascertain the performance of healthcare facilities but the tool is limited its focus to measure quality aspects of health services.
- Joint supportive supervision has been identified to link up with DHIS2 but funding constraint is limiting their roll out and sustenance.
- Current tools and protocols are largely based on IHR (2005) MEF except the PVS tool. Country successfully participated in reporting for C9 Health Service Provision capacity and the overall score of 33%. JEE tool applied successfully in 2016 and the next round is due in 2021 – however the tool is limited in the scope to ascertain the preparedness of health services.
- HCWs trained in IPC, WASH in the context of EVD and post-EVD campaign have not stayed in their respective role and succession of skills is therefore uncertain due to long term financial outlook.
- Limited veterinary services capacity to prevent and control diseases of animal origin; border controls remain very weak and the risk of disease introduction through unregulated cross-border movements is high. Routine linkages between public health and animal health on joint preparedness, reporting, surveillance and response needs improvement, despite some progress being made.

4.4. Authorities, stakeholders and their role in quality and emergency preparedness

The various authorities and stakeholders involve in quality and emergency were identified during a preliminary consultation that formed part of the broader situational assessment of the health system. The stakeholders, general roles and specific involvement in QoC and emergency preparedness are summarized in Annex VI.

The 2014–2015 EVD outbreak in Liberia exposed the fragility of the health system and its limited capacity to respond to outbreaks while simultaneously providing quality routine health services. As part of the lessons learnt, Liberia has prioritized quality of care in routine health service delivery and building resilience to prepare and respond adequately to subsequent outbreaks. Consequently, the government through the MOH created the Healthcare Quality Management Unit (HQMU) and finalized the National Quality Health Strategy, 2017-2021. The elaboration of these quality instruments is aligned with the goals of the National Health Policy and Plan (2011-2021) and the Investment Plan for Building Resilience in the Health System (2015-2021).

In 2017, the government of Liberia established the National Public Health Institute of Liberia (NPHIL)[44] with support of partners to provide expert advice on the causes of death and disability to the MOH, decision makers, County Health Teams (CHTs) and the public, to implement epidemic prevention and control measures. NPHIL collaborates with the MOH and strengthens the existing infection prevention and control efforts, laboratories, surveillance, infectious disease control, public health capacity building, response to outbreaks, and monitoring of diseases with epidemic potential.

As earlier highlighted, Liberia conducted a Service Availability and Readiness Assessment (SARA) and Quality of Care assessment and also completed the JEE in 2016. From the JEE findings, the National Action for Health Services (NAPHS) was developed. A recent SARA assessment was carried out in 2018. There are disease-specific plans such as the Antimicrobial Resistance (AMR), Emergency Preparedness and Response plans (EPR). Also, there is a 'Global Health Security Roadmap for Liberia' that elaborates the emergency preparedness and response plan, including hospital preparedness.

In 2008 the Ministry of Health and Social Welfare (MOHSW) established a National Reference Laboratory to improve the diagnoses and confirmation of diseases, especially those with epidemic potential. Five regional laboratories have been earmarked for construction to decongest the central laboratory. However, only Phebe and Tappita laboratories are currently been developed.

[44] Investment Plan for Building a resilient health system (2015-2021)

There exist many guidelines, and SOPs on various areas of care produced and disseminated by the MOH with the support of partners, with the goal of improving the quality of care and emergency response. Such include; the National IPC policy, WASH guidelines, IPC guidelines, IDSR guidelines, and disease-specific contingency plans, manuals/protocols like HIV and TB.

Highlights of the extent of stakeholders and institutions in Liberia

- The institutional and legislative framework work in the country is robust to support an integrated approach to health systems strengthening. The set-up of NPHIL and Quality Unit in MoH post-EVD are a laudable initiative to promote public health protection alongside MoH and Disaster Management Authority.
- There is also clear reference to the presence of subnational and facility levels health infrastructure including community health workers which would enable operationalization of national policy for resilient health services to contain the event fast and effectively.
- There is also good presence of NGOs and donors operating in the country including growing private sectors; however, their inclusion in national emergency preparedness and quality health services is unclear.
- Professional and academic sectors are also identified in different functions which can be potential resource to promote health systems resilience by building health workers capacity (pre-service and in-service perspectives)
- There is robust partnership between MoH, NPHIL and WHO on health emergencies, health systems and other health/disease programs which can be harnessed for integrated approach to health systems strengthening.

5. Findings from the site assessment

In addition to desk-based situational assessment, visits to specific health facilities and offices were conducted to review their status and feasibility to partake in the project. This was facilitated through a detailed site selection matrix and an assessment. This section presents a synthesis from the three key activities carried out during the situational assessment (desk review, stakeholders' engagement and the field visit). However, we start by highlighting the major findings from the health facility visit.

The site assessment was carried out in 19 health facilities (10 clinics, 5 hospitals and 4 health centers) in 10 health districts and 3 health counties (Bong, Lofa and Cape Mount) as represented in figure 13. Of these, 15 (79%) of the health facilities were public. Two of the assessed hospitals were from Bong and Lofa County, respectively.

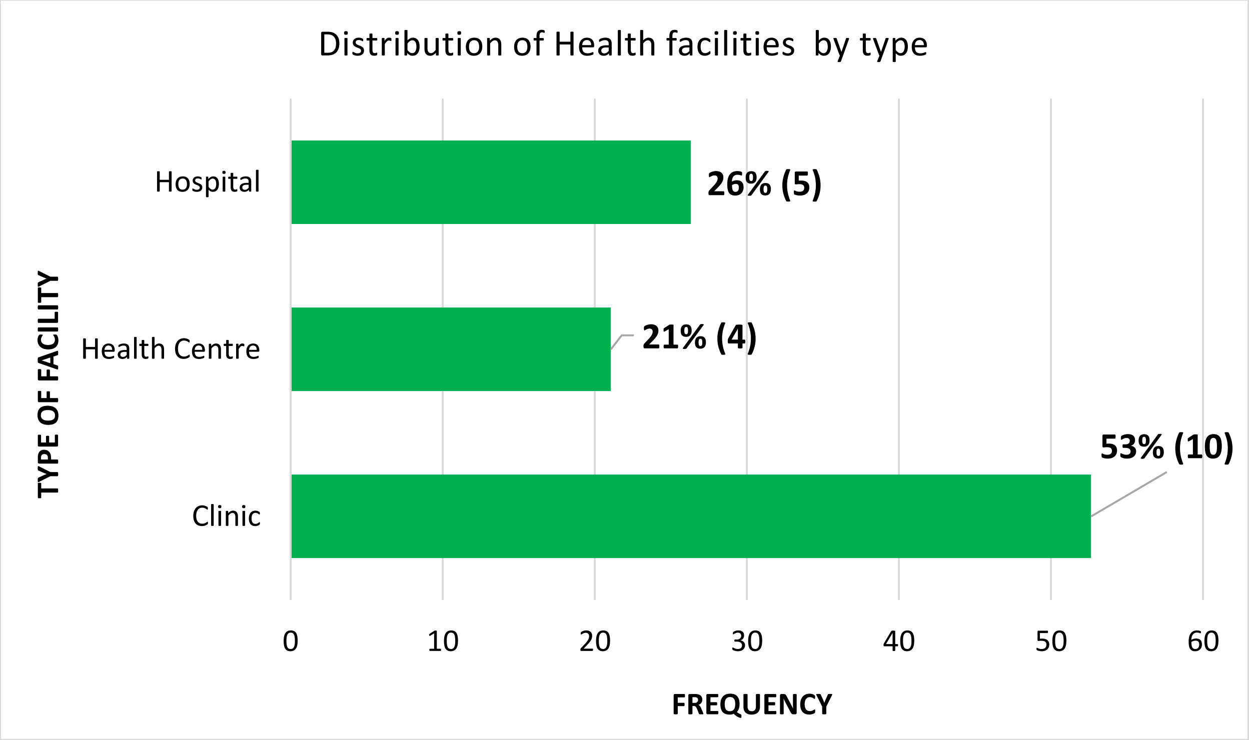

Figure 8: Distribution of health facilities assessed by type

5.1. Distribution of health facilities

Majority of the health facilities (37%) assessed were from Lofa County, especially in the Voinjama district as shown in the figure below. Lofa County is strategically located as it is a crossroad for Liberia, Sierra Leone and Guinea. Six health facilities were each selected from Bong and Grand Cape Mount counties.

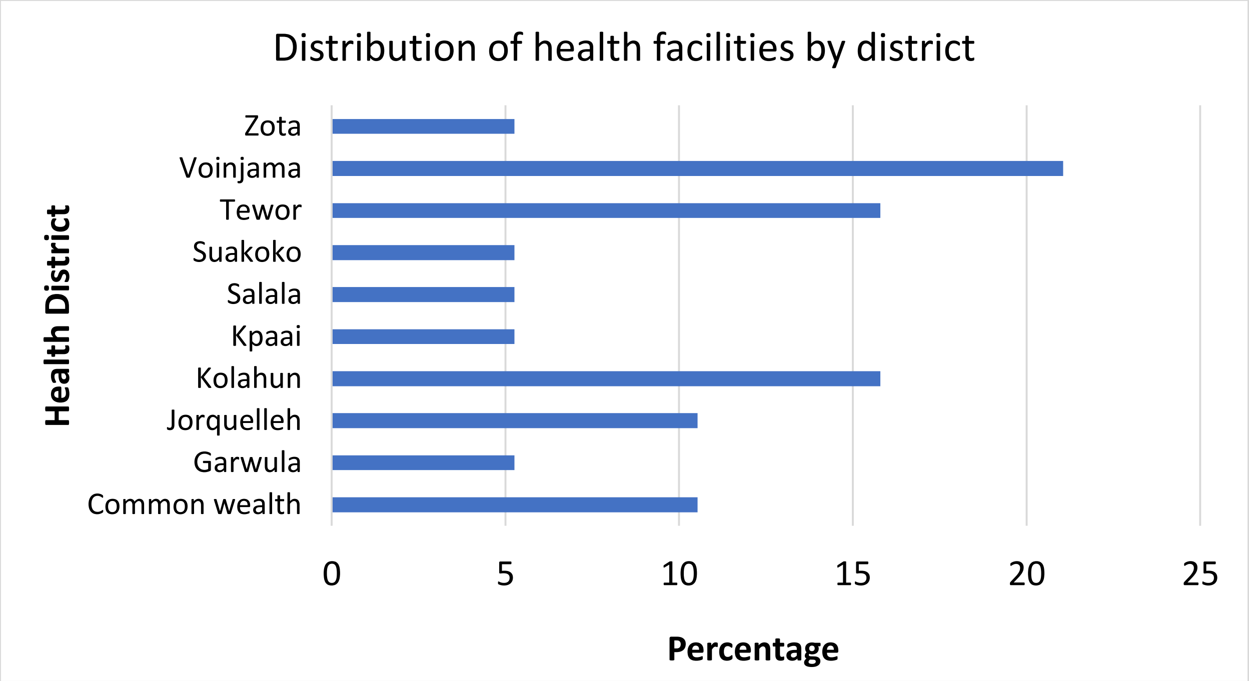

Figure 9: Distribution of health facilities by district

5.2. Health worker distribution in selected health facilities

The 19 health facilities in this pilot served a population of about 294,151 inhabitants, of which majority (49%) of them were found in Bong county. The catchment population for the 19 facilities are being served by 784 health workers; registered nurses making the bulk (26%) of the clinical workforce. Figure 15 shows that 550 (70%) of the entire health workforce from the 19 health facilities was found in the Bong county, and mainly registered nurses and midwives.

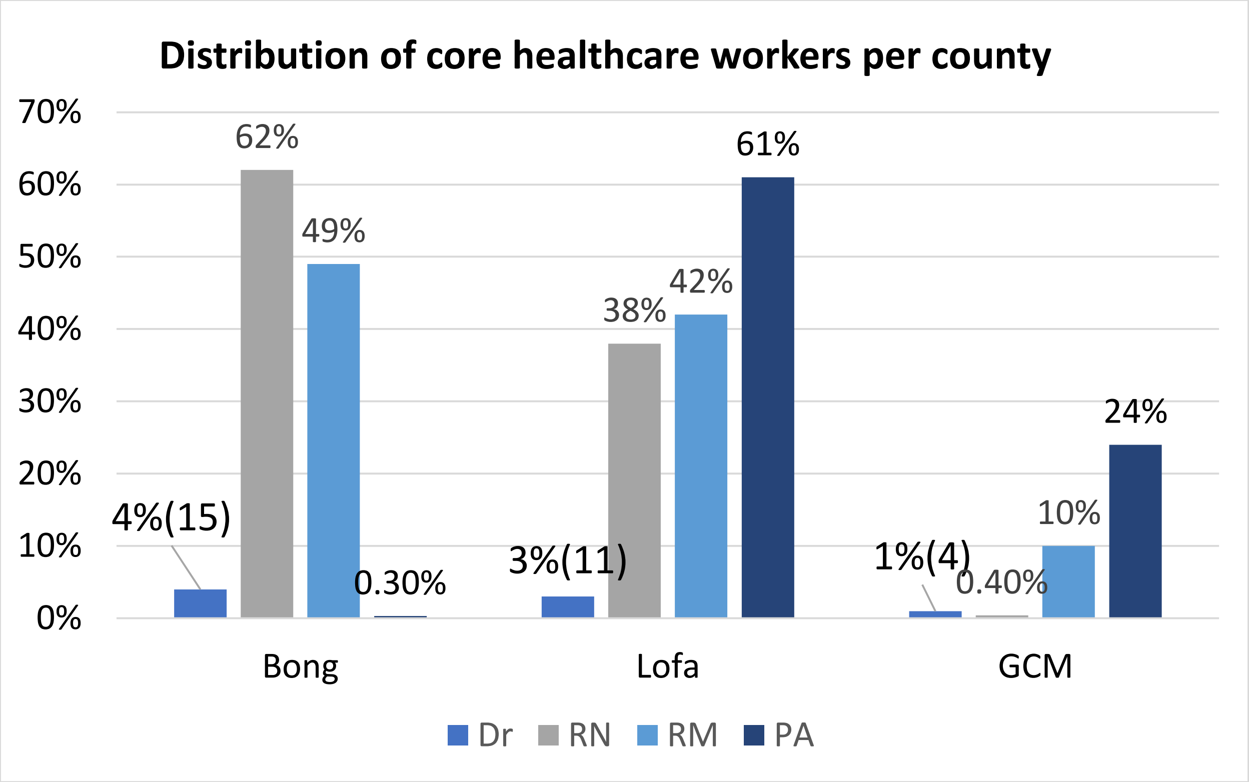

Dr= Medical Doctors, RN= Registered nurses, RM= Registered midwives, PA= Physician assistants

Figure 10: Core workforce distribution per county

Among many factors, the easy geographical accessibility, better health infrastructure and social amenities attract more of the skilled workforce to work in this county. Conversely, physician assistants who are trained to fill the critical shortage of doctors are predominantly found in Lofa and Cape Mount counties where Medical Doctors are few. Table 15 represents a detail analysis of the health workforce recorded during the field visit.

Table 12: Distribution of health workers cadres by health facility and county

County	District	Health facility	Type	Ownership	Catchment pop	*Doctors	*Physician Ass	Mental Health clinician	Lab personnel	*Registered midwives	*Registered nurses	Pharmacist	Dispenser	Comm. Health workers (CHAs, CHVs, TTMS)	Nurse anesthetists	Nurse Assistants	Total per facility
Bong	Jorquelleh	Baptist clinic	Clinic	PNFP	4,337	0	2	1	5	2	2	0	2	0	1	0	**15**
		CB Dunbar	Hospital	Public	53,714	4	3	2	5	47	38	2	8	0	20	4	**133**
	Kpaai	Palala	Clinic	Public	15,884	0	0	1	1	3	3	0	1	75	0	0	**84**
	Suakoko	Phebe	Hospital	PNFP	35,000	8	1	1	4	22	82	1	9	0	69	6	**203**
	Zota	Belefanai	Clinic	Public	7,385	0	0	0	1	2	4	0	1	76	0	0	**84**
	Salala	Salala	Clinic	Public	27,542	0	0	0	1	2	3	0	1	23	1	0	**31**
GCM	Garwula	Sinje HC	Health Center	Public	6,723	1	3	0	4	9	9	1	3	8	0	1	**39**
	Common wealth	Madina	Clinic	Public	4,408	0	0	0	0	0	2	0	1	13	0	0	**16**
		St. Timothy	Hospital	Public	2,075	1	3	1	3	4	15	1	5	0	4	2	**39**
	Tewor	Tienni	Clinic	Public	5,289	0	1	0	1	0	2	0	1	0	0	0	**5**
		Bo Water	Clinic	Public	2,752	0	0	1	0	0	3	0	1	8	0	0	**13**
		Devos	Health Center	PNFP	1,431	1	2	0	2	1	2	0	1	0	2	0	**11**
Lofa	Voinjama	Free Pent	Health Center	PNFP	4,878	0	0	0	3	2	6	0	1	0	0	0	**12**
		Bondi	Clinic	Public	3,614	0	0	0	0	1	1	0	1	13	0	0	**16**
		Tellewoyan	Hospital	Public	77,336	4	3	2	9	15	9	1	10	17	0	1	**71**
		Barkedu	Clinic	Public	12,364	0	0	1	1	2	4	0	1	55	0	0	**64**
	Kolahun	Korworhun	Clinic	Public	6,473	0	0	2	0	1	2	0	1	42	0	0	**48**
		Kolahun	Hospital	Public	17,000	2	4	1	6	8	12	1	4	1	0	2	**41**
		Bolahun	Health Center	Public	5,946	0	1	2	1	3	3	0	1	20	0	0	**31**
Total					**294,151**	**21**	**23**	**15**	**47**	**124**	**202**	**7**	**53**	**179**	**97**	**16**	**784**

The findings from the field visit on quality and emergency preparedness and response will be presented according to the WHO six domains of interventions for quality[45], which has also been used as a lens to examine the possible levers, barriers and opportunities for the integration of quality and emergency response in routine healthcare. The domains include: 1) leadership, 2) information, 3) patient & population engagement, 4) regulation and standards, 5) organizational capacity and 6) models of integration.

5.3. Leadership (policies/plans)

Leadership was assessed by making inquiry on the presence of a functional team and focal person in charge of quality and emergency response at the health facility.

For the presence of a quality management team (QMT), more than half (68%) of the health facilities admitted to having a 'functional' QMT. Meanwhile, 10 (53%) of the facilities reported having focal persons for QI, of which a TOR was found in 9 of the health facilities pertaining to the IPC focal person. However, it was realized that these figures were overstated as the QMT, where present, was mostly implied with no organizational framework. Besides, the QI focal person was likened to the IPC focal person in all the health facilities assessed.

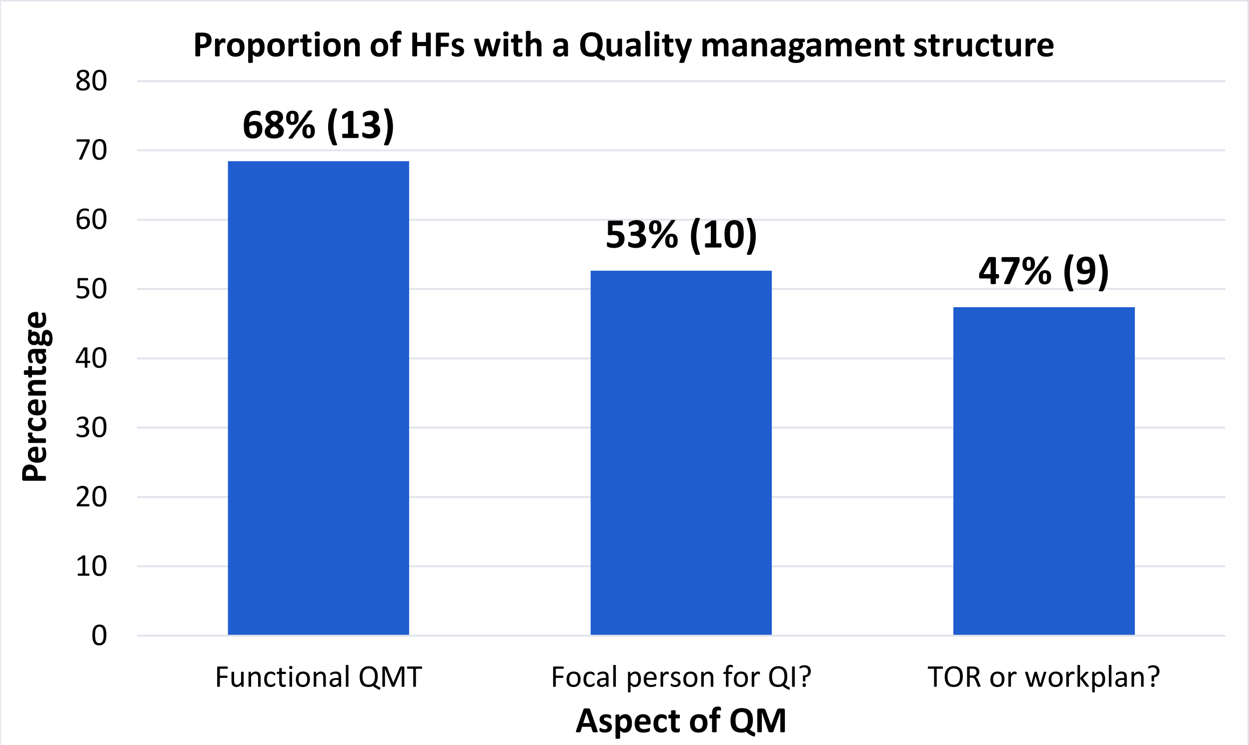

Figure 11: Proportion of facilities with a leadership structure for quality

[45] WHO. Quality of Care: a process for making strategic choices in health systems, 2006

On the contrary, very few health facilities reported having a team (21%) or focal person (37%) in charge of emergency preparedness and response activities in the health facilities as shown in the figure 17 below. None of the health facilities had an emergency preparedness and response plan in place.

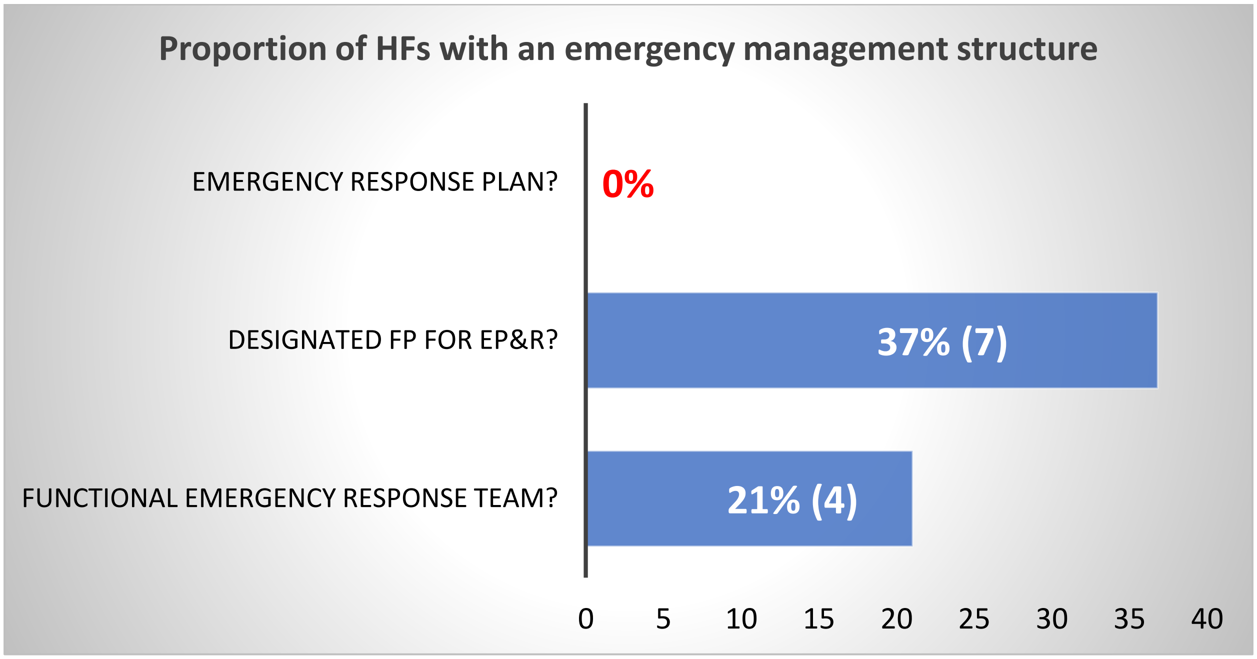

Figure 12: Proportion of facilities with a leadership structure in emergency

Another aspect of leadership that was assessed was the availability of funding for quality and emergency. Less than half of the health facilities could identify an ongoing project funding QI (47%) or emergency preparedness (37%) or both (11%).

The following partners were reported to fund projects focused on QI at the health facility level: CHAI, ACCEL, UNFPA, and PACS. Meanwhile, PACS was reported to support community workers in conducting disease surveillance in the communities. Three respondents (Palala clinic, C.B Dunbar Hospital, Tellewoyan Memorial Hospital) in Bong and Lofa Counties identified Performance-Based Financing (PBF) and Fixed Amount Reimbursement Activity (FARA) as projects integrating quality and emergency response in the health facilities.

Interviews with the county and district health teams revealed that no budget was specifically allocated for quality improvement or emergency response at the operational level of the health system.

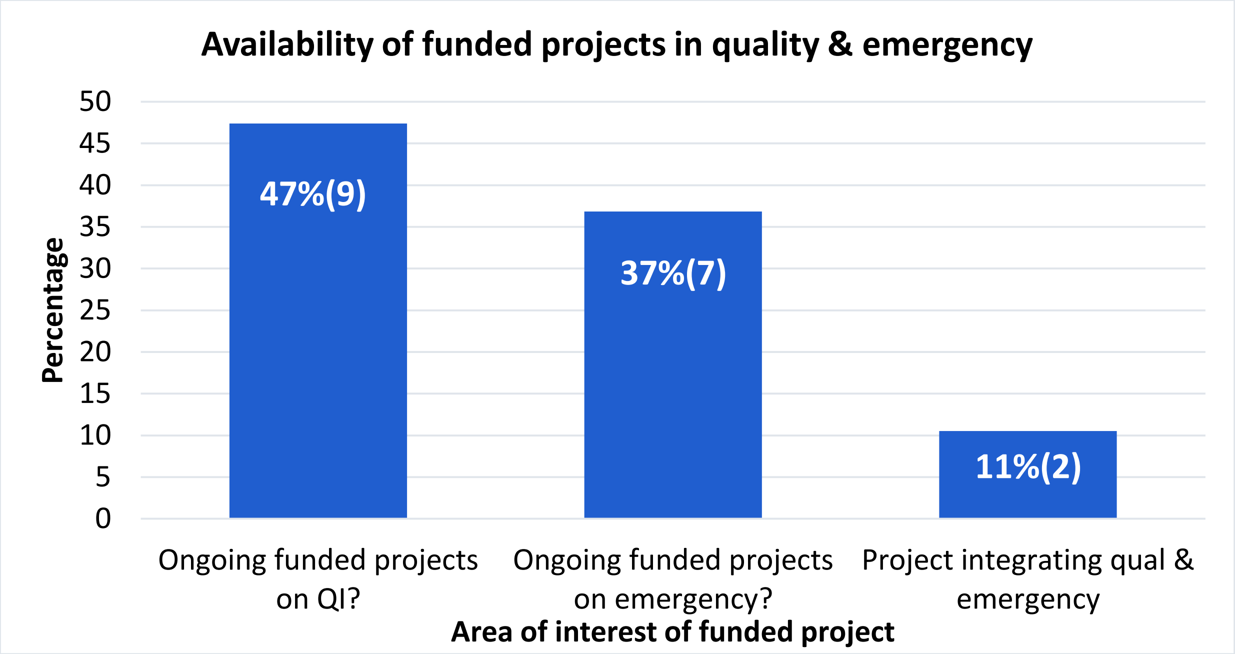

Figure 13: Funding for quality and emergency at the facility level

5.4. Information (measurement tools)

The availability of tools to measure quality and emergency preparedness in the health facilities was also assessed. Besides, the practice of feeding back information to the facilities for improvement was also analyzed.

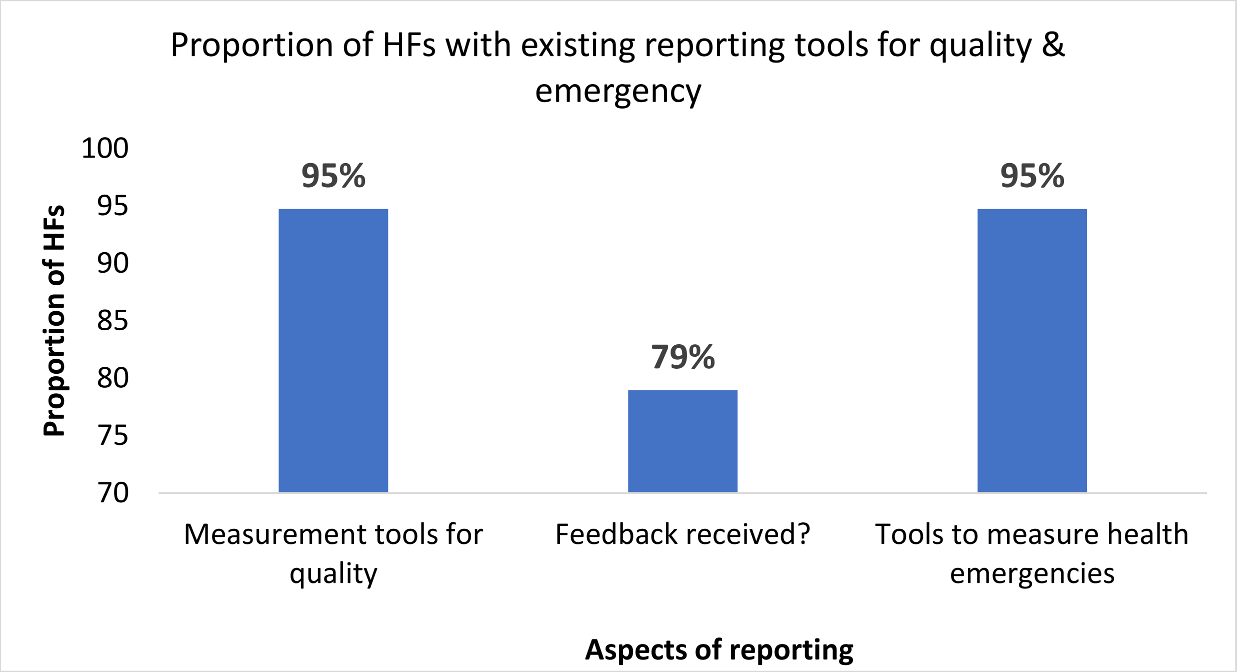

Figure 14: Availability of reporting tools and feedback on quality and emergency

In 18 health facilities (95%), the availability of measurement/reporting tools for quality and emergency was reported. These include checklist, data reporting forms, ledgers and registers. Besides, there is an electronic tool for Joint Integrated Supportive Supervision (JISS) which is used by the district and county supervision teams. It was found that most of the reporting tools were used to collect a wide range of data from the facility without differentiating quality from emergency. However, there are no explicit indicators to capture QI practices within the health facilities.

Despite the scale of reporting from the facilities, four of the respondents denied receiving any feedback from the district or county health teams to which data is transmitted. On the contrary, all of the District Health Officer (DHOs) reported that feedback is made to all the health facilities immediately after supervision using the JISS tool.

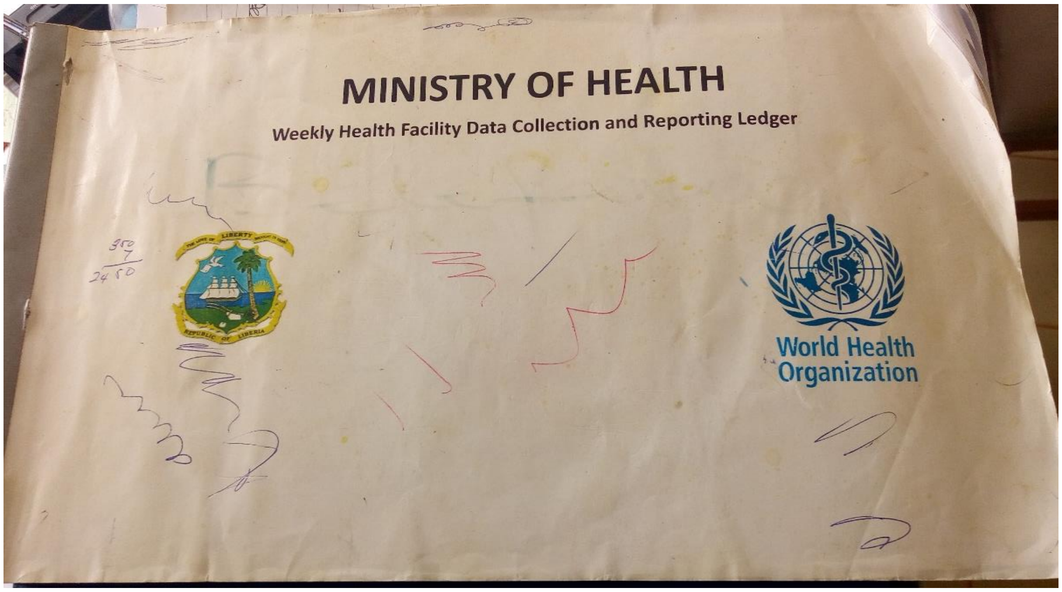

Figure 15: Data collection ledger used at the health facility level

5.5. Patient and population engagement (stakeholders)

The involvement of the communities in health service planning and decision making is important to ensure patient satisfaction, sense of ownership of health facility, collaboration in the notification and respond to epidemics in the communities. Majority of the health facilities (74%) assessed reported that their population is represented at planning meetings by elected representatives of the development committee and village chiefs, as shown below.

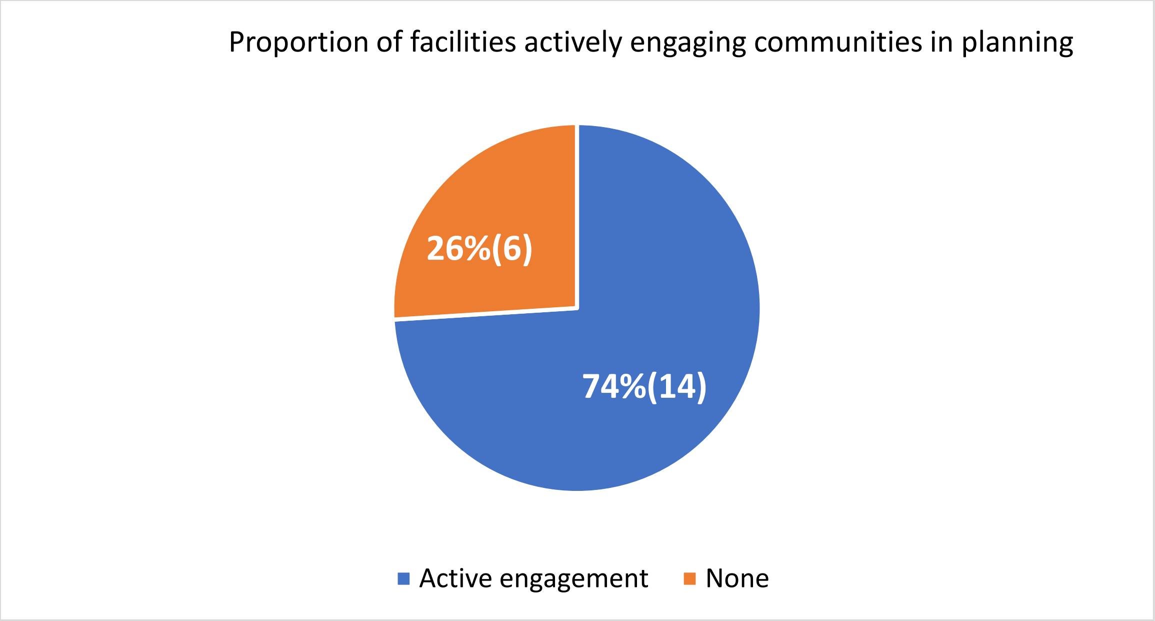

Figure 16: Representation of health facilities per active engagement of their population in planning

5.6. Regulation and standards

Aspects of regulation and standardization assessed during the field visit included; accreditation, laboratory capacity, and infrastructure. Most of the facilities (84%) reported having basic infrastructure (building, water, electricity) to provide quality healthcare. Less than half were found to have the laboratory capacity to diagnose basic pathologies in the community. The lack of capacity was mostly linked to the absence of basic supplies like laboratory reagents and other consumables. On the contrary, 12 (63%) health facilities reported to have adequate laboratory capacity to diagnose some of the diseases under surveillance in the health facility and community, as shown in figure 19. However, this capacity was referring mostly to the ability to send samples for analysis at the reference laboratories (Phebe Hospital or National Reference Laboratories) and not in the health facility itself.

Concerning health facility accreditation, 10 of the health facilities assessed (53%) declared having an accreditation from the MOH, although none presented a certificate of accreditation. Two of the respondents were not certain of the accreditation status of their health facility (figure 20). From discussions with the accreditation board and MOH, the last accreditation was carried out in 2013. This means that none of the health facilities is currently accredited to provide the EPHS.

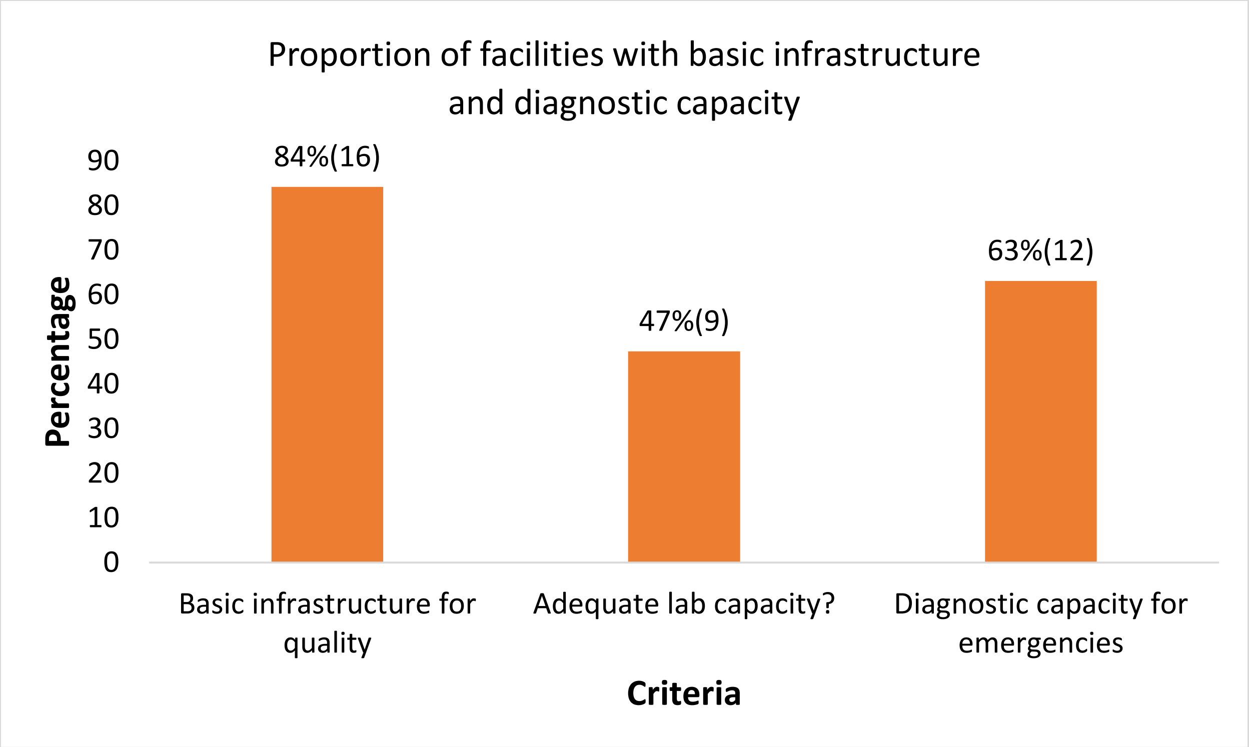

Figure 17: Infrastructural and diagnostic capacity among health facilities assessed

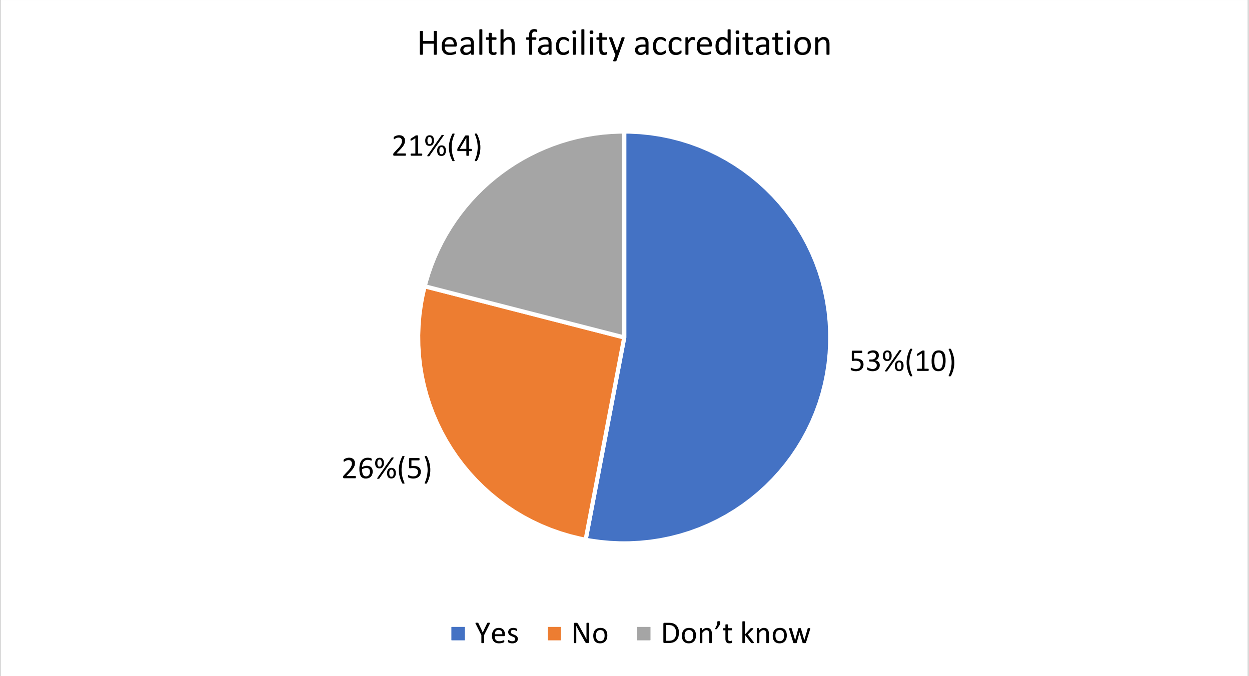

Figure 18: Declared status of accreditation of health facilities

5.7. Organizational capacity in health facilities

This domain was assessed by making inquiry about training of health personnel, availability of strategic documents, capacity building activities on quality and emergency preparedness and response.

Out of the 19 facilities visited, just 4 (21%) had health personnel who have undergone some form of continuous professional development in quality. However, from the majority of facilities in which personnel did not follow any CPD, 12 (63%) of them provide in-service training on various aspects of quality ranging from IPC to hand hygiene.

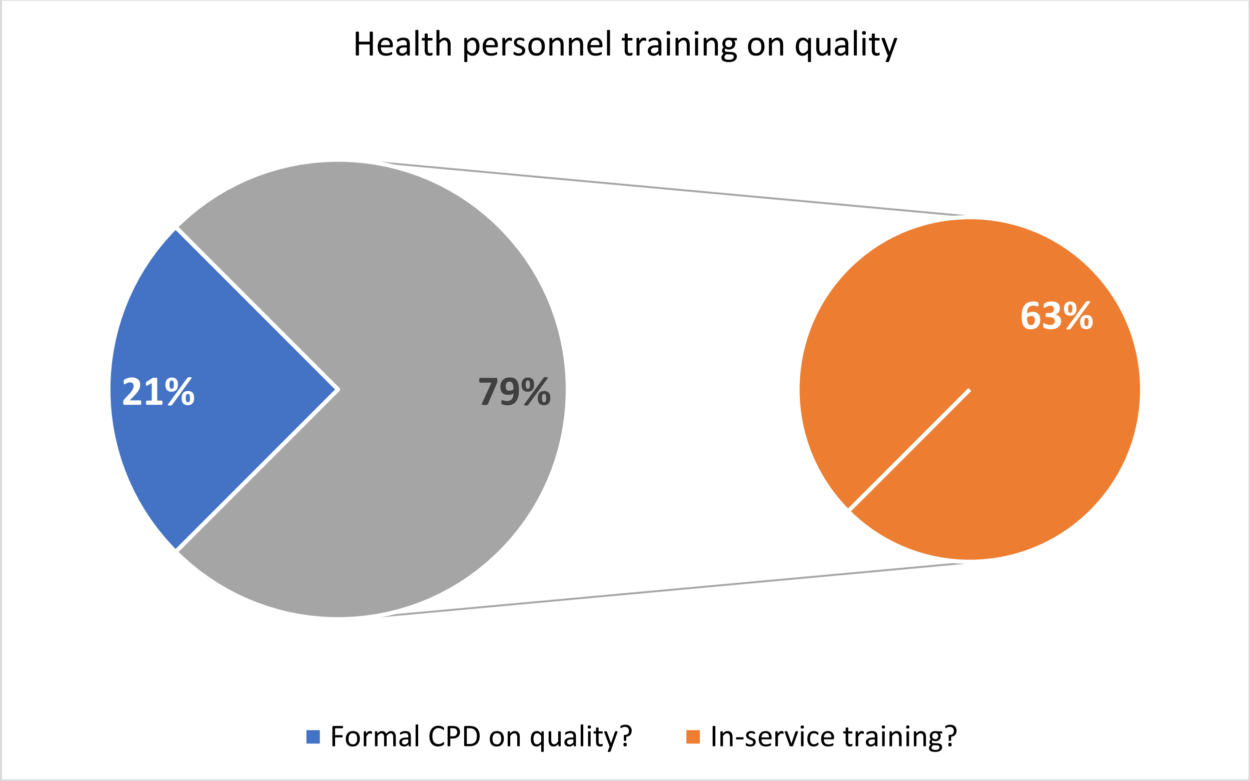

Figure 19: Proportion of health facilities in which personnel undergo some form of training in QI

A similar picture was reflected in training of health personnel pertaining to emergency preparedness and response. Just 16% of health facilities had personnel who were undertaking or had undertaken a CPD in emergency.

Regarding capacity building activities in the health facilities for emergency preparedness and response, the organization of Simulation Exercises (SimEx) and participation in After Action Reviews (AARs) were questioned.

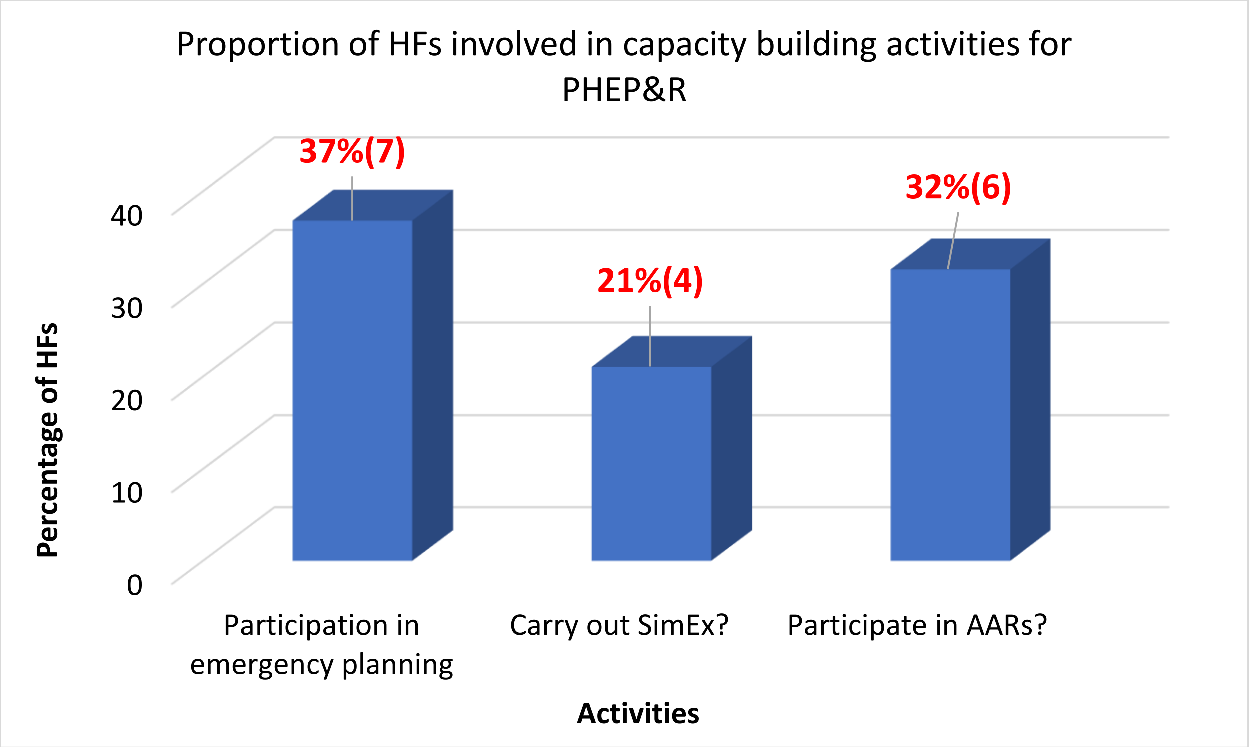

Figure 20: Health facilities involvement in SimEx, AARs

From figure 22 above, just four health facilities reported having organized a SimEx as part of emergency preparedness activities in recent time. Also, only 36% of the facilities have participated in an AAR in the past or recent times. Most of the respondents (63%) declared that their facilities are not involved in the planning phase of emergency response, be it at the county or national levels. SimEx and AARs are very important activities in preparing the health workforce for emergency response.

Another key element in organizational capacity was the availability of relevant national strategies or guidelines for quality or emergency. The assessment found that all of the respondents in health facilities have never heard about or seen a copy of the Liberia National Quality Strategy and the National Action Plan on Health Security in Liberia. Very few of the facilities were in possession of an Emergency Preparedness and Response Plan, Blood Safety guideline and the National Laboratory System policy. These are all key publications which elaborate action plans and SOPs relevant for emergency preparedness and response. The guidelines for malaria and mental health were available in almost all of the facilities visited as shown in the figure below.

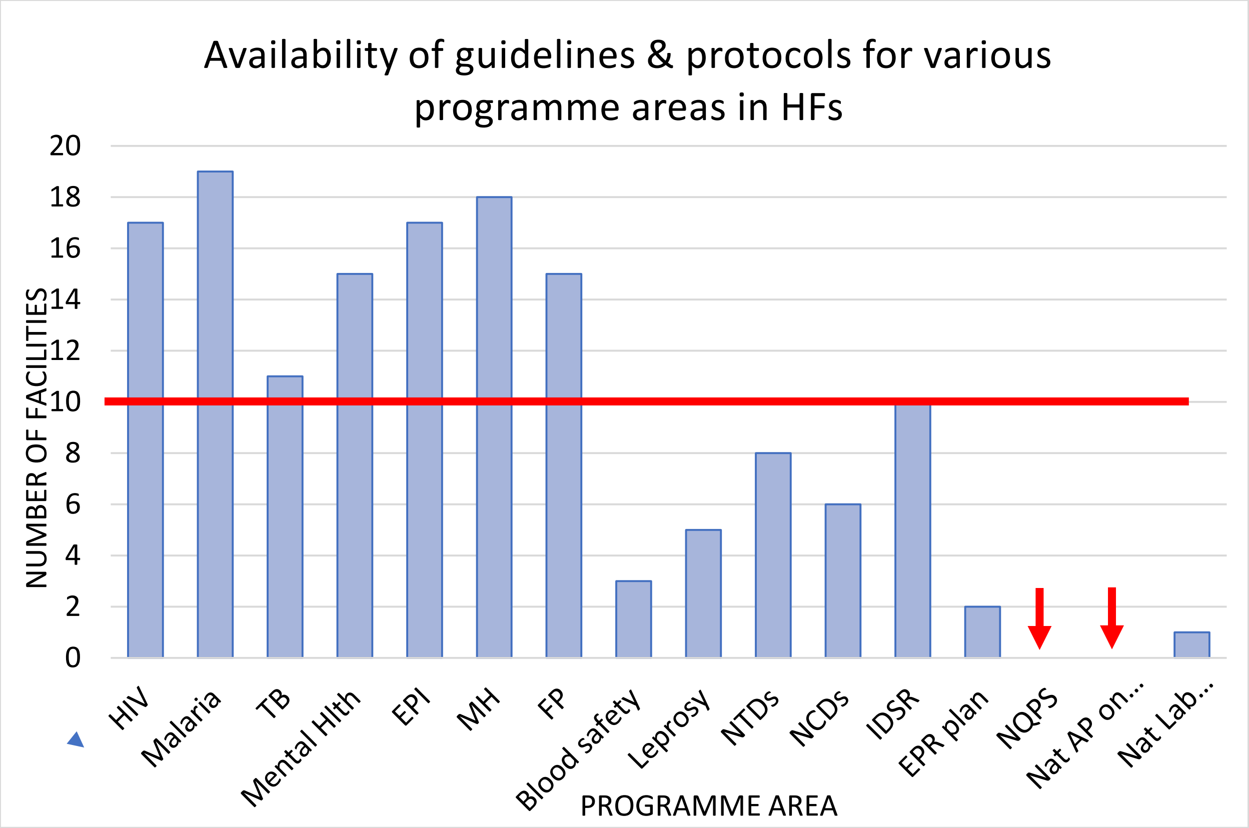

Figure 21: Availability of strategic documents for quality and emergency at health facility level

5.8. Integration of quality and emergency preparedness

The existence of a framework or link between human, animal and environmental health is crucial in tackling public health emergencies, especially disease outbreaks. From the findings, there appeared to be a weak bond between human, animal and environmental health at the health facility level. Just 6 health facilities were reported to integrate the One Health trio (human-animal-environment) through periodic meetings with officials from the veterinary and environmental sectors.

On the other hand, there was a clear understanding from respondents on the relevance of applying QI methods to improve emergency response. For example, the practice of proper hand hygiene is crucial in the response to Lassa, EVD outbreaks, among others. Also, the use of checklist and protocols will improve the diagnostic capacity of laboratories for diseases under surveillance.

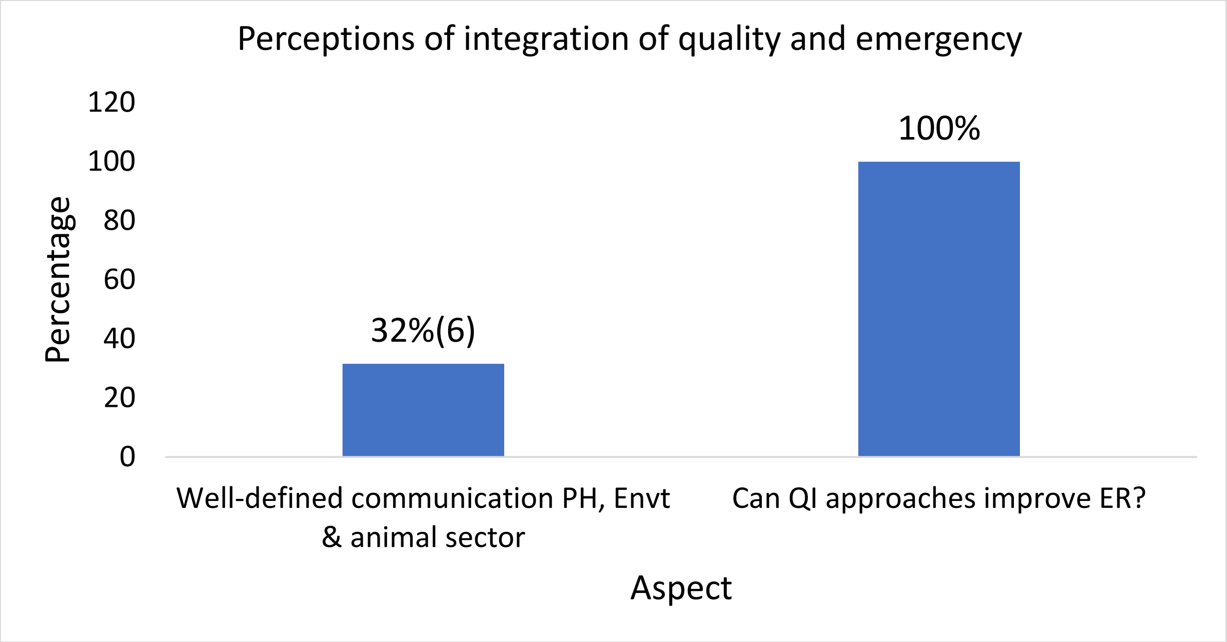

Figure 22: Perception of an integrated model of care for resilience

Overall, there were a number of aspects for which all respondents gave very positive remarks. For example, there was a warm reception of the project approach of linking quality and emergency preparedness and response at the facility level using the catchment approach. Also, all of the health facilities benefit from routine supportive supervision from the county or district health teams.

6. Levers, barriers, gaps and opportunities for integrated approach to building health service resilience

6.1. Levers

- There are national legislations, policies and plans which support the objectives of the project;
- Political/leadership will to implement EPR/IDRS/IHR at national and subnational levels;
- Prioritization of health sector emergency preparedness and response in national investment plan;
- Collaboration between MoH research team, NPHIL, pre-service training institutions, regional laboratory established,
- Evidence on benefits to integrate quality and emergency preparedness in routine health services,
- Potential partners for collaboration are available both at national and subnational levels,
- Experience of human health surveillance training courses at University of Liberia (FETP) can guide other training programs (IPC, WASH, AMR etc),
- Availability of an electronic integrated monitoring and measurement tool for data collection and supervision,
- Some VHF isolation and management capacity built in the field already,
- Existing QI practices: IPC, AMR and WASH practices, supervision and coaching.

6.2. Key challenges and gaps

Workforce capacity

- Critical shortage of skilled health workforce
- Limited capacity of PoE health staff on quality (IPC, AMR stewardship, waste management) and emergency (contact tracing, use of PPEs)
- Most health workers in routine healthcare lack skills in SimEx and AARs
- Limited in-country capacity for the integration of health quality and emergency preparedness
- Weak research capacities to monitor, evaluate and report on quality and resilience
- Inadequate capacity in-country on quality improvement in the health system

System functionality

- Poor quality and safety culture in health care delivery
- Insufficient funding for quality improvement and emergency preparedness and response
- Poor intersectoral coordination on quality and resilience

- Parallel planning and implementation of activities between health care quality and preparedness
- Poor working environment and motivation for health workforce
- Limited functionality of the QMT and RRTs at the health facility level
- Poor road network and difficult access to many health facilities, especially during the rainy season,
- Frequent disease outbreaks including Lassa, meningitis, and other vaccine preventable diseases (measles, pertussis), coupled with persistent high maternity mortality rate
- Limited knowledge and experience-sharing platforms on quality and emergency
- Limited referral capacity within routine health service delivery system

Training and capacity building

- Insufficient emphasis of quality and emergency preparedness and response in pre-service training,
- Limited resources and skills to conduct SimEx,
- Absence of systematic training modules on quality and emergency for CPD of health workers,
- Unavailability of stand-by roster for RRT
- Limited engagement of port health authorities & health facilities

Diagnostic capacity and supplies

- National Reference Laboratory and regional labs not functioning to optimal capacity
- Frequent stock of laboratory reagents and supplies
- Poor laboratory capacity at facility level to detect diseases under surveillance
- Regular stockout of IPC and WASH supplies in health facilities
- Inadequate support to port of entries (POEs); inadequate screening at POEs;
- Absence emergency funds for timely response to disease outbreaks

Information and data management

- Poor harmonized data management systems e.g. the Integrated Human Resource Information system (IHRIS), E-IDSR
- Failure of the IHRIS of the MOH

Behavioral change to integrated approach

- Territorial interest: most programs and sectors of the health system still function in parallel and restrict financial resources and even skills to benefit their areas of work only,

- Donors’ focus interest is paramount in most projects in which donors push for their specific objectives and goals to be met, irrespective of the activities of other actors in the field,
- Most interventions are donor-driven, and will usually fold up after the duration of the project set by the donors has elapsed,
- Fragmentation/vertical approach to implementation,
- Unmatched capacities across sectors (animal health, environmental health and human health),
- Poor adherence to triage processes at some health facilities,
- Issues with sustainability of the project after donor funding has expired.

6.3. Opportunities

- **Strong commitment and political will** to improve health service quality and emergency preparedness as part of initiatives for building a resilient health system at national and subnational levels. This determination has been reflected in the national health policy and plan (2011-2021), National Human Resource Policy and Strategic Plan (2011-2021), the Investment Plan for Building a Resilient Health System (2015-2021). The purpose for this commitment is to build a resilient health system and restore the gains lost to the EVD crisis between 2014-2016. Also, to provide health security for the people of Liberia by reducing risks due to epidemics and other health threats and accelerates progress towards UHC by improving access to safe and quality health services as well as ensuring access to safe and quality essential medicines. A robust health emergency risk management system, and an enabling environment that restores trust in the government’s ability to provide services is required to build health system resilience. The revised Public Health Law (2019); NAPHS (2018), NPHIL Strategic Plan (2018) and the National Quality Strategy (2018-2022) all demonstrate a strong political will to improve quality of routine health care and emergency response.
- **Establishment of the Health Quality Management Unit** mandated to improve health service quality in collaboration with other programs, agencies and partners provide an enabling environment for integrating health service quality improvement initiatives and emergency preparedness into the national health system. This is evidenced by the nomination of focal persons from both MOH and NPHIL who are currently supporting the implementation of the KOICA project in close collaboration with WHO Country Office.
- **Establishment of the NPHIL with surveillance structures at national and subnational levels** (e.g. disease prevention and control unit, district and county surveillance officers, health facility surveillance focal persons). The project can leverage on the existing structures with integration of health service delivery and emergency preparedness efforts across the three sectors (human, animal and environment).

- **Current decentralization initiatives by MOH and other line ministries** demonstrated by the NHPP (2011-2021), Decentralization Policy and Strategy (2011) of the health delivery system, including establishment of county, district, facility and community health management structures with county health boards. This project can leverage on the decentralization process to collaborate with MoH, NPHIL, and other line ministries including Agriculture, Veterinary and Environment to strengthen quality and emergency preparedness efforts at subnational levels.
- T**he integration of quality elements (e.g. IPC, AMR, WASH) and emergency preparedness into the One Health *approach*** *provides an opportunity for the project to* strengthen the interlinkages between health services, public health and animal health at all administrative levels.
- **Existence of RRT structures at national and subnational levels** are important developments in the county which can be encouraged and leveraged to improve the involvement of health facilities in the preparedness for emergencies and effective participation in response without compromising the routine health services.

7. Limitations

- The situational assessment was successfully conducted in three counties. However, we cannot claim that the desk review was comprehensive. This is because some of the key documents were not available on the MOH website, especially current data on health workforce. This was because the electronic Human Resource Information System (HRIS) was shut down.
- Besides, the review team experienced some delays from some stakeholders from whom relevant reports or documents were requested. Until the time of compilation of this report some grey literature that was requested from certain institutions had not been provided despite follow ups and reminders.
- There were multiple versions of some information in different reports or documents reviewed. The team had to decide on which narrative to adopt. This could have left out some relevant information in the process.

8. Conclusion

The health system of Liberia having suffered a number of public health shocks is in need of interventions geared towards strengthening its healthcare delivery and emergency response structure and functions. The KOICA project seeks to use quality improvement in routine healthcare as an entry point to strengthen emergency preparedness and response in health facilities. The country situational assessment carried out in 19 health facilities in three counties was intended to shed light on the structures, systems, challenges and opportunities that exist to build a resilient health system in Liberia with quality and emergency.

Besides the challenges identified, the assessment also exposed many opportunities for integrating quality and emergency response within the health system of Liberia. This project is therefore a timely and appropriate approach to strengthen the health system of Liberia. To achieve the objectives set out by the project a careful and purposeful selection of interventions which are relevant and feasible for the context of Liberia is key. The hallmarks of the stakeholder's consultative meeting were:

1. List of target health facilities for project implementation confirmed;
2. Initial project interventions, support and modalities endorsed;
3. Stakeholders for project implementation identified and engaged and the integrated approach advocated with positive feedback from stakeholders;
4. Salient aspects of discussions to finalize the situational assessment report captured.

The report highlights the need for resilient national and subnational or local level health service delivery to prevent, detect, respond to and recover from public health events in Liberia, which is achievable through an integrated approach to quality health service provision and emergency preparedness. This further requires commitment of all actors in creating the enabling environments for implementation and sustainability.

9. Recommendations from the stakeholders' consultation meeting

A three-day stakeholders' consultative meeting brought together about 45 stakeholders from the MOH, NPHIL, LMDC, LBNM, donor and partner organizations, WHO Country Office-Liberia, AFRO-Inter-country support team, and WHO-HQ, to validate the findings from the situational assessment and agree on package of support for the project. At the end of the meeting the following recommendations were made to the project team and key partners (MOH, NPHIL and WHO);

9.1. On tools and measurement approaches

- **Select a set of indicators** from the existing indicators found in diverse measurement tools in the MoH and NPHIL that can be used to monitor resilience in the health system,
- **Develop a standardize integrated tool** for periodic assessment of QoC in routine service delivery and emergency preparedness and response,
- Form **multi-disciplinary governance team** at the central level to oversee and coordinate the use of measurement tools, data collection, data analyses and feedback to health facilities on QoC and emergency preparedness,
- **Jointly mobilize and share of resources** between the QMU/MOH, NPHIL and relevant partners to enhance ownership and ensure sustainability of an integrated approach to quality and emergency,
- **Establish and strengthen multi-disciplinary Quality Management Teams** (QMTs) at the sub-national and facility levels.

9.2. On health facility reporting on quality and emergency

- Build capacity of health personnel at health facilities to apply the SPAR C9 (chemical, radiation, contaminations, etc),
- Provide adequate equipment to health facilities to detect and respond to public health threats,
- Institutionalize real time reporting for public health emergencies at the health facility level,
- Strengthen county capacity to monitor and report SPAR-C9 activities,
- Improve cross-border coordination by training PoE health workers on QoC and emergency response techniques
- Increased coordination and partnership with strong and high-level political commitment

10. Project next steps based on Stakeholders' Consultative Meeting

1. Develop and share summary of the three-day consultative meeting report.
2. Finalize the draft Situational Assessment Report based on the inputs from the stakeholders' consultation.
3. Share the ToRs and subsequently work plan for Health Service Resilience Training and Indicators package to interested experts and authorities e.g. professional and academic bodies in Liberia.
4. Project Team to work with authorities in first wave facilities to confirm the arrangement for support.
5. Undertake joint review of activities for next 6 months (TWG).
6. Train the country project teams, including MoH and NPHIL to conduct Health System Resilience SimEx in Liberia.
7. Start implementing the package of support with MoH, NPHIL, local partners and authorities at facilities level.

Reference

World Population Prospects. (2019 Revision). United Nations population estimates and projections. Retrieved July, 15, 2019 from http://worldpopulationreview.com/countries/liberia-population/

Country Economy. (2018). Liberia GDP 2018 Retrieved July, 5, 2019 from https://countryeconomy.com/gdp/liberia.

The World Bank (2019). World Bank in Liberia. Retrieved July, 5, 2019 from https://www.worldbank.org/en/country/liberia/overview

United Nations Development Program (2018). Human Development Indices and Indicators, statistical updates.

World Health Organization. (2018). Country cooperation strategy for Liberia

United Nations Development Programme. (2018). Human development statistical update. Retrieved July, 15, 2019 from http://hdr.undp.org/sites/default/files/2018_human_development_statistical_update.pdf

Liberia Ministry of Health. (2018). Service Availability Readiness Assessment (SARA) report.

Brolin Ribacke, K. J., Saulnier, D. D., Eriksson, A., & von Schreeb, J. (2016). Effects of the West Africa Ebola virus disease on health-care utilization–a systematic review. Frontiers in public health, 4, 222.

Kruk, M. E., Gage, A. D., Arsenault, C., Jordan, K., Leslie, H. H., Roder-DeWan, S., ... & English, M. (2018). High-quality health systems in the Sustainable Development Goals era: time for a revolution. The Lancet Global Health, 6(11), e1196-e1252.

World Health Organization. (2017). Community engagement frameworks for quality, people-centered and resilient health services. Geneva: World Health Organization

An Roinn Sainte. (2013). How to conduct a literature search. Internal staff guide.

Liberia Ministry of Health (2015). Investment plan for building a resilient health system 2015-2021. Republic of Liberia.

Liberia Ministry of Health. (2016). Joint Annual Health Sector Review.

Republic of Liberia. (2018). Joint National Action Plan for Health Security 2018-2022.

Liberia Ministry of Health. (2019). Five-year strategic plan for the national laboratory system of Liberia, 2019-2024, draft

Liberia Ministry of Health. (2018). Health Sector Performance Evaluation Report (2006-2017). Republic of Liberia.

Republic of Liberia (2015). 2015/2016-health workforce census in Liberia.

Liberia Ministry of Health (2018). Health Sector Performance Evaluation Report (2006-2017). Republic of Liberia

Liberia Ministry of Health (2018). Health Sector Performance Evaluation Report (2006-2017). Republic of Liberia

World Health Organization (2019). Liberia Country Support Plan, 2019-2020.

US Center for Disease Control. (2019). Ebola Virus Disease-Case count. Retrieved July, 5, 2019 from https://www.cdc.gov/vhf/ebola/outbreaks/2014-west-africa/case-counts.html.

Liberia IDSR Epidemiology Bulletin. (2017). Epi Week 39 (September 25 – October 1, 2017).

World Health Organization. (2015). SARA. An annual monitoring system for service delivery Reference Manual; Version 2.2 Revised July 2015.

Liberia Ministry of Health. (2013). EPHS accreditation final report.

Liberia Ministry of Health. (2017). Joint Integrated Supportive Supervision Tool, Revised 2017.

Liberia Ministry of Health. (2013). EPHS Accreditation Final Report.

Cleveland, E. C., Dahn, B. T., Lincoln, T. M., Safer, M., Podesta, M., & Bradley, E. (2011). Introducing health facility accreditation in Liberia. *Global Public Health*, *6*(3), 271-282.

World Health Organization. (2016). International Health Regulation Join External Evaluation Report

World Health Organization (2018). WHO Liberia Country Office Report.

World Health Organization (2019). Global mapping of simulation exercise materials: a health system review, technical report.

Liberia Ministry of Health. (2018). Simulation Exercice report

National Bridging Workshop. (2018). IHR and the OIE-PVS Pathway

Bastiaensen, P. et al. (2016). OIE-IHR-PVS gaps analysis mission report, Republic of Liberia

Ministry of State for Presidential Affairs. (2015). Cabinet Guide for Ministries, Agencies and Commissions, Cabinet Secretariat, Republic of Liberia.

World Bank and OECD. (2018). Delivering quality health care: a global imperative

World Health Organization (2006). Quality of Care: a process for making strategic choices in health systems

ANNEX

Annex I: List of desk reviewers

SN	Name	Position and Organization
1	Dr. Catherine Cooper	Assistant Minister, Curative Health Services, Ministry of Health
2	Dr. J. Ngormbu Ballah	Director, Healthcare Quality Management Unit, Ministry of Health
3	Hon. Henry A. Blake	Deputy Director General for Administration, NPHIL
4	Dr.Lekilay G. Tehmeh	Clinical Coordinator & Patient Safety, HQMU, MoH
5	Nelson Dunbar	Research Director, MOH
6	Josephus Kilikpo	Research Unit, Ministry of Health
7	Culleen Parker	Research Unit, Ministry of Health
8	Mike Mulbah	M & Director, Ministry of Health
9	Patrick Konwloh	Health Information Management System (HIS) Director, MoH
10	Roseline George	Surveillance Coordinator, NPHIL
11	Nathaniel Dovillie	Emergency Preparedness Focal Person, NPHIL
12	Mrs. Edna Kiawion	Child, Adolescent and Nutrition Officer, WHO
13	Moses B. Bolongei	Technical Lead, KOICA Project AMR Officer, WHO - Liberia
14	Mohammed Kromah	GIS Specialist, WHO
15	Dr. Louis Ako-Egbe	WHO Consultant
16	Edna J. Kiawoin	Child, Adolescent and Nutrition Officer, WHO-Liberia
17	Dr. Sohel Saikat	KOICA Overall Project Lead, Geneva, Switzerland
18	Redda Seifeldin	Consultant, KOICA Project

Annex II: Summary of relevant literature reviewed with findings concerning the status of quality and emergency

No.	Title of documents	Year	Focus on QOC	Focus on EPR	Reference to an Integrated approach	Scale
**Policies, Regulations and legislations**						
1	Revised Public Health Law of Liberia	2019	√	√	√	3
2	Liberian Board for Nursing and Midwifery State Board Examination Policy	2019	√	X	X	1
3	National healthcare quality policy statement	2018	√	√	X	2
4	Liberia National Blood Safety Policy*	2018	√	√	X	2
5	National Policy and Strategic Plan on Health Promotion, 2016-2021	2016	√	√	√	3
6	Information Communication Technology (ICT) Policy and Strategy (2017-2021)	2017	√	√	√	3
7	National Disaster Management Policy	2012	√	X	X	1
8	National Health and Social Welfare Decentralization Policy and Strategy	2012	√	X	X	1
9	National Health and Social Welfare Policy and Plan, 2011-2021	2011	√	√	X	2
10	National Health and Social Welfare Financing Policy and Plan (NHSWPP) 2011 – 2021	2011	√	√	X	2
11	National Human Resources Policy and Plan (HRPP) for Health and Social Welfare 2011-2021	2011	√	X	X	1
12	National-Environmental-and-Occupational-Health-Policy	2010	√	√	X	2
13	Medicines & Health Products Regulatory Authority (LMHRA) Act	2010	√	X	X	1
14	By-Laws & Constitution of the Liberia Medical and Dental Council	2010	√	X	X	1
15	An Act to Establish the Ministry of Health	2010	√	√	X	2
16	Code of conducts for Health Professionals in Liberia	2012	√	X	X	1

No.	Title of documents	Year	Focus on QOC	Focus on EPR	Reference to an Integrated approach	Scale
17	National Health Infrastructure Policy (NHIP&P)	2010	√	X	X	1
18	National Monitoring and Evaluation Policy for Health	2009	√	X	X	1
19	Institutional Review Board Policies & Procedures Handbook	2008	X	X	X	1
20	National Drug Policy	2001	√	X	X	1
21	The Public Health Laws (1976)	1976	√	X	X	1
**Plans and Strategies**						
22	Disease Specific Contingency Plans (Cholera, Lassa Fever, Yellow Fever and EVD)	2019	√	√	X	2
23	Five-year Strategic Plan for the National Laboratory System of Liberia, 2019-2024*	2019	√	√	√	3
24	The National Water, Sanitation and Hygiene Commission Strategic Plan, 2019-2024*	2019	√	√	√	3
25	Joint National Action Plan for Health Security (NAPHS)	2018	√	√	√	3
26	National Action Plan on Prevention and Containment of Antimicrobial Resistance in Liberia, 2018 – 2022	2018	√	√	√	3
27	National One Health Strategic Plan, 2019-2023	2018	√	√	√	3
28	National Public Health Institute of Liberia Strategic Plan, 2017-2022	2018	√	√	√	3
29	National Public Health Institute of Liberia 2019 Annual Work Plan	2019	√	√	X	2
30	National Risk Communication Plan, 2017-2019	2017	√	√	X	2
31	Liberian Health Information System and ICT Strategic Plan, 2016-2021	2016	√	√	X	2
32	National Epidemic Preparedness and Response Plan	2016	√	√	X	2
33	Consolidated Operational Plan-Fiscal Year, 2019-2020	2016	√	√	√	3

No.	Title of documents	Year	Focus on QOC	Focus on EPR	Reference to an Integrated approach	Scale
34	Investment Plan for Building a Resilient Health System	2015	√	√	√	3
35	Revised National Community Health Services Strategy and Plan, 2011-2015	2011	√	√	X	2
36	Revised National Community Health Services Strategy and Plan, 2016-2021	2011	√	√	√	3
37	Liberia National Health Quality Strategy, 2017-2021	2017	√	√	X	2
**Guidelines**						
38	National Guidelines for the Accreditation of Nursing and Midwifery Programs in Liberia	2019	√	X	X	1
39	National guidelines for the safe management of healthcare waste*	2019	√	X	X	1
40	National Infection Prevention and Control Guidelines	2018	√	√	√	3
41	2nd Edition National Standard Therapeutic Guidelines and Essential Medicines List Liberia	2017	√	X	X	1
42	National Technical Guidelines for Integrated Disease Surveillance & Response (IDSR)	2016	√	√	x	2
43	Cabinet Guide for Ministries, Agencies and Commissions	2015	X	X	X	0
44	Liberia Ebola Virus Disease Clinical Management Manual	2014	√	X	X	1
45	Guidelines for the Setting up and Operating National and Hospital Pharmacy and Therapeutic Committee in Liberia	2012	√	X	X	1
**Reports**						
46	Hospital Verification Report: Q Assessment and Quantity Varication, Quarter 1-2019	2019	X	X	√	1
47	Electronic Joint Integrated Supportive Supervision Pilot Review Meeting Report	2019	√	√	X	2
48	Advanced infection prevention and control training pilot: Liberia training report	2019	√	X	X	1
49	Semi-annual counter verification report for the period ended; January to June, 2019	2019	√	X	X	1

No.	Title of documents	Year	Focus on QOC	Focus on EPR	Reference to an Integrated approach	Scale
50	World Health Organization Country Office Annual Report 2019	2019	√	√	X	2
51	Animal Diseases Surveillance System and Response in Liberia	2019	√	√	X	2
52	National Bridging Workshop on the International Health Regulations (IHR) and the Organization of Anima Health (OIE) Performance of Veterinary Services (PVS) Pathway	2018	X	√	X	1
53	Health sector resource mapping report	2018	√	√	√	3
54	Health Sector Performance 2006-2017 Evaluation Report	2018	√	√	√	3
55	Vulnerability Risk Assessment and Mapping Planning Workshop Report	2018	X	√	X	1
56	Simulation Exercise Report (Maryland)	2018	√	√	X	2
57	Healthcare Quality Management Unit Annual Report	2018	√	X	X	1
58	Evaluation Report on the Implementation of the "One Health" Approach in the West African Region	2018	√	√	X	2
59	Integrated Disease Surveillance and Response Supportive Supervision Reports, 2017-2018	2017/18	√	√	X	2
60	After Action Reviews (ARR) Report: Lassa Fever, Meningococcal Meningitis	2017/18	√	√	√	3
61	Service Availability and Readiness Assessment (SARA) Reports, 2016 and 2017	2016/18	√	X	X	1
62	Twinning Partnership for Improvement, Report	2017	√	X	X	1
63	Epidemic Preparedness and Response Consortium Isolation Facility Assessment Summary Report	2016	√	√	X	2
64	Joint Annual Health Sector Review Report 2016	2016	√	√	√	3
65	Republic of Liberia: Joint External Evaluation of IHR core capacities	2016	X	X	X	0
66	Safe and Quality Health Services (SQS) Training Program External Evaluation Report	2016	√	√	X	2
67	Republic of Liberia MoH Joint Financial Management Assessment Report	2016	√	√	X	1

No.	Title of documents	Year	Focus on QOC	Focus on EPR	Reference to an Integrated approach	Scale
68	Infection Prevention and Control End of Year Report, 2015, 2016	2015	√	√	X	2
69	Essential Package for Health Services (EPHS) Accreditation Final Report	2013	√	X	X	1
**Others**						
70.	Essential Package for Building Health Services	2011	√	X	X	1
71.	Liberian Board for Nursing & Midwifery Republic of Liberia Competency-Based Curriculum Post-Basic Bachelor of Science in Midwifery (BSc)	2019	√	X	X	1
72.	Public Health Emergency Operations Center PHEOC (PHEOC) handbook	2019	√	√	X	2
73.	Action Brief: Integrating infection prevention and Control into national directions on quality in Liberia	2019	√	√	√	3
74.	One Health Governance Manuel	2018	√	√	√	3
75.	State Party Self- Assessment Annual Reporting Tool	2018	√	√	√	3
76.	GHSA & IHR Standardized Milestone Library	2017	√	√	√	3
77.	National Health Accounts Fiscal Years, 2013-2014; 2015-2016	2013/15	√	√	X	2
78.	Competency-Based Curriculum Pre-service Training for: Registered Nurses and Midwives, Environmental Health Technicians and Physician Assistants	2011	√	√	X	2
79.	Liberian Board for Nursing & Midwifery Republic of Liberia Competency-Based Curriculum CM to RM Bridging Program	2011	√	√	X	2

Annex III: Health Facility Assessment Tool

**PROJECT SITE SITUATIONAL ASSESSMENT TOOL**<br>***Making Health Services Resilient with Quality and Preparedness for Emergency Response***			***WHO-KOICA Project:***
A	**Health Facility Assessment**		
	**County:** **Assessment date:**		
**1**	**Facility details**		
	Name of Facility:	**Location (GPS coordinates)**	
	District:	**Latitude:**	**Longitude:**
	Position of respondent:		
	Sex of Respondent:		
	**Facility Type**		
	a. Clinic		
	b. Health Center		
	C. Hospital		
	**Catchment Population**	**#________________**	
	**Facility Ownership**		
	a. Public		
	b. Private-for-Profit		
	c. Private-not-for profit		
2	**Staffing**	**Number**	**Comments**
	a. General Medical Doctor		
	b. Specialist Doctor		

	c.PA		
	d. Mental Health Clinician		
	e. Dentists		
	f. Lab Tec		
	g. Eye Nurses		
	h. Midwives		
	i. Registered Nurse		
	j. Pharmacist		
	k. Dispensers		
	l. Lab Aide		
	m. CHA		
	n. Nurse Anesthetic		
	o. Other, specify		
3	**Services provided/programs**	**Yes/ No**	**Comments**
	a. HIV		
	b. Malaria		
	c. TB		
	d. Mental Health		
	e. EPI		
	f. Maternal Health		
	g. Family Planning		
	h. Blood Safety		

	i. Leprosy			
	j. NTDs			
	k. NCDs			
	l. Others-specify			
4	Has the facility experienced staff attrition or turnover in the last 6-12 months?	If yes, indicate number of staff attrition (if known)		# of Staff:
	New staff during the last 6-12 months	**Yes**	**No**	# of Staff:
	a. General Medical Doctor			
	b. Specialist Doctor			
	c. Nurse Anesthetist			
	d. PA			
	e. Midwives			
	f. RN			
	g. Other Specify			
**B**	**Quality of Care: Availability of Strategy, plans, SOPs or guidelines for quality health care**			
	**Questions**	**Yes/No**		**Probe/comments**
1	Health facility managers are aware of relevant national strategy/plans/guidelines documents relating to preparedness and quality of care *(which documents is mostly known and which ones are not)*			Probe for locally available strategies, guidelines, SOPs, protocols etc
1.1	If yes, select all documents that applies	**Yes**	**No**	**Verified**
	a. HIV			
	b. Malaria			
	c. TB			

	d. Mental Health			
	e. EPI			
	f. Maternal Health			
	g. Family Planning			
	h. Blood Safety			
	i. Leprosy			
	j. NTDs			
	k. NCDs			
	l. IDSR 2016			
	m. Emergency Preparedness or EPR Plan			
	n. Liberia National Quality Strategy			
	o. National Action Plan on Health Security			
	p. National Laboratory System Policy			
	q. Others, specify			
2	Does the facility have functional quality management /improvement team			If yes, list composition of this team
2.1	Does the facility have designated focal person responsible for quality improvement activities?			If yes, ask for TOR/work plan
3	Are there ongoing funded projects being implemented on quality improvement in this facility?			If yes, list projects, duration and supporting partners
4	Are there formal continuing professional development (specify training programs) on quality improvement for maintaining staff competency in this facility?			If yes, list training programs or training modules available
5	If no, are there occasional in-service training for quality improvement for health workers?			If yes, list type of training, category of staffs trained

6	Are there existing measurement tools being used to monitor quality of healthcare services at health facility level?			If yes, kindly list available tools (JISS tool, checklist, data collection forms, ledgers, registries)
7	Is feedback on synthesized reports and information from data submitted provided to health facility staff?			if yes, are feedback submitted on: QI or emergency response/both, how often feedback is provided?
8	Has the facility been accredited by any national regulatory body/professional association e.g. LMDC, LBNM?			If yes, accrediting Inst./year
9	Does the facility have basic infrastructure for quality health care delivery?			if yes, Probe for electricity supply, water supply, placenta pit, incinerator etc
10	Does the health facility have adequate laboratory capacity to detect and report cases of common diseases and other diseases with epidemic potential?			If yes, what diagnostic and surveillance tools are available for investigating and reporting diseases?
11	Does this facility have the capacity to use diagnosis tool available?			If yes, state training/skill acquired
12	If no, does the facility have links with the reference laboratory at the county or national level?			If yes, kindly list the reference laboratories.
**C**	**Emergency Preparedness and Response**			
1	Does this facility have a functional emergency response team?			If yes, list the composition of this team?
2	Does the facility have designated focal person responsible for emergency preparedness and response activities			If yes, ask for TOR/work plan
3	Does this facility have an emergency preparedness and response plan?			If yes, verify copy of this plan
4	Is the facility involved in regional or district emergency preparedness and response planning?			If yes, how is this facility involve
5	Are there formal continuing professional development (specify training programs) on public health emergency and response for maintaining staff competency in this facility?			If yes, list training programs or training modules available

6	If no, are there occasional in-service training for public health emergency and response for health workers?			If yes, list type of training, category of staffs trained
7	Does the facility carry out simulation exercises (SimEx) as part of emergency preparedness?			If yes, list type of exercises, who was involved and time
8	Does this facility participate feedback sessions/ reviews (AAR)?			If yes, state last date of participation
9	Are there existing measurement tools which are being used to monitor public health emergency at health facility level?			If yes, kindly list available tools (checklist, data collection forms, ledgers, registries, JISS tool)
10	Are there ongoing funded projects being implemented on emergency preparedness and response in this facility?			If yes, list projects, duration and supporting partners
11	Is there any funded project on health service resilience with an integrated focus on quality and preparedness at this facility			If yes, provide list projects, duration and supporting partners
12	Are there well defined communication links between the health facility, public health and animal health sectors?			If yes, describe the process for communication during emergencies
13	Doses the facility have the capacity to diagnose any of the below diseases	**Yes**	**No**	
	a. Malaria			
	b. HIV			
	c. TB			
	d. Cholera			
	e. Others Specify			
**C**	**General Questions**			
1	Are health authorities interested in participating in the project?	Yes	No	If no, why?
2	What percentage of your catchment population falls within 5 km/one-hour distance from the facility?	Percentage:____		
3	Are the catchment communities of this facility involve with facility planning exercises?			If yes, how are they involved? (minutes of last meeting held)

4	Does this facility undergo routine supervision and support from district, county and national levels team?			If yes, verify the last visit recorded in visit book
5	In your opinion, can quality improvement techniques and routine health services be applied to improve emergency preparedness and response			Please explain
6	What do think are possible challenges to successful implementation of quality in emergency preparedness and response?	Probe: how can these challenges be addressed		

Annex IV: Initiatives to strengthen health services quality and emergency preparedness since 2015

No	Initiative name	Policy/plan linkage	Area of focus	Responsible organizations	Location (County, District, HF)	Duration / Status
**Initiatives in the area of health services quality**						
**1.**	Integrated Management of Childhood Illnesses (IMCI)	-Essential Package for Health Services (2011);<br>-National Health and Social Welfare Policy and Plan (2011-2021);<br>-National Community Health Services Policy (2016-2021)	• Maternal and child health<br>• Pre-service training of Midwives and Lab officers<br>• Appropriate infection control practices<br>• Vaccinations<br>• Early recognition and home management of illness	MoH, WHO, USAID, JHPIEGO, UNICEF	Health facilities in 15 counties	Ongoing
**2.**	Integrated Community Case Management (ICCM);	-National Health and Social Welfare Policy and Plan (2011-2021);<br>-President's Malaria Initiative: Malaria Operational Plan (2018); Revised national community health services strategy and plan (2011-2015)	• Community-based healthcare –children<br>• Improving the quality of malaria diagnostic testing through on-site training and supportive supervision in all 15 counties.<br>• Strengthening of the health information system, and improved human resource management.	MoH, WHO, G2G; UNICEF; USAID; US-CDC	Health facilities in 15 counties	Ongoing
**3.**	Infection Protection & Control (IPC) education and training	-National Infection Prevention and Control Guidelines for Liberia (2018),<br>-National Action Plan on Antimicrobial Resistance (2018-2022**);**<br>-National action plan for health security (2018-2022)	• IPC training (Keep Safe, Keep Serving, Safe & Quality Health Services, Advanced IPC training and National IPC Guidelines<br>• Supplies<br>• Supervision	MoH, WHO, G2G, CDC, JHPIEGO, GIZ, PIH, ACCEL	Health facilities in 15 counties	PC guidelines training is ongoing

No	Initiative name	Policy/plan linkage	Area of focus	Responsible organizations	Location (County, District, HF)	Duration / Status
**4.**	WASH in health facilities	-National Health Policies and Plans, NHPP (2007/2011, 2011/2021);<br>-Investment Plan for building resilient health system (2015-2021);<br>-HRH Policy & Strategy Plan (2007-2011, 2011-2021);<br>-National health infrastructure policy (2010)	• Training<br>• Supplies<br>• Monitoring	MoH, WHO, CDC, GIZ, UNICEF	Health facilities in 15 counties	Ongoing
**5.**	Maternal, new born and adolescents' health service delivery	**National Health**<br>-NHPP (2007-2011, 2011-2021);<br>-Investment Plan for building resilient health system (2015-2021)<br>-RNMCAH Investment case	• Training<br>• MNDSR<br>• Mentorship/supervision<br>• In-service training for adolescent health, mid-level workers (PA, Nurses, Midwives)	WHO, UNICEF, UNFPA, World Bank Health Portfolio	Montserrado (3 health facilities in 2 districts);<br>Grand Bassa; County; Phebe; CB Dunbar; Tellewoyan	Ended (2018)
**6.**	Quality emergency obstetric & newborn care (EmONC), post-natal care (PNC) & Child Health	-NHPP (2007-2011, 2011-2021); -Investment Plan for building resilient health system (2015-2021);<br>-HRH Policy & Strategy Plan (2007-2011, 2011-2021);<br>-National health infrastructure policy (2010)	• Infrastructure<br>• Diagnostics	World Bank, MoH	Redemption Hospital, JFK	FY 20/21
			• Upgrade of health facilities<br>• Procurement of equipment	GIZ, MoH	Fishtown and Redemption Hospitals, Esther Bacon School of Nursing and Midwifery	FY 20/21
			• Direct nutrition interventions to Integrated Management of Acute Malnutrition	UNICEF, MOH, WHO	Health facilities in 15 counties	Ongoing
			• Installation, maintenance & repair of solar devices in public & private health facilities.	GIZ	Health facilities in 15 counties	FY 20/21

No	Initiative name	Policy/plan linkage	Area of focus	Responsible organizations	Location (County, District, HF)	Duration / Status
			• Procure and install cold room freezers monitoring device and voltage stabilizer	GAVI	Central Medical Store (CMS)	FY 18/19
			• Procure & distribute commodities for malaria interventions<br>• Technical assistance for supply chain management for HIV, Malaria and Family Planning commodities	USAID	Health facilities in 15 counties	FY 18/19
			• Construction of new EPI storage capacity (i.e. Dry & cold) at National & County	GAVI	Health facilities in 15 counties	FY 18/19
7.	Equipping health facilities with skilled providers	-NHPP (2007-2011, 2011-2021); -Investment Plan for building resilient health system (2015-2021);<br>-HRH Policy & Strategy Plan (2007-2011, 2011-2021);<br>-National health infrastructure policy (2010)	• Pre-service education for midwives & laboratory technicians	USAID, MoH, and regulatory bodies	Tubman National Institute of Medical Arts, Mother Patern College of Health Sciences, United Methodist University, Esther Bacon School of Nursing & Midwifery, Phebe Nursing School and MTPSER)	FY 19/20
8.	Quality reproductive, maternal, newborn, child and adolescent	-NHPP (2007-2011, 2011-2021); -Investment Plan for building resilient health system (2015-2021);<br>-HRH Policy & Strategy Plan (2007-2011, 2011-2021);	• Pre-service education for midwives & laboratory technicians<br>• EPHS implementation in Bong, Lofa, and Nimba	GFF/World Bank Health Portfolio; USAID/FARA; WE Care Solar;	Redemption, Phebe, CB Dunbar, Tellewoyan Memorial, Jackson F	FY 19/20

No	Initiative name	Policy/plan linkage	Area of focus	Responsible organizations	Location (County, District, HF)	Duration / Status
	health (RMNCAH) service provision	-National health infrastructure policy (2010);<br>- RMNCAH investment case,	• Quality facility-and community-based health services<br>• IPC supplies, pre-service education for midwives & laboratory technicians?	GIZ; UNFPA;<br><br>*FARA/USAID & WB not supporting pre-service	Doe, FJ Grant Hospital);<br>PBF at primary level (Gbarpolu, Sinoe, River Cess)	
**9.**	Equipping health facilities with skilled providers	-NHPP (2007-2011, 2011-2021); -Investment Plan for building resilient health system (2015-2021);<br>-HRH Policy & Strategy Plan (2007-2011, 2011-2021);<br>-National health infrastructure policy, National Immunization Policy (2012)	• Support medical education training for family medicine residents at JDJ and Para-medics<br>• Clinical mentorship and education training	Human Resources and Services Administration (HRSA), MoH	Faculties under Liberia College of Physicians (LCPS), AMD, GMRP faculty consortium), Tubman University, JDJ	FY 18/19
			• In-service training of health workers (vaccinators and CMs) on immunization in practice<br>• Supportive supervision in targeted counties	GAVI	600 health facilities, offering EPI services plus 50 total trainers	FY 18/21
			• Pre-service training at the post-graduate (Masters) level	MoH, GFATM,	Mother Pattern and Phebe	FY 20/21
**10.**	Adolescent health	-NHPP (2007-2011, 2011-2021); -Investment Plan for building resilient health system (2015-2021);<br>- HRH Policy & Strategy Plan (2007-2011, 2011-2021)<br>- ASRH	• Training and mentoring healthcare providers on comprehensive post abortion care	CHAL, MoH, WHO, WB, USAID/FARA	Grand Bassa, Grand Kru, River Gege, River Cess, Sinoe, Gbarpolu and Montserrado	FY 20/21
**Initiatives in the areas of emergency preparedness**						
**11.**	EPR and surveillance with	-NHPP (2007-2011, 2011-2021;	• Integrated service delivery and quality improvement-	MoH, GFATM, WHO, CDC	Health facilities in 15 counties	FY 20/21

No	Initiative name	Policy/plan linkage	Area of focus	Responsible organizations	Location (County, District, HF)	Duration / Status
	emphasis on maternal newborn death surveillance and response (MNDSR)	-Investment Plan for building resilient health system (2015-2021);<br>-HRH Policy & Strategy Plan (2007-2011, 2011-2021);<br>-National health infrastructure policy	laboratory systems for disease prevention and control, treatment and disease surveillance<br>• Disease & event-based surveillance			
**12.**	Animal health (One health	-National One Health Strategic Plan (2018-2022),<br>-Investment plan for building resilient health system (2015-2021),<br>-NPHIL Strategic Plan (2018)	• Surveillance<br>• Diagnostics<br>• Curriculum development<br>• Training	MoH, USAID: FAO/ECTAD	Health facilities in 15 counties	Ongoing
**13.**	Bio-surveillance of wild viruses	-Animal disease surveillance and response plan (2018);<br>-Investment plan for building resilient system (2015);<br>-One health strategic plan (2018)	• Bio-surveillance of wild viruses<br>• Training	MoH, USAID: Predict2 (Eco-Health Alliance)	Lofa, Nimba , Montserrado, south-east etc.	Ongoing
**14.**	Infectious diseases detection & surveillance (IDDS)	-Investment plan for building resilient system (2015);<br>-One health strategic plan (2018)	• Surveillance<br>• Laboratory capacity building (One health approach)<br>• AMR capacity building (Lab)	MoH, USAID, WHO, CDC	Public referral hospitals laboratories, National Reference Laboratory	Ongoing
**15.**	AMR Surveillance	-NAPHS (2018-2022),<br>-AMR National Action Plan (2018-2022),<br>-One Health Strategic Plan (2018)	• AMR sentinel	MoH, WHO, CDC, GIZ	South eastern counties	Ongoing
**16.**	E-IDSR	-Investment plan for building resilient health system (2015-2021),<br>-One Health Strategic Plan (2018),<br>-NAPHS (2018)	• Pilot	MoH, CDC	Margibi & Grand Cape Mount	Complete d

No	Initiative name	Policy/plan linkage	Area of focus	Responsible organizations	Location (County, District, HF)	Duration / Status
**17.**	Field epidemiologist program	-Investment plan for building resilient health system (2015-2021),<br>-One Health Strategic Plan (2018),<br>-NAPHS (2018-2022)	• Training of Field epidemiologist	NPHIL/MoH;REDISSE, World Bank, CDC	73 districts across Liberia 15 counties	Ongoing
**18.**	IHR Core Capacity	-Investment plan for building resilient health system (2015-2021),<br>-One Health Strategic Plan (2018),<br>-NAPHS (2018-2022)	• Development of Multi-hazard preparedness and response plan, surveillance, laboratory services, IHR in PoE zoonosis, risk communication<br>• Supportive supervision & monitoring<br>• Supporting implementation of AMR national action plans	WHO, FAO, IOM, UNDP; US-CDC	15 counties (national and subnational	Ongoing

Annex V: Summary of key institutions/stakeholders and their role in quality & emergency

Stakeholder	General roles	Role in quality and emergency preparedness of health services
**National and sub-national government**	**National:** The national governance of Liberia includes the cabinet that consists of the President, Vice-President, and 28 Members (Ministers, Directors-General and senior Advisors to the President[46]);<br>**County:** includes county superintendents, district commissions, paramount, clan and town chiefs and zonal heads.<br>Responsible for approval of legislations and policies, coordination, developing government's strategic direction and decision making at highest level (national and county), monitoring implementation of government priorities, managing delivery of government services[47].	1) Responsible for approval and monitoring implementation of legislation, policies, plans and strategies related to quality of health services and emergency preparedness at national and subnational levels.<br>2)Responsible for cross-sectorial coordination of stakeholders in both public and private sectors<br>3)Responsible for declaring a state of national disaster based on recommendations from the National Disaster Management Agency (NDMA) and MoH
**Ministry of Health (MoH)**	Provides leadership and governance of the health system, through development and review of health policies, guidelines, standards and plans, partners coordination, resource mobilization, health financing, health workforce development, procuring supplies/equipment, infrastructure, health management information, promoting private-public partnership, advising other ministries and agencies on health-related matters, monitoring and evaluation of health programs	1)Responsible for planning, implementing, monitoring and evaluation of health system quality initiatives through its Health Quality Management Unit (HQMU) and partners<br>2)Collaboration with NPHIL and other sectors to develop and strengthen public health emergency prevention, preparedness and response at all administrative levels
**National Public Health Institute of Liberia (NPHIL)**	-Complements and supports MoH activities at all levels; including Environmental and Occupational Health, Disease Prevention and Control, National Reference Laboratory, and Biomedical Research functions<br>-Mandated to improve the Liberia's public health status in collaboration with relevant agencies and government	1)Institutionalization of operational system to facilitate early/timely detection, diagnosis and response to epidemics, emergencies and disasters at the national, county and community levels (emergency preparedness; including rapid response mechanism<br>2)Providing technical assistance, resources, coordination and logistical support to counties, public health workforce capacity building;

[46] Ministry of State for Presidential Affairs. (2015). Cabinet Guide for Ministries, Agencies and Commissions, Cabinet Secretariat, Republic of Liberia.
[47] Ibid

	institutions, in alignment with IHR core capacities (prevention, detection, and response to public health threats and events).	conducting and coordinating research; strengthening the health system to prevent, detect, confirm and respond to public health threats for sustainability and resilience
**National Disaster Management Agency (NDMA)**	-NDMA is an autonomous body that assumes overall coordination of disaster response in close consultation with the National Disaster Management Committee (NDMTC) and its sub-committees<br>-Both work closely with lead agencies on specific hazards which provide recommendations and technical expertise for NDMA and NDMTC to organize disaster risk reduction programs.	1)Responsible for coordinating disaster preparedness and response for natural and man-made hazards<br>2)Strengthening the government's institutional and operational response capacities in disaster risk reduction, national Early Warning System, risk transfer and integration of DRM into government post-disaster recovery plans<br>3)Increasing cooperation and collaboration between health and other sectors (e.g. agriculture, water, energy, law enforcement, transport, migration, foreign affairs and trade) before, during and after all types of health emergencies; raising public awareness and communicating risk
**Ministry of Education (MOE) and The National Commission on Higher Education in Liberia (NCHE)**	-MoE is mandated to ensure quality *education* for all, and overseeing the whole education sector<br>-It draws up policies, regulations, standards, strategies, plans and curriculum for educational reform and development; and supervises their implementation, coordination and management in all forms of formal education at various levels.<br>-NCHE is responsible for regulation of higher education (Colleges, Universities etc)	1)Provides oversight for health workers' pre-service education, including accreditation and regulations<br>2)Responsible for collaborations with MoH to ensure pre-service, in-service and CPD curriculums for healthcare workers training are up to date and of high quality<br>3)Responsible for monitoring, evaluating and accrediting all institutions of higher learning for healthcare workers to ensure that the requisite knowledge and skills are obtained
**Other ministries and agencies at the national and subnational levels e.g. Internal Affairs; Agriculture; Labor; Gender, Children and Social Protection; Youth and Sports; Civil Service Agency, Transport, Liberia Water and Sewage Corporation,**	Other line ministries are part of the governance structure at national and subnational levels	1)Key actors in ensuring continued collaboration with the MoH, NPHIL and partners, providing non-clinical human and material resources, infrastructure and services essential for quality routine and public health emergency services<br>2Responsible for participating in the One Health platform to provide relevant support for emergency preparedness<br>3)Civil Service Agency is responsible for health workforce employment, salary scales, employment regulations

**National WASH Commission etc**		
**County Health Teams (CHT); County Health Boards (CHB) and District Health Teams (DHTs)**	-The CHT serves as the linkage between the national and lower administrative levels of the health system; where some primary, secondary and tertiary hospitals (regional and county referral hospitals) have direct accountability to the CHT.<br>-Responsible for coordination of all activities at the health facility level including planning, resource allocation, financial management and implementation of national priorities, facility management, maintenance and supervision, management of personnel, collection and analysis of HMIS-generated data, coordination with local and international partners at their level, and collaboration with the County Health Board.	1) It Influences quality health service delivery and emergency preparedness by ensuring that health facilities have and implement appropriate operational and technical capacities (including equipment, supplies and infrastructure necessary for quality service delivery and IPC; collecting and analyzing data from districts, reporting to the national level and providing information back to the districts; leading outbreak response within the county.<br>2) CHT monitors quality improvement activities through joint integrated supportive supervision and feedback; hand hygiene compliance monitoring; maternal and neonatal death audits; disaster risk monitoring and mitigation; disease prevention and control.
	**County Health Boards:**<br>-The County Health Board comprises the superintendent, CHO, civil society and provides oversight for implementation of the National Health Policies and Plans within the context of the County Health Plan, engage and coordinate with stakeholders and cultivate accountability	1) Assist the County Health Team in multi-sectorial coordination; mobilize resources for the implementation of the county health plan which is essential in making available quality healthcare and emergency preparedness and response activities.
	**District Health Teams (DHTs)**<br>-They are the administrative link between healthcare facilities and the county health team.<br>-The district receives reports from healthcare facilities in its catchment area and submits reports to the county level<br>-The district health officer (DHO) serves as the administrative head of health services in a district and oversees district-level implementation of quality health services and emergency preparedness activities.	1) The DHT closely works with all healthcare facilities within the district in ensuring health service delivery through quality improvement, quality assurance, quality control and emergency preparedness/response measures and coordination of contribution from different stakeholders and partners<br>2) The district health team through the district data clerks and district surveillance officers are responsible for collecting and analyzing data from healthcare facilities, reporting to the county level, and feedback to healthcare facilities on IDRS priority diseases, conditions and events;

		3) DHO leads and coordinates the district rapid response team that is responsible for case management, including IPC, and WASH, working with health facility OICs
**Healthcare facilities (public and private) and healthcare workers**	-These are institutions with outpatient or in-patient facilities, including primary, secondary and tertiary healthcare facilities.<br>-Health service providers include, but not limited to: doctors, registered nurses, midwives, laboratory professionals, pharmacists, mental health clinicians, management team etc.	▪ Responsible for frontline health service provision, directly interacting with clients, patients, family caregivers, and communities to deliver preventive, promoting, palliative health care, curative, while considering quality principles and emergency preparedness<br>▪ Emergency preparedness functions includes, planning, surveillance, reporting, outbreak investigation and response, also supporting and supervising Community Health Assistants and Volunteers
**Healthcare service users and communities**	• The users of health services generally include: patient groups, clients, community members-who all have the right to quality, safe health care as well protection from health emergencies.<br>• Civil societies as part of the community contribute to promoting the rights of the community members	• They play major role in partnering with healthcare providers to ensure accountability, feedback, patient and community engagement, and participation for ensuring the provision of people centered quality universal health coverage as well as emergency preparedness
**Community Health Workers**	• This includes General Community Health Volunteers (gCHVs), Community Health Assistants (CHA), Trained Traditional Midwives (TTMs)	• The gCHVs, CHA, TTMs etc. play major roles in community-based surveillance and notification of healthcare facilities<br>• Also, important in providing health education, and promoting quality health care through basic healthcare services and referral of patients to appropriate health care facilities for further management
Academic and Professional Bodies		
**Academic (Public and Private) and research institutions**	▪ The role of research and academic institutions includes education, research and capacity building for various professions	▪ They are key players in training healthcare workers, largely in pre-service and certification, building health professionals' knowledge, and skills essential for quality health care and emergency preparedness<br>▪ Have a major role in generating evidence to improve operational performance for health service delivery and emergency

**Professional institutions (Medical, Nursing and Midwifery, Laboratory, Pharmacists, etc.)**	• Professional bodies (Liberia Medical and Dental Council, Liberia Board for Nursing and Midwifery, Pharmacist Board, Laboratory Board etc) monitor compliance with set standards and ethics of the profession, licensing of qualified professionals and accreditation of training programs	▪ Responsible for supporting the implementation of quality health services through curriculum development, ethics, mentoring, and regulations
**Private sector**	▪ Charities, faith-based organizations, nongovernmental organizations, and private providers have been major contributors to the health delivery system, with at least 38 percent of health facilities are privately owned	▪ The private sector is a growing source of health services including hospitals, clinics, and pharmacies.<br>▪ Responsible for working closely with MoH to ensure that quality health services are provided according to guidelines and standards, and to support scale up of services and contribute to emergency preparedness and response
International partners and local partners		
**African CDC**	• Key actor in providing technical support to strengthen public health capacities in member countries	▪ Key role in strengthening NPHIL surveillance capacities and partnerships to detect and respond to public health threats
**International and local NGOs and other UN agencies working in health-related sectors**	▪ UN agencies and NGOs closely collaborate with and support the government priorities, based on their mandate, resources and capacity	• Key actors in supporting health system strengthening through provision of technical assistance, financial and material resources for quality improvement and emergency preparedness interventions<br>• Also support capacity building, advocacy and research
**Donor agencies supporting quality of care and competency/health system resilience: KOICA, GIZ; USAID; GAVI**	• They provide funding and technical support to government and partners for various areas of national priorities; promoting aid effectiveness and harmonization with the international community	• Key actors in supporting health service quality and emergency preparedness through providing funding for related proposals from the government and partners in the country

**WHO (HQ, Regional and Country Offices)**	▪ Lead organization responsible for coordination, providing technical, financial, material support to MoH and partners in health-related matters, including policy, guidelines, monitoring and evaluation, medical supplies, research and technologies, technical capacity strengthening,	▪ Responsible for supporting national and sub-national level MoH and NPHIL in building capacity and systems strengthening for improving health services quality and emergency preparedness including IHR<br>▪ Improving global knowledge exchange on technical areas related to health emergencies and quality of care
