# Supplementary material for: Liberia health system's journey to long-term recovery and resilience post-Ebola: a case study of an exemplary multi-year collaboration
Source: Front Public Health. 2023 Jun 19;11:1137865. doi: 10.3389/fpubh.2023.1137865 (PMC10317185; doi:10.3389/fpubh.2023.1137865)
Supplement: Supplementary file 2 [file Table_1.DOCX]

**Annex to the paper: Situational analysis of the state of healthcare quality and service resilience in the Liberia health system**

A situational assessment (SA) conducted to inform project planning and implementation showed that there were national legislations and policies to support initiatives in building health services resilience by improving quality of essential services and emergency preparedness. Although quality and emergency preparedness are generally considered separately in national policies and plans, there was a national drive for an integrated approach to strengthen resilience of the health system to public health emergencies. The 2015-2021 Health System Resilience Investment Plan emphasized the integration of health services quality and emergency preparedness as essential elements for building resilience. However, this integrated approach was yet to be well developed and embedded within health system and health security initiatives aiming to strengthen health services. Considerations of emergency preparedness were also found to anchor mostly at the administrative levels, indicating the need to increase focus on health services-level preparedness.

The situational analysis also found that there was a lack of integration in measuring quality and emergency preparedness in health services. The efforts of quality measurements were mostly centered around EVD-based efforts and mostly related to IPC and, to an extent, maternal and child health (MCH). The National Health Quality Strategy (2017-2021) was developed to facilitate the achievement of the goals established in the National Health Policy and Plan (2011-2021) but lacked an M&E framework and was yet to be disseminated to all health facilities. Key strategic resources were absent, including standard tools/questionnaires to assess health system resilience, standards for health facility accreditation, a dedicated health system SimEx (Simulation Exercise) package, a training package for health system resilience (HSR), sets of indicators for quality and resilience, guidelines for Health Service Continuity Planning and for Continuing Professional Development (CPD).

In addition, the analysis highlighted the need for a systematic and integrated approach in improving the capacity of health workers and health system leaders in quality and resilience, and ensuring their active participation in activities related to health service quality and preparedness for public health emergencies. Areas for improvement included emergency preparedness and service continuity planning, integrated approach to training and measurement, quality management structures, interlinkages with public health, animal and environmental health sectors.
